# High-Spin Porphyrin Polyradicals

**DOI:** 10.1021/acsomega.5c10439

**Published:** 2026-01-05

**Authors:** Sergi Betkhoshvili, Jordi Poater, Ibério de P. R. Moreira, Josep Maria Bofill

**Affiliations:** † Departament de Química Inorgànica i Orgànica & IQTCUB, 16724 Universitat de Barcelona, Martí i Franquès 1−11, Barcelona 08028, Spain; ‡ Departament de Ciència de Materials i Química Física & IQTCUB, Universitat de Barcelona, Martí i Franquès 1−11, Barcelona 08028, Spain; § ICREA, Pg. Lluís Companys 23, Barcelona 08010, Spain

## Abstract

We propose metal-free, open-shell porphyrins with at least full diradical or tetraradical characters, high-spin ground states, and highly delocalized unpaired electrons obtained via minimal modifications of porphine. Since many functional (bio)­organic compounds lack sufficient pro-aromatic parts to allow seamless aromaticity-induced polyradical design, we impose the lower-bound number of unpaired electrons by tailoring the topology of π-conjugation. The presented method, Topologically Rational Assembly of Polyradicals (TRAP), is useful for designing multifunctional magnetic compounds for organic electronics, spintronics, biosensors, single-molecule devices, etc. The TRAP method transcends classes of π-conjugated compounds, thus allowing the design of polyradicals from bioorganic conjugated systems and the tuning of their magnetic properties.

## Graphical abstract

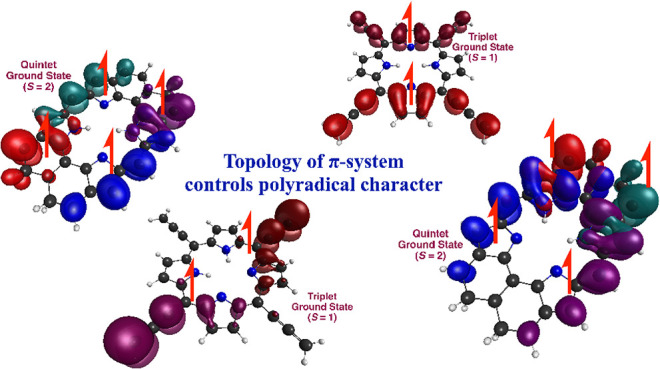

## Introduction

Polyradicals due to unique electronic and magnetic properties have applications in organic electronics, spintronics, organic magnetic materials, molecular switches, bioimaging, photodynamic therapy, quantum technologies, etc.^1–11^ Despite extensive studies of diradicals and upcoming studies of conjugated compounds with more than two unpaired electrons,^2,12–22^ for the overwhelming majority of fully π-conjugated polyradicals, the open-shell electronic structure is a consequence of aromatic stabilization (e.g., Tschitschibabin’s and Müller’s hydrocarbons^23,24^ ) or 4*n* (antiaromatic, open-shell) or 4*n* + 2 (aromatic, closed-shell) π-electrons in the ring according to Hückel’s rule.

There have been few fully π-conjugated polyradicals designed based on bioorganic compounds, because fully π-conjugated frameworks with sufficient (pro)­aromatic subsystems properly connected to allow easy design of polyradicals are very rare. Furthermore, naively connecting several radical groups to the conjugated π-system is not guaranteed to result in the proper structure of the polyradical with the desired number of unpaired electrons and ground-state (GS) multiplicity. In recent works, we established that the principal factors controlling the polyradical character, GS multiplicity, and structure of the low-energy spectrum are aromatic stabilization and the topology of π-conjugation.^25–28^ We proposed a theory of rational design of fully π-conjugated organic polyradicals with control over GS multiplicity and low-energy spectrum,^27^ the method henceforth named as Topologically Rational Assembly of Polyradicals (**TRAP**). In this work, we apply this method to design high-spin porphyrin polyradicals and present structures that are more optimal in terms of minimal structure modification and a higher number of resulting unpaired electrons than attaching the maximum amount of *radicalogen groups* to the π-system of porphine. In fully π-conjugated systems, we define a *radicalogen group* as a chemical group that is connected to the starting conjugated system via a σ bond, is conjugated to the original π_*z*_ system, and has an unpaired electron (distributed) in the *p*_*z*_ orbital(s) (e.g., –ĊH_2_). The presented strategy can be used to design open-shell compounds from different classes of (bio)­organic compounds by rationally adjusting their structure, which affects the topology of their π-system and electron density distribution.

Depending on the topology of the fully conjugated π-system, there is a specific number of maximum allowed *proper* (between σ-bonded atoms) π bonds (*N*_π–*b*_(max)). If there are more available electrons *N*_e_ than this maximum number of π bonds requires, then there are at least *N*_e‑unp_(min) = *N*_e_ – 2*N*_π–*b*_(max) unpaired electrons. We proposed this general definition for topological restriction in our work.^27^ The special case of this in polycyclic aromatic hydrocarbons (PAHs) is called *nullity*, denoted as η, which shows how many unpaired electrons there are based on the maximum number of nonadjacent vertices α and edges β of a PAH, based on the hexagonal graphs theorem,^29^ that is, η = α – β. As we systematically showed in our recent works,^26–28^ and has been repeatedly reported in the studies of diradicals, if one wants to increase the number of unpaired electrons without introducing the (new) topological restriction, then one needs to make sure there is a bridging group, directly conjugated to the unpaired electrons via the π-system, which switches the π-bond configuration in open-shell form with a gain of resonance energy of 2–3 benzene rings to offset the energy of one fewer π bond in the open-shell configuration compared to the closed-shell configuration, as exemplified by Tschitschibabin’s and Müller’s hydrocarbons.^23,24^ The further refinement of the concept of the topological restriction presented in this work enables us to design such porphyrin polyradicals with full diradical and tetraradical characters that have highly delocalized unpaired electrons. Multiple highly delocalized unpaired electrons in such a small chemical structure as the presented porphyrins become possible due to the careful analysis of topological and electronic effects that allow the control of the properties of designed compounds reliably.

## Results and Discussion

In this work, we apply the principles of the **TRAP** method to design delocalized high-spin porphyrin polyradicals. We also introduce and demonstrate a systematic refinement within the concept of topological restriction on a minimum number of unpaired electrons in the π-conjugated system in order to have greater insight into the control of the electronic structure of polyradical­(oid)­s. One must note that some of the electrons that are expected to be fully unpaired based on (for example) nullity criteria can engage in long-range on-bond pairing via through-space interaction. We henceforth call this effect the *loosening* of the topological restriction, which leads to reducing the open-shell character of the particular subshell that corresponds to these unpaired electrons within the open-shell π-subspace. We apply the concept of loosening topological restriction on the minimum number of unpaired electrons in porphyrin-based systems, as shown in Figure 1b in addition to the primary principles of the **TRAP** method to design delocalized high-spin porphyrin polyradicals.

**Figure 1:**
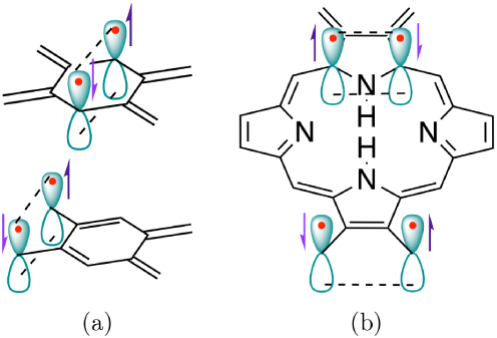
Orbital schemes for long-range π on-bond pairing in (a) tetramethylenebenzene and (b) tetramethyleneporphine.

### Tightness of Topological Restriction

Formally, multiconfigurational valence bond theory defines a set of valence bond configurations that can be mapped to the set of resonance structures. Thus, to simplify terms, we use *resonance structures* to refer to the representative Lewis structure drawings that contribute to the *resonance hybrid* and also the corresponding set of valence bond configurations that contribute to the multireference wave function of a given compound. To better explain the phenomenon of loosening the topological restriction, we can recall the nonzero contribution of “Dewar” resonance structures (RSs) in the ground-state wave function of benzene. We can postulate the possibility of analogical contribution of such RS ***d-k*** in the ground-state wave function for the reference compound **CB** shown in Figure 2a and in a series of compounds shown in Figure 3 sharing this central benzene ring (thus abbreviated as **CB**). Note that we denote the *para* π bond with a dashed line to distinguish this *improper* π bond from the proper π and σ bonds. We have computationally shown that the compound **CB** has about 60% diradical character, significantly lower than a pure diradical, in the Supporting Information of our recent work.^27^ This shows that the topological restriction has been loosened due to the contribution of RS of type ***d-k*** via long-range through-space on-bond pairing, as shown in Figure 2 with the orbital scheme in Figure 1a. Zero *tightness* of topological restriction means complete through-space on-bond pairing, which in planar organic systems is rarely ever achieved. Maximum tightness means the complete absence of through-space on-bond pairing.

**Figure 2:**
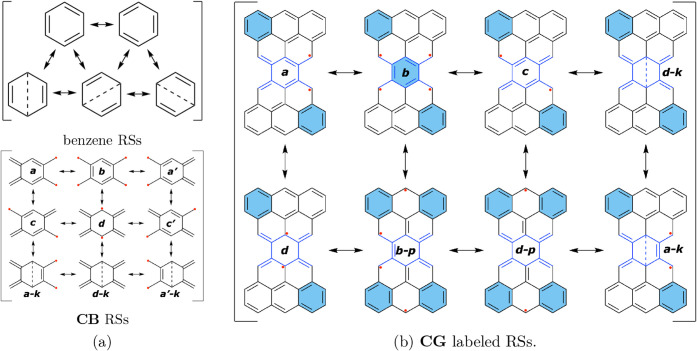
(a) Resonance structures of **CB** and (b) Clar’s goblet **CG** with Kekulé- and Dewar-type (dashed *para* π bonds) forms with labels showing topological homology to **CB**.

**Figure 3:**
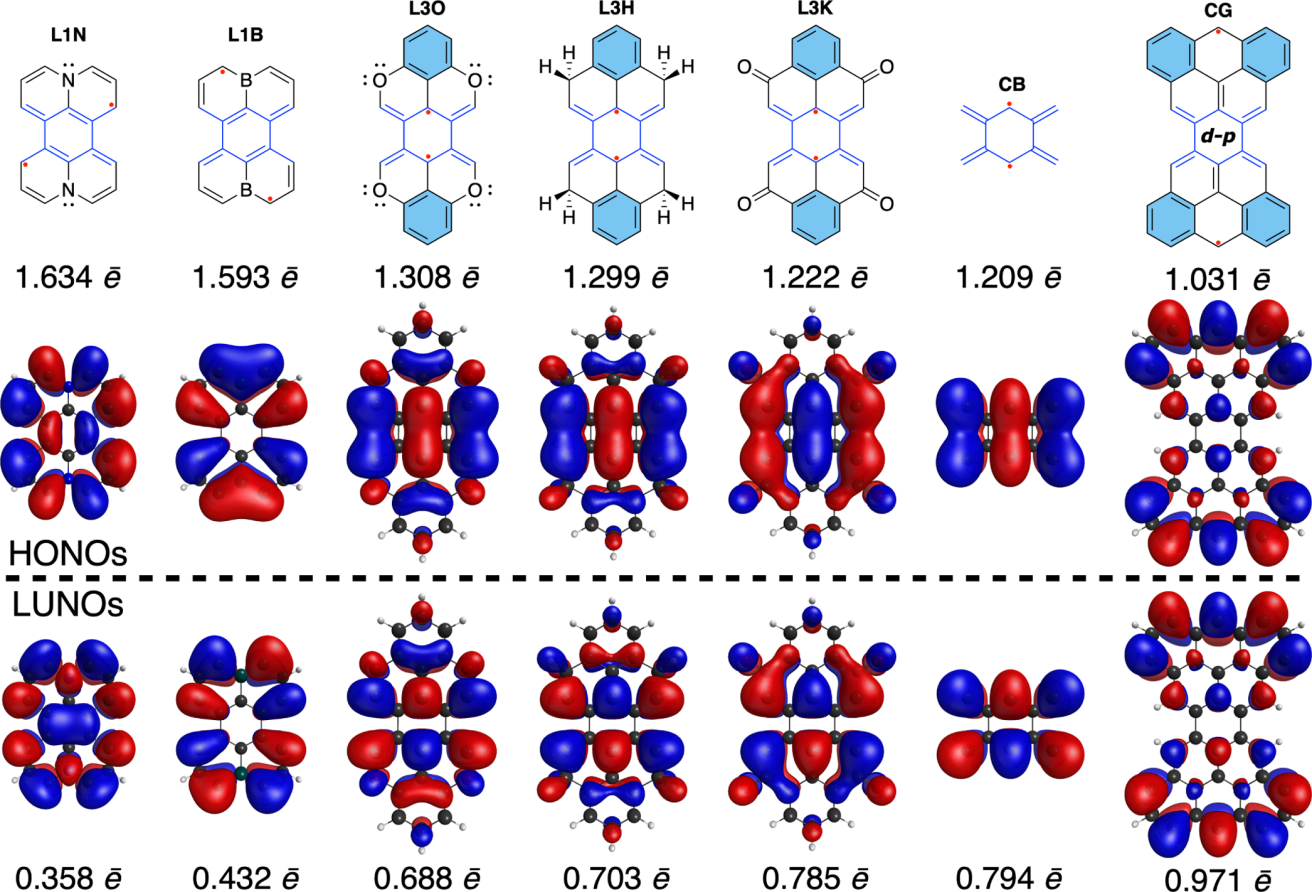
Modulation of tightness of topological restriction in compounds with the same central part but different total size and structure of π-conjugated systems (drawn with significantly contributing RSs). Frontier NOs are shown with occupation numbers.

To analyze how the tightness of this topological restriction changes, we can study the related species with the identical central topology of the π-system and the same apparent topological restriction on the minimum number of unpaired electrons, in the series of compounds shown in Figure 3. For these compounds, we define topological homology from the standpoint of the compound **CB**, as its structure is embedded in every compound and their topology of the π-system maintains the limiting features, such as the minimum necessary and maximum appropriate number of unpaired electrons. The compounds in this homologous series are designed such that the topological restriction of having two unpaired electrons comes from the same cross-conjugated **CB** unit. The favored resonance structure of the embedded unit in the larger π-system will change according to the appropriate complementarity to the π-bonding configuration in the surrounding groups. That is, to define at least one feature of homology with such an embedding, the larger structures must have a π-system configuration that is consistent with (at least one) resonance structure of the homology-defining embedded unit. This implies a proper π-system configuration at the interface between the embedded and surrounding units. Thus, the mode of complementarity of π-systems of the embedded unit and surrounding units might change depending on which of the resonance structures of the embedded unit are allowed or favored within the larger π-system. Nonetheless, in the set of resonance structures of the larger π-system, the subunit **CB**, which defines homology, can only have contributions from resonance structures that are consistent with RSs originally topologically allowed for it and those which are related to them by unpaired electrons delocalized into other atoms of a larger π-system. Note that compound **CG** in Figure 2b, which is a famous Clar’s Goblet, has been synthesized on the surface^30^ and in solution.^31^ The authors referred to an excellent explanation for the very high open-shell character of Clar’s goblet, since it is impossible to concomitantly pair all *p*_*z*_ orbitals to form π bonds, thus generating uncompensated radicals.^30,31^ This is in line with the topological analysis that we used in one of our previous works^27^ to provide the general solution of polyradical design in alternant systems (including PAHs) with addition rules between the (di)­radical subunits. Erich Clar, in his book *The Aromatic Sextet* ^32^ about polycyclic conjugated hydrocarbons, showed several versions of the topology of the π-system that would have the possibility of a *para* π bond, including higher acenes. Notably, the higher contribution of the RS(s) with the *para* π bond directly corresponds to the diminished tightness of topological restriction to have a specific minimum number of unpaired electrons due to the impossibility of forming more proper π bonds. Nonetheless, the tightness of topological restriction has been mostly overlooked in studies of extended π-conjugated systems, because in most of them the possibility of forming an *improper* π bond is attenuated by the delocalization of π-electrons.

We extend upon Clar’s analysis with our refinement by introducing the tightness of topological restriction, to estimate the collective contribution of *para*-bonded (or generally π-bonded between nonadjacent atoms) structures in the systems that Clar envisioned and many more that we proposed in our previous works.^25–28^ Such an estimation of trends does not require performing multiconfigurational quantum chemistry calculations. By using the tightness measure and understanding that it depends on the structure of the π-system, one can readily explain some of the qualitative open-shell properties. One of such properties is the reinforcement of the magnetic ordering and a significant singlet–triplet gap in Clar’s goblet, which was reported to be 0.29 kcal/mol,^31^ unexpectedly strong spin–spin coupling if one had assumed fully disconnected spin centers. In the limit when the principal conditions of our **TRAP** method rules^27^ are barely satisfied, long-range electron correlation effects can become important for designing extended π-conjugated polyradicals and predicting their qualitative properties reliably.

We performed electronic structure calculations on the compounds shown in Figure 3 using CASSCF­(14,14) (only for **CB**, CASSCF­(10,10)) on each of the limiting geometries obtained from restricted and unrestricted Kohn–Sham (RKS and UKS) density functional theory (DFT), optimized for closed-shell and triplet states. For comparative analysis, for each compound, we used the lowest CASSCF energy solution among the ones obtained for RKS and UKS triplet DFT-optimized geometries. Since the topology of the π-system in this homologous series of alternant compounds unequivocally defines the singlet ground state according to Ovchinnikov’s rule,^33^ it is not expected to have avoided crossings near and at the equilibrium geometries upon first-order perturbation. Thus, upon calculation with multireference methods, these DFT geometries are biased toward lower and higher diradical characters, but with a persistent singlet ground state as verified by CASSCF calculations. The open-shell character can be compared using occupation numbers (*n*_NO_) of the highest occupied and the lowest unoccupied natural orbitals (HONO and LUNO) given in Figure 3. Natural orbitals (NOs) are eigenvectors of the first-order density matrix operator, and their corresponding eigenvalues are natural orbital occupation numbers.^34^ In qualitative terms, a given NO shows the electron density distribution of the average number of electrons occupying this orbital, as given by the occupation number. The closer *n*_HONO_ and *n*_LUNO_ are to 1 (*n*_HONO_ + *n*_LUNO_ ≈ 2), the higher the diradical character *y*_0_, which varies from 0 to 1 (see Methods and Computational Details). The noteworthy case is when unpaired electrons are not allowed to make on-bond pairing by a through-bond path but they are spatially close, especially if they have opposite spins. If a spin-antiparallel configuration is consistent with through-bond coupling, then the contribution of structure(s) with through-space on-bond pairing can be enhanced. This **loosens** the topological restriction to have these electrons unpaired, but **does not remove** it. The consequence is a reinforced magnetic ordering and lowered open-shell character for this subsystem but nonetheless maintained open-shell nature. We use the frontier NO occupation numbers to estimate the open-shell character of the compounds in the series and the density distribution of unpaired electrons indicated by frontier natural orbitals to infer which diradical resonance structure(s) is (are) more important in the resonance hybrid. This is justified due to the partial but formally appropriate correspondence of one-particle representation of electronic structure using the frontier natural orbitals to characterize the density distribution of (possibly) unpaired electrons. Note that these frontier NOs show the density distribution of electrons that are unpaired in the diradical configuration and bond-paired in the closed-shell configuration. Thus, we can infer which (set) of the resonance structures would be consistent to contribute to such an electron density distribution of (possibly) unpaired electrons. Furthermore, we can use the Clar’s rule^32^ which considers aromaticity as a local property of the benzene-like six-membered rings with π-sextets that are separated from adjacent rings by C–C single bonds. Clar’s rule states that from the resonance structures with equal number of π bonds, the one(s) with most Clar’s π-sextets will contribute most to the resonance hybrid. If we examine the frontier NOs and correspondingly estimated *one of the representative RSs* of the compounds in the series, we see that the regions of the higher electron density are closer in the compounds with relatively lower open-shell character (looser topological restriction). The *para* π bond leading to the loosening of the topological restriction can be directly visualized by referring to the HONOs of **L3O**, **L3H**, **L3K**, and **CB** in Figure 3. There is a significant overlap between the electrons that are not allowed to make on-bond pairing via a through-bond π-conjugated path. Notably, this effect is amplified when these unpaired electrons are more confined in the center, as in **L1N** and **L1B**. Specifically, boron- and nitrogen-containing compounds are homologous to **CB** in topological limits, with nominally at least two unpaired electrons and with four as the maximum appropriate number of unpaired electrons due to the central cross-conjugated unit. The nitrogen-containing compound **L1N** has the loosest topological restriction because lone pairs of peripheral nitrogen atoms repel unpaired electron density toward the center. This causes the unpaired electron density distributions to have higher overlap, inducing stronger long-range on-bond pairing interaction. This effect is almost fully maintained in the boron-containing compound **L1B**. The difference is a longer carbon–boron bond than a carbon–nitrogen bond and boron having an empty *p*_*z*_ orbital, thus being an acceptor of the π-electron density. These factors allow unpaired electrons to be a little further than in the nitrogen-containing compound, in which nitrogen is a donor of π-electron density. The effect of confinement is partly maintained in **CB**, as there are several pairs of atoms that have significant unpaired electron density and are spatially distanced similarly to the *para* atoms in benzene. Nonetheless, contrary to the case for **L1N** and **L1B**, in **CB**, unpaired electrons are not adjacent to other groups, which would limit the spatial spread of electron density from all directions. This diminishes the spatial confinement of the density distribution of these unpaired electrons. In the extended homologous systems with the same topological limits as **CB** with a maintained central cross-conjugated unit, the aromaticity of the specific parts of the molecule can determine the unpaired electron density distribution. For example, within the subset **L3O**, **L3H**, and **L3K**, the aromaticity of the peripheral rings shifts the unpaired electron density toward the center, pushing the unpaired electrons spatially closer compared to the reference compound **CB**, relatively loosening the topological restriction. This phenomenon of aromaticity shifting the unpaired electron density works in the opposite direction in Clar’s goblet: upon maximizing the number of aromatic Clar’s π-sextets (corresponding to the one of the most important RSs ***d-p*** in Figure 2b), unpaired electron density is shifted further from the center, diminishing the overlap between density distributions of spin-antiparallel unpaired electrons and thus tightening the topological restriction in **CG**. Within the series **L3O**, **L3H**, and **L3K**, the difference can be explained consistently with consideration of the size of the π-subspace in which unpaired electrons can delocalize and if the groups are electron-donating or electron-withdrawing. In **L3K**, the size of the π-space is larger due to carbonyl groups, which also shift the unpaired electron density away from the center. This diminishes the overlap between unpaired electron density distributions and, thus, tightens the topological restriction. The effect is opposite for the **L3O**, with electron-donating alkenoxy groups, which repel the unpaired electron density toward the center. This causes greater overlap between unpaired electron density distributions and thus loosens the restriction relative to compound **L3H**, which has a saturated carbon at that position without any strongly electron-donating or withdrawing groups.

In a recent study about Clar’s goblet, the authors stated that it has been experimentally shown that spins are partially localized and spatially segregated.^31^ This is fully consistent with the coarse-grained view of our analysis. However, one must always be aware of the possible loosening of the topological restriction due to (even minor) spatial overlap between delocalized unpaired electron density distributions. Thus, detailed insight into these distributions according to the structural peculiarities of the π-conjugated system allows tuning the tightness of the topological restriction more consistently. This allows rational design of compounds with more precisely controlled properties. Specifically, this is highly relevant for tuning the energy gaps between the spin states and can also serve as an auxiliary tool for engineering the ordering between different spin states in low-energy spectra. The topological analysis and consideration of the stabilization of specific π-system configurations by delocalization allow the rational design to have control over the spin spectrum in terms of state ordering, relations between different energy gaps, and the magnitude of these gaps. This means that the **TRAP** method allows for low-energy spectrum engineering for open-shell compounds. As a brief guideline, we can summarize the method used to design open-shell compounds from π-conjugated chemical structures as follows:

1. Define the nominal *N* minimum number of unpaired electrons according to resonance theory by tailoring the topology of the π-system.
2. Adjust the *tightness* of the topological restriction on the minimum number of unpaired electrons. Consider each possible pair of unpaired electrons that cannot make a *proper* π bond but can engage in long-range through-space on-bond pairing (e.g., *para* π bonds). One can design to obtain a tight topological restriction to have *K* number of unpaired electrons, and the remaining *M* unpaired electrons can have significant participation in long-range through-space on-bond pairings: *N* = *K* + *M*
3. If the open-shell character in a given substructure is to be increased without introducing the topological restriction, one should insert the (pro)­aromatic groups bridging the unpaired electrons. In the resulting structure, the configuration(s) with *N* + 2 unpaired electrons must have greater collective delocalization energy relative to configurations with *N* unpaired electrons by at least the resonance energy of 2–3 benzene rings to offset the energy of one fewer π bond.
4. The ground-state multiplicity is controlled by two factors: the number of unpaired electrons and the spin alignment between these unpaired electrons. In purely alternant systems, the GS multiplicity is determined by Ovchinnikov’s rule. In non-alternant systems, the spin alignment between the unpaired electrons should be determined by first estimating which atoms have the highest unpaired electron density and then determining the relative spin configurations between these spin centers based on the shortest through-bond paths.

Henceforth, as a result of our **TRAP** method, we are enabled to blueprint fully π-conjugated open-shell molecules from a multitude of bioorganic compounds with minimal modifications without having to rely on the presence of (pro)­aromatic parts in the system. These modifications should be made in a manner that does not introduce significant strain in the compound in order to avoid modification of the topology of the π-system and simultaneously allow synthetic accessibility.

### Neutral Porphyrin Polyradicals

Since one can connect at most 12 independent radicalogen groups to the π-system of porphine, there is a finite number of unique topologies that porphyrin polyradicals can have, accessed through a multitude of single-radical connection modifications of porphine. Nonetheless, the number of permutations can still be high, and it is helpful to analyze the limiting cases. The one obvious limit to analyze is at least how many unpaired electrons are topologically imposed when we connect 12 radicalogen groups to the conjugated π-system of porphine, expressed by the resonance structures of the compound **PF** in Figure 4a. This compound is nominally topologically restricted to have at least six unpaired electrons. Nevertheless, we must also note that this restriction is not necessarily complete. The loosening of this topological restriction can occur because the density distributions from different unpaired electrons may have significant spatial overlap. These unpaired electrons are not allowed to make on-bond pairing through the path of direct π-conjugation. However, there might be a significant contribution from the electronic configurations involving through-space π on-bond pairing as shown within the compound series in Figure 3 and with an orbital scheme for the porphyrin system in Figure 1b. Hence, although according to the topology of the π-system the compound would have been expected to have full tetraradical and hexaradical characters, this is not achieved because two pairs of spin-antiparallel (through-bond effect) unpaired electrons have their density distributions with sufficient overlap to lower the open-shell character within these subshells. This leaves two unpaired electrons within a tight topological restriction and the remaining four electrons within a loose topological restriction. This was verified by CASSCF­(10,10)/cc-pVDZ calculations, which show the frontier NO occupation numbers of 1.65, 1.64, 1.00, 1.00, 0.36, and 0.35 in the singlet ground state, implying full diradical character and significantly diminished tetraradical and hexaradical characters (*y*_1_ = 0.09 and *y*_2_ = 0.08, respectively). In addition to frontier NOs, we used localization to better express and analyze the spatial overlap of different unpaired electron density distributions (shown in different colors in Figure 4b) from six frontier CASSCF canonical orbitals. The significant reduction of tetraradical and hexaradical characters can be traced to the resonance structures and localized orbitals shown in Figure 4. Two sets of pairs of spin-antiparallel electrons in pyrrole rings have significant density on the carbon atoms that are spatially closer than the *para* atoms in benzene (see also Figure 1).

**Figure 4:**
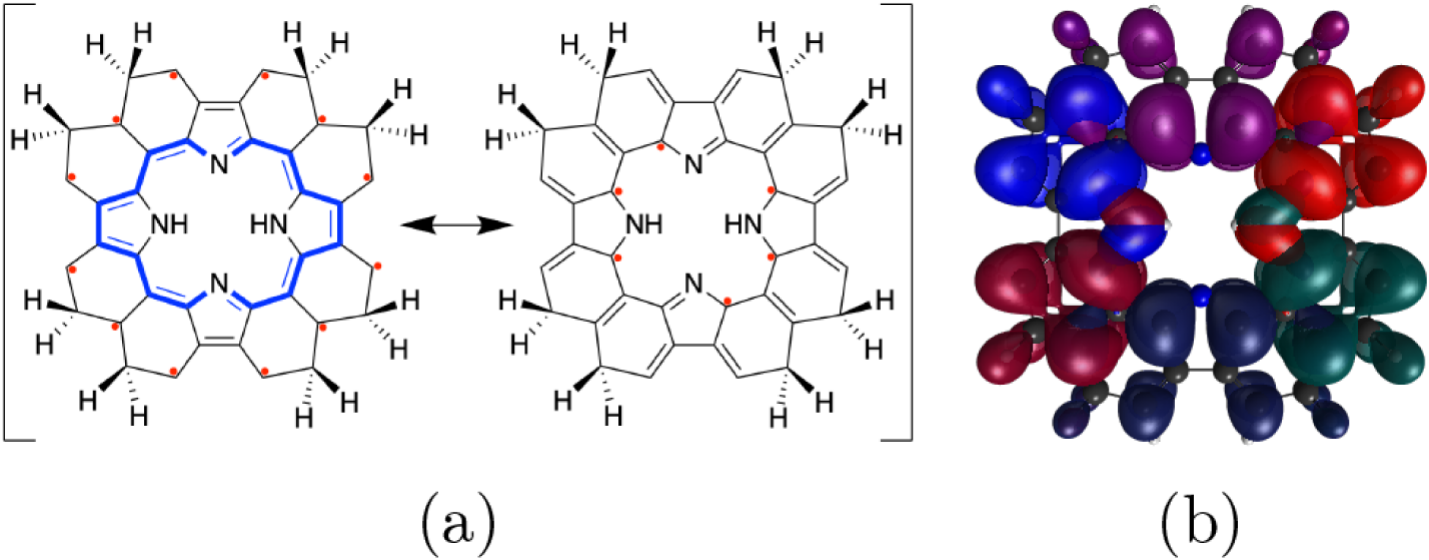
(a) The limiting resonance structures and (b) localized frontier CASSCF orbitals (in different colors) of the porphine derivative **PF** with 12 radicalogen groups.

The similar long-range π-bonding interaction in the five-membered ring-containing systems was also presented by Clar in his work.^32^ Apparently, the greater spatial overlap between topologically imposed unpaired electrons in the porphyrin systems can lower the open-shell character even more than in conjugated systems with only six-membered rings. This analysis shows that taking the naively trivial limit of connecting the maximum number of independent radicalogen groups to the porphine does not produce a tight topological restriction to have more than two unpaired electrons in the resulting porphyrin. Hence, one needs to analyze other instances if using fewer radicalogen groups is more effective in inducing a greater number of unpaired electrons in the ground state. In this work, we show a compound that surpasses this limit of topological restriction and has at least four fully unpaired electrons. In porphyrins, one needs to carefully analyze not only the topological restriction within the π-system but also the density distributions of unpaired electrons to allow sufficient delocalization to attenuate the possible through-space on-bond pairing interaction. One of the ways to achieve this in the ground state is to tailor the topology of the π-system such that it is consistent for these unpaired electrons to be spin-parallel according to the shortest through-bond π-conjugated path, while the other longer path is consistent with on-bond pairing, even though this pairing would leave other unpaired electrons, as is consistent with the imposed topological restriction. In non-alternant systems, this degree of freedom is present in the structure. Such a spin configuration prevents unpaired electrons from occupying the same regions of space, if we reason from the Pauli exclusion principle for Fermions. Hence, the loosening of the topological restriction can be attenuated with structural adjustments, maintaining the number of unpaired electrons and allowing the change of the spin configuration in the ground state. Moreover, the effect of tautomerism, based on which nitrogen atoms are bonded to hydrogen atoms, can have a significant influence on the open-shell character and is also analyzed.

To further exemplify the concept of tight and loose topological restrictions for porphyrins, we describe two tetrasubstituted systems. The first system has two isomers, **PA** and **PB**, which are related by the common tautomer **PT** and topological restriction on open-shell character is not imposed across tautomers, as shown in Figure 5a. The second system **PE** is restricted to have at least two unpaired electrons in both of its tautomers **PE-Z** and **PE-T** as shown in Figure 6a. Tetraradical RSs of **PA** and **PT** have 1 fewer π bond than diradical RSs and 2 fewer π bonds than closed-shell RSs, while for each, **PA** and **PT**, closed-shell, diradical, and tetraradical RSs have about the same total delocalization energy. Thus, we can predict that the open-shell character of these systems must be very low, as verified by the results in Figure 5b. Specifically, in **PA**, on-bond pairing between two pairs of unpaired electrons can happen without affecting the aromatic unit. In **PT**, a single on-bond pairing can happen without affecting the aromatic unit, but if on-bond pairings occur such that all electrons are paired, then the pyrrole ring in aromatic configuration can still be maintained. The difference between the aromatic resonance energy of the porphyrin 18 π-electron system and the pyrrole is not sufficient to offset the energy of even one π bond. **PB** and tautomers of **PE** shown in Figures 5a and 6a have the topological restriction on the lower bound of polyradical identity to diradical. Even though **PB** is nominally restricted to be at least a diradical, the connected radicalogen groups are on the adjacent atoms, and the density distributions of unpaired electrons end up spatially close. This leads to a nonzero overlap between π-type orbitals occupied by unpaired electrons and thus a non-negligible through-space on-bond pairing interaction, which loosens the topological restriction as shown in Figure 1b. For **PE**, the restriction is tight since radicalogen groups are not bonded to adjacent carbon atoms, and the resulting unpaired electron density distributions are more separated. Also, due to the aromaticity of the pyrrole rings, they are more excluded from the central region, where they could have had sufficient overlap so that the contribution of the configuration with through-space on-bond pairing would have been significant. Results of CASSCF spectra with annotated diradical and tetraradical characters in Figure 5 show that tautomers of **PE** have much higher diradical character than **PB** due to tighter topological restriction of lower-bound polyradical identity to diradical, and **PB** has much higher diradical character than **PA** and **PT** due to the complete absence of such topological restriction in the latter systems. The energy gap between the singlet GS and the first excited (triplet) state in **PA** is about 30–32 kcal/mol; in **PT**, it is about 31–34 kcal/mol, while in **PB**, it is about 9–11 kcal/mol. These gaps are even more diminished in tautomers of **PE**, in which GS is triplet (except for quintet UKS-optimized (QG) geometry of **PE-Z**, where the gap is inverted) and energy gaps are 0.95 kcal/mol for the TG of **PE-Z** and −0.47 kcal/mol for the QG of **PE-Z**. The triplet ground state is more pronounced in the **PE-T** tautomer with an energy gap of 4.1–4.4 kcal/mol. These results exemplify tuning energy gaps by adjusting the tightness of the topological restrictions.

**Figure 5:**
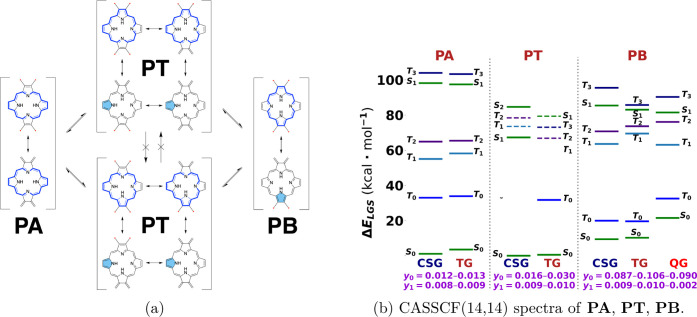
(a) Resonance structures of tautomerically connected porphyrins **PA**, **PT**, and **PB**; (b) CASSCF/cc-pVDZ spectra of each tautomer using RKS, UKS-triplet, and UKS-quintet optimized geometries (CSG, TG, and QG, respectively) with diradical (*y*_0_) and tetraradical (*y*_1_) characters.

**Figure 6:**
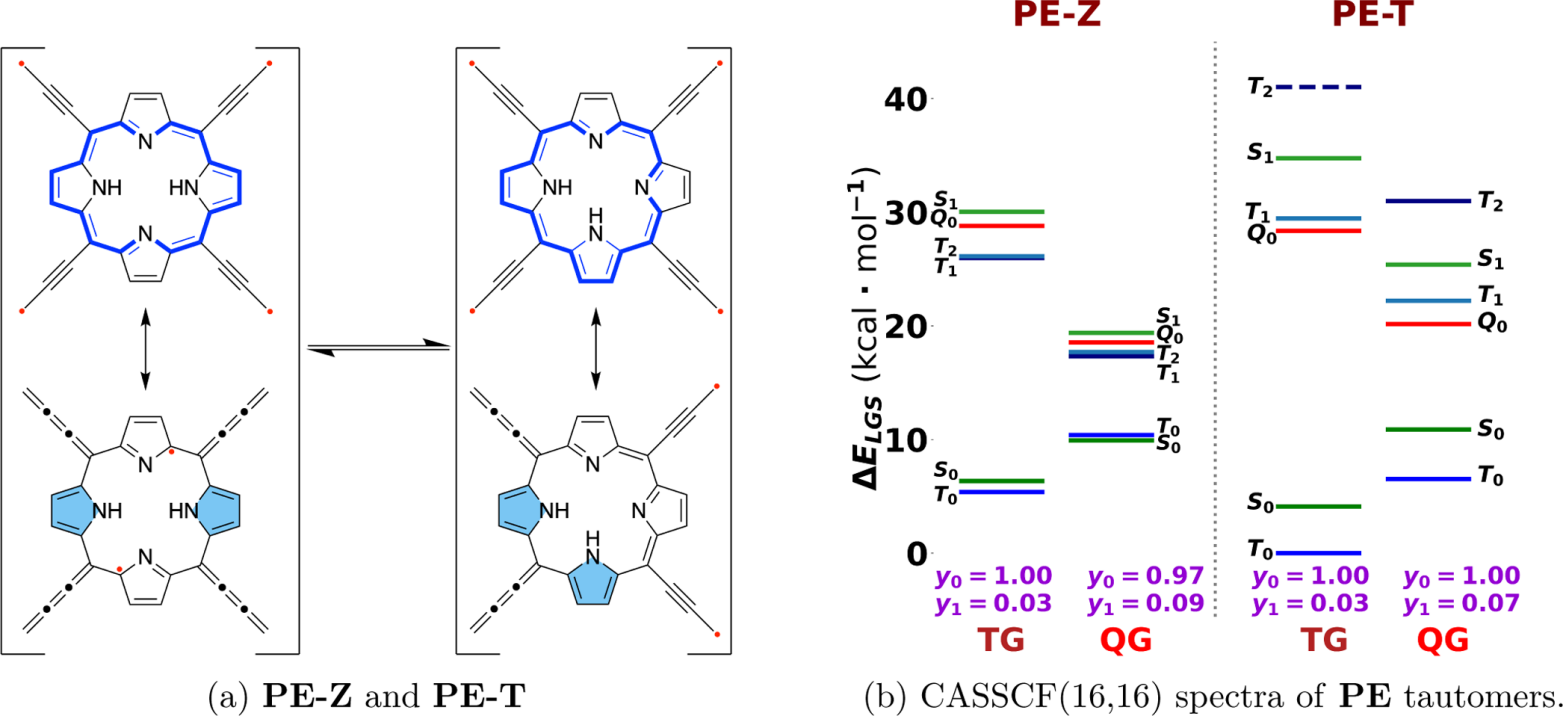
(a) Resonance structures of tautomers of porphyrin **PE** and (b) CASSCF­(16,16)/cc-pVDZ spectra of each tautomer using UKS-triplet and UKS-quintet geometries (TG and QG, respectively) with diradical (*y*_0_) and tetraradical (*y*_1_) characters.

The open-shell character for these compounds results from the specific topology of the π-system determined by the positions of the radicalogen groups connected to porphine, even though the local aromaticity of pyrrole groups and the global aromaticity of porphine alone are insufficient to induce an open-shell electronic structure. Thus, for a given porphyrin chemical species with insufficient (pro)­aromatic units to allow stabilization of open-shell configurations, the polyradical character can be persistent if all tautomeric forms are restricted to have a minimum number of unpaired electrons. Among **PA**, **PT**, and **PB**, the most stable tautomers are the closed-shell species **PA** and **PT**, with approximately the same stability, according to CASSCF calculations. Between the tautomers of **PE**, both of them among different geometries have the lowest-energy ground state (LGS) to be triplet, with the diradical triplet state of tautomer **PE-T** being the most stable according to the CASSCF calculations.

Moreover, we present the hexasubstituted porphyrin for which all tautomeric forms have at least four unpaired electrons due to topological restriction. The specific topology of the π-system gives rise to the quintet ground state as shown in Figure 7. The topological restriction in tautomers of **QD** is tight, and diradical and tetraradical characters approach 1 because the closest radicalogen groups are bonded to atoms in relative 1,3 positions in hexaradical RSs, while in some tetraradical RSs they are much further apart, as shown in Figure 7a. In tetraradical configurations, this leads to little spatial overlap, and the contributions of electronic configurations with through-space on-bond pairing are dramatically diminished compared to **PB** and **PF**. As is apparent from the frontier singly occupied natural orbitals for the tautomers of **QD** in Figure 7c to 7e, the unpaired electrons are fully delocalized in the molecule. This should be favorable for the chemical stability of **QD** if one also adds bulky groups to protect the peripheral atoms from radical reactions. Notably, one could also predict the ground-state multiplicity of these tautomers based on careful extension of Ovchinnikov’s rule following the proper through-bond path between radical centers as described in our previous works.^27,28^ The higher energy of the **QD-Y** tautomer can be explained based on the resonance structures in Figure 7a and unpaired electron density distribution from CASCI frontier NOs. In **QD-Y**, unpaired electrons are closer on average and have less space for delocalization, while in other tautomers, the π-system is used more uniformly among these unpaired electrons for delocalization.

**Figure 7:**
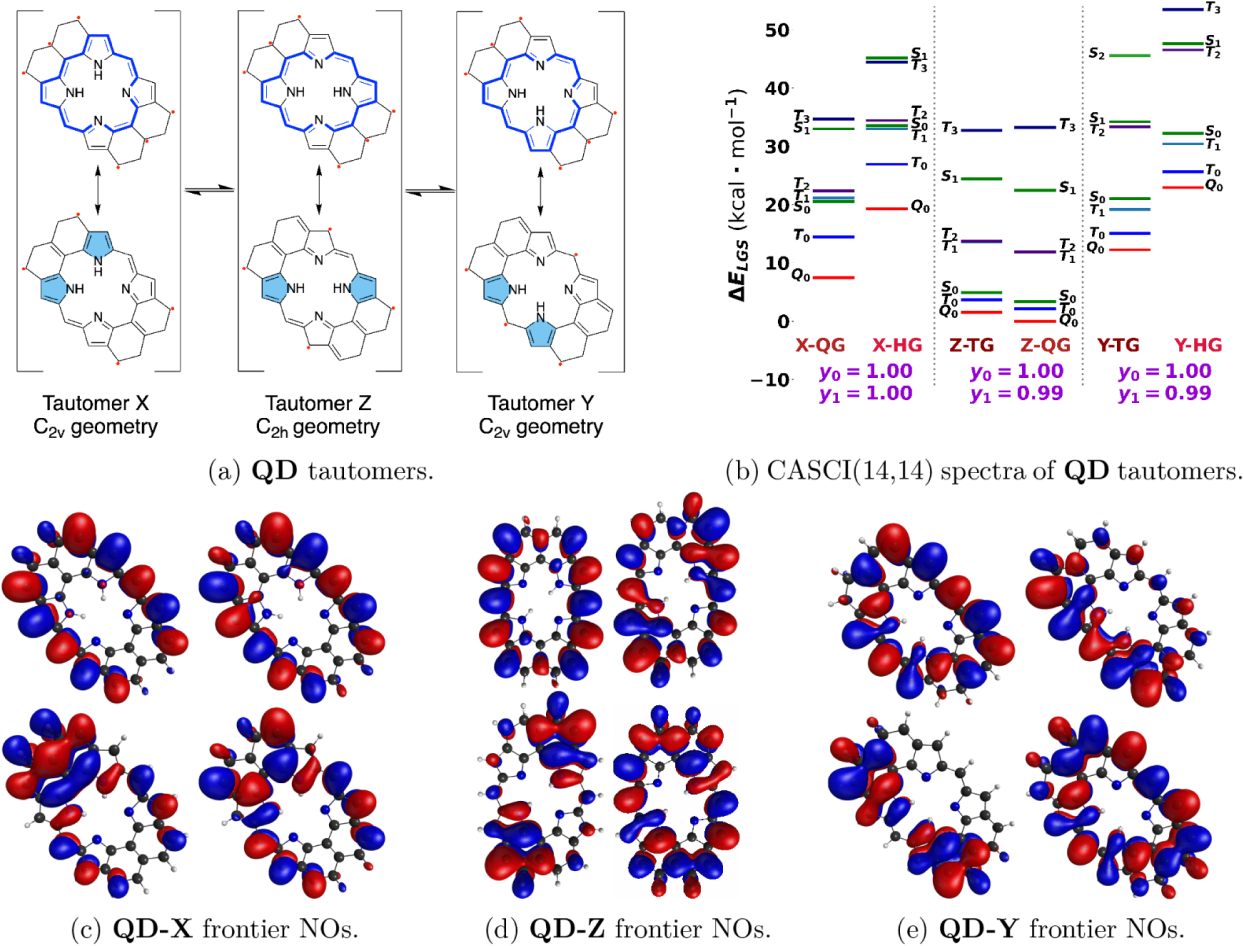
(a) Tautomer structures of **QD**, (b) CASCI spectra using UKS-triplet, UKS-quintet, and UKS-septet-optimized geometries (TG, QG, and HG, respectively) with diradical (*y*_0_) and tetraradical (*y*_1_) characters, and (c, d, and e) frontier singly occupied NOs of **QD-X**, **QD-Z**, and **QD-Y**.

### Charged and Metal-Inserted Porphyrin Polyradicals

We also examined the effects of single and double deprotonation of the system **QD**, with total resulting charges of −1 and −2, respectively. CASSCF calculations showed that the ground-state multiplicity might change upon transformation into the singly or doubly deprotonated (Figure 8) species. Nonetheless, the qualitative open-shell character is maintained with a similar unpaired electron density distribution (section S3 in the Supporting Information). We also studied the effect of the insertion of Mg^2+^ (common in biological porphyrins) such that the overall complex is neutral for doubly deprotonated **QD** and showed that the minimum number of unpaired electrons in the ground state is maintained. In addition, the spectral range of spin states diminishes compared to the metal-free **QD** as evident from Figure 9b. For example, the spectral range for the first six states in **QD-Z** (the most stable **QD** tautomer) is about 22.4 kcal/mol, while for **Mg-QD**, it is about 4.8 kcal/mol, compared using quintet UKS-optimized geometries. It is important to note that since the presented porphyrin systems are non-alternant, there are more degrees of freedom in the spin configuration of the ground state than in alternant systems. The singly deprotonated species has the same GS multiplicity as **QD**. However, as is apparent from the results for doubly deprotonated and magnesium-inserted species of tetraradical **QD**, there are some changes in the electronic structure that are noteworthy. In doubly deprotonated **QD** (dianion ${\mathrm{QD}}_{m2H}^{2-}$ ), the pyrrole rings in the center are more electron-rich and this repels unpaired electrons toward the peripheries of the π-subsystems, while these unpaired electrons are topologically forbidden from engaging in simultaneous on-bond pairings by through-bond π-conjugated paths. Nevertheless, density distributions from different unpaired electrons are pushed toward the same region of the structure as pyrrole rings push both unpaired electrons on each side toward one another. This increases the spatial overlap between unpaired electron density distributions, and through-space on-bond pairing interaction becomes a significant effect in the electronic structure.

**Figure 8:**
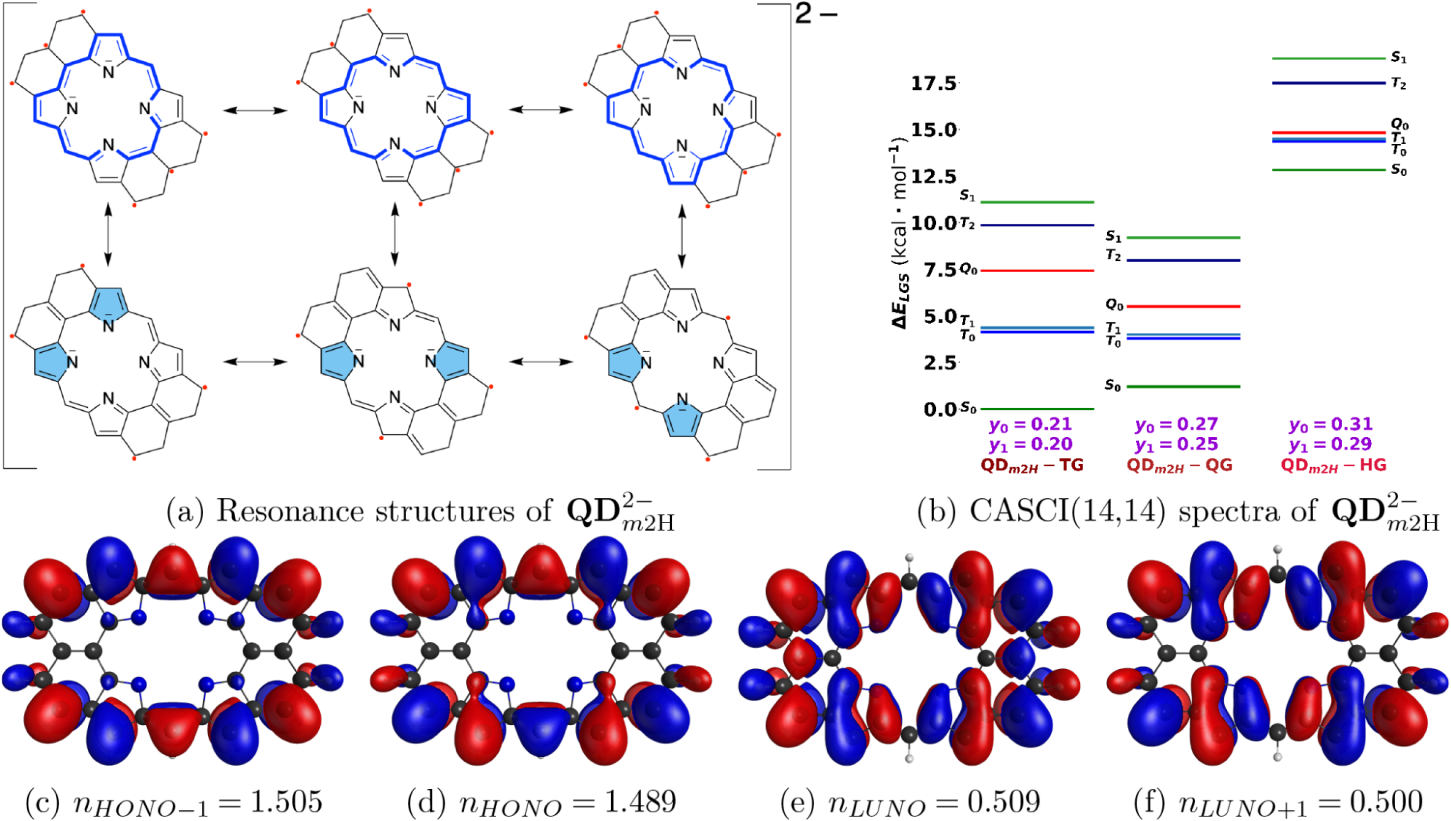
(a) Resonance structures of doubly deprotonated (with a charge of −2) **QD**. (b) CASCI spectra using UKS-triplet, UKS-quintet, and UKS-septet-optimized geometries (TG, QG, and HG, respectively) with diradical (*y*_0_) and tetraradical (*y*_1_) characters. (c-f) CASCI singlet ground state frontier NOs with occupation numbers, computed at quintet UKS-optimized geometry.

**Figure 9:**
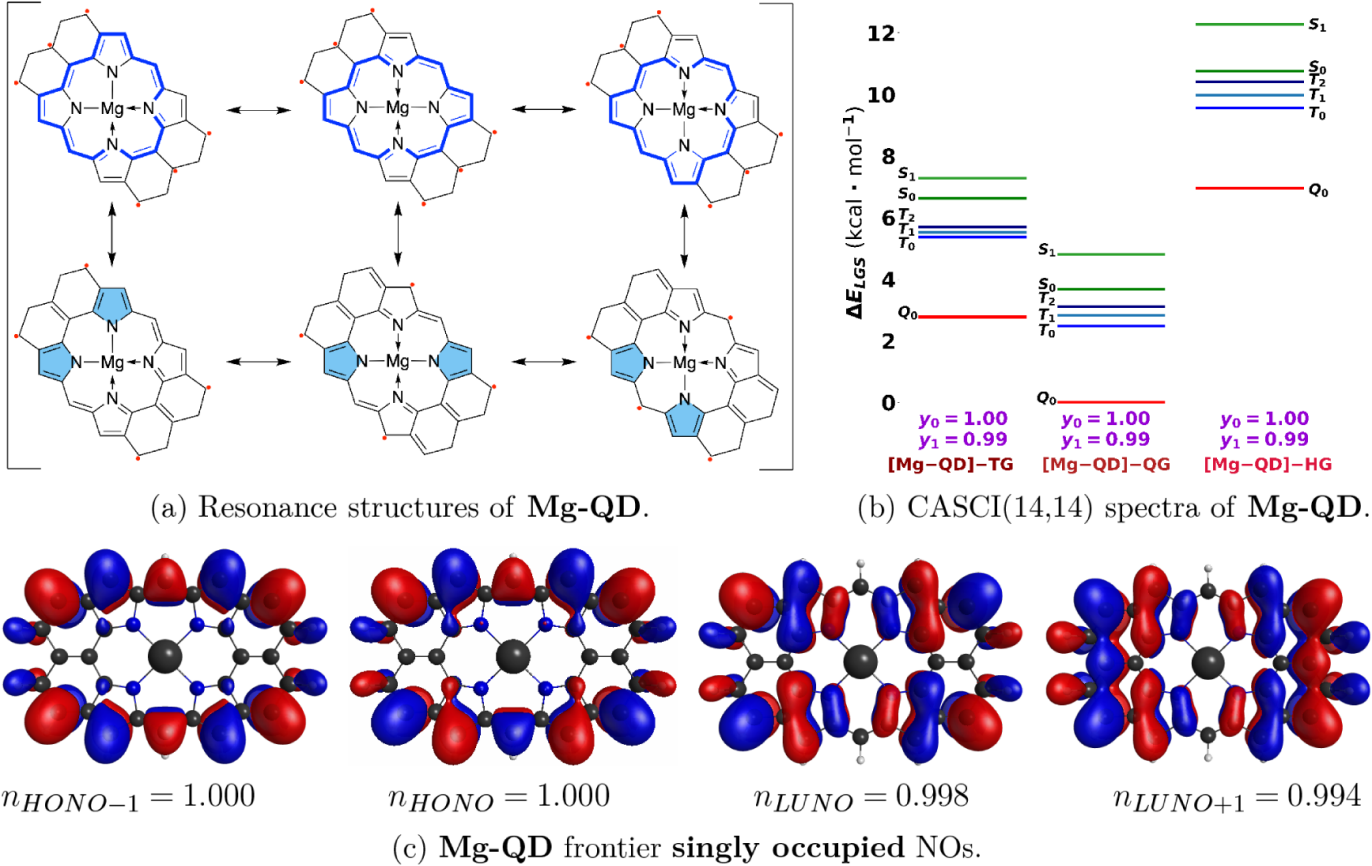
(a) Resonance structures of Mg^2+^ inserted in **QD**. (b) CASCI spectra of **Mg-QD** using UKS-triplet, UKS-quintet, and UKS-septet-optimized geometries (TG, QG, and HG, respectively) with diradical (*y*_0_) and tetraradical (*y*_1_) characters and (c) four singly occupied natural orbitals.

The effect is opposite in **Mg-QD** compared to dianion ${\mathrm{QD}}_{m2H}^{2-}$ . Insertion of the magnesium ion in the doubly deprotonated dianion recovers the electron density distribution similar to the **QD** but shifts more electron density toward the magnesium-filled porphyrin center, where through-space on-bond pairing is much weaker. Consequently, **QD** and **Mg-QD** both have full tetraradical characters, while the doubly deprotonated species ${\mathrm{QD}}_{m2H}^{2-}$ does not. Moreover, the insertion of a magnesium ion instead of two hydrogen atoms in free porphyrin **QD** stabilizes the quintet spin state relative to other spin states more than in **QD**. Notably, the spectral range is dramatically diminished in **Mg-QD**, but the gap between the quintet GS and the triplet first excited state is 2.1 kcal/mol in **QD-Z**, while it is 2.5 kcal/mol in **Mg-QD**, compared using quintet UKS-optimized geometries. Since in **Mg-QD** unpaired electrons are more pushed toward the center than in ${\mathrm{QD}}_{m2H}^{2-}$ , the *S* = 2 spin configuration is even more stabilized compared to other spin configurations, because the spin-parallel electron configuration minimizes electron–electron repulsion and is simultaneously consistent with the topology of the π-system. In contrast, in the case when *S* = 0, upon through-space on-bond pairing, unpaired electrons have to be pushed in spatial proximity, increasing the repulsion, without the possibility of on-bond pairing that creates the proper π bond. Also, as unpaired electrons are more evenly spread in **Mg-QD** than in ${\mathrm{QD}}_{m2H}^{2-}$ dianion, spin–spin coupling strengths are relatively diminished. We can also extend the described principles to the transition metal ions, which can insert into the core of the open-shell porphyrin compound. Hence, if the stable complex between the metal ion with nonzero unpaired electrons is established, this allows us to design purely organic open-shell porphyrins and then insert the magnetic metal ion to create a second magnetic subsystem. The interaction between these subsystems leads to a more complex spin spectrum due to a higher amount of unpaired electrons in the open-shell π-subspace.

## Conclusions

In conclusion, using our recently formulated **TRAP** method/theory, we described the rational design of fully π-conjugated, highly delocalized, and high-spin porphyrin polyradicals with full diradical and tetraradical characters. We also showed that such a topologically rational approach is successful in providing optimal designs in terms of achieving a higher number of unpaired electrons in the ground or low-energy electronic states in the resulting porphyrin system than naively expected by connecting the maximum possible number of radicalogen groups to the porphine. This shows that the topologically rational design method **TRAP** can be used to find nontrivial polyradical structures. The **TRAP** method can be applied to many classes of synthetically accessible and common organic π-conjugated compounds to design open-shell molecules with desirable properties applicable in organic electronics, spintronics, single-molecule devices, biosensors, quantum technologies, etc. The proposed metal-free open-shell porphyrins exemplify designing organic compounds with magnetic ordering without having to use metals as magnetic centers but can have persistent open-shell character even if a metal ion is inserted. The inclusion of magnetic or nonmagnetic metals in organic open-shell porphyrins can allow for the controlled alteration of the spin spectrum. This can be used to reversibly tune the spin state excitation energies as well as create doubly magnetic metal–organic systems. These new possibilities are certainly pertinent to study and are being further explored by our group.

## Methods and Computational Details

For geometry optimizations of the molecules, Density Functional Theory (DFT) was used with Gaussian 2016.^35^ We used geometries optimized for the state with appropriate spin multiplicity by performing restricted and unrestricted Kohn–Sham (RKS and UKS) DFT calculations, with Generalized Gradient Approximation (GGA) exchange-correlation functional BLYP^36,37^ and Dunning’s correlation-consistent triple-ζ basis set cc-pVTZ.^38^ To verify that the optimized geometry corresponded to the minimum on the Potential Energy Surface (PES), a Hessian with respect to nuclear coordinates was computed and checked.

To study the multiconfigurational wave function of the presented polyradical­(oid)­s, the Complete Active Space Self-Consistent Field (CASSCF) method was used.^39–45^ The initial guess orbitals for CASSCF calculations were UHF natural orbitals, as they are one of the best starting orbitals for CASSCF.^46–48^ For a localized representation of frontier CASSCF canonical orbitals, we used Pipek–Mezey^49^ localization method available in the General Atomic and Molecular Electronic Structure System (GAMESS).^50,51^

In addition, when the CASSCF calculations are not affordable or have convergence problems, we use Complete Active Space Configuration Interaction (CASCI) calculations to characterize the low-energy spectrum of spin states of polyradicals. The initial guess can be UHF natural orbitals of the state with the highest-allowed multiplicity for a given polyradical, which reproduces the electron density of multiple states because of the mixing of pure states in UHF. This method is called Unrestricted Natural Orbitals Complete Active Space CI (UNO-CAS), which was shown to reproduce CASSCF results of similar active space sizes qualitatively by determining electronic structures and energies along the PES of different molecules. In most of the PES regions, the UNO-CAS energy curve was essentially parallel to the CASSCF energy curve.^47^ All electronic structure calculations were performed in GAMESS. Since we have to rely on the DFT-optimized geometries, we optimized the geometry of each compound for the spin states that are topologically allowed in the low-energy spectrum. This represents the set of points on the PES that are sampled by using the DFT-optimal geometries for each spin state. Then, by computing the low-energy spectrum with the CASSCF method on each such geometry and comparing changes in energies of spin states, we can approximately deduce the structure of the low-energy spectrum if CASSCF-optimized geometries were used. In the comparison of the open-shell character of the topologically restricted diradical series shown in Figure 3, we tabulate the occupation numbers of the frontier natural orbitals which correspond to the ground state determined with the DFT-optimized geometry, which results in the lower CASSCF electronic energy.

For calculations of open-shell character indices, Yamaguchi’s approach was employed,^52^ based on occupation numbers (*n*_NO_) of frontier natural orbitals. The *n*-radical character *y*_*n*_ varies from *y*_*n*_ = 0, meaning no *n*-radical character, to *y*_*n*_ = 1, meaning full *n*-radical character. The highest occupied natural orbital (HONO) is defined as the orbital that has the lowest *n*_NO_ among NOs with *n*_NO_ ≥ 1. The lowest unoccupied natural orbital (LUNO) is defined as the orbital that has the highest *n*_NO_ with *n*_NO_ ≤ 1. In UHF, *n*_HONO–*i*_ + *n*_LUNO+*i*_ = 2.000, also usually the case for CASSCF and CASCI NOs. 2­(*i* + 1)-ple radical character is calculated as follows:

$$T_{i}=\frac{n_{\mathrm{HONO}-i}-n_{\mathrm{LUNO}+i}}{2}\to y_{i}=1-\frac{2T_{i}}{1+{T_{i}}^{2}}$$

By substituting *i* = 0, we obtain *y*_0_ and 2­(0 + 1)-ple radical character. Hence, *y*_0_ is a diradical character index, and when *i* = 1, then *y*_1_ is a tetraradical character index and so on for higher-order polyradical character indices.

## Supplementary Material



## Data Availability

The data underlying this study are available in the published article and its Supporting Information.

## Notes

Computational details of geometry optimization, computational details of CASSCF and CASCI calculations, CASSCF/CASCI results with relevant frontier natural orbitals for every compound, and optimized geometries for every relevant state of all the compounds presented in this study (PDF)

## Notes

We dedicate this article to the memory of Prof. Santiago Olivella Nello (1944–2025).
