# Supplementary material for: High-Spin Porphyrin Polyradicals
Source: ACS Omega. 2026 Jan 5;11(2):3368–79. doi: 10.1021/acsomega.5c10439 (PMC12824932; doi:10.1021/acsomega.5c10439)
Supplement: Supplementary file 1 [file ao5c10439_si_001.pdf]

Supporting Information

High-Spin Porphyrin Polyradicals

Sergi Betkhoshvili,[†] Jordi Poater,[*,†,¶], Ibério de P. R. Moreira[*,‡] and Josep Maria Bofill[*,†]

†*Departament de Química Inorgànica i Orgànica and Institut de Química Tèorica i Computacional (IQTCUB), Universitat de Barcelona, Martí i Franquès 1–11, 08028 Barcelona, Spain.* E-mail: jmbofill@ub.edu

‡*Departament de Ciència de Materials i Química Física and Institut de Química Tèorica i Computacional (IQTCUB), Universitat de Barcelona, Martí i Franquès 1–11, 08028 Barcelona, Spain.* E-mail: i.moreira@ub.edu

¶*ICREA, Pg. Lluís Companys 23, 08010 Barcelona, Spain.* E-mail: jordi.poater@ub.edu

Contents

S1. Topology of the Conjugated System of Porphyrin **S1**

S2. Methods and Computational Details **S2**
S2.1. Geometry Optimization S2
S2.2. CASSCF and CASCI Calculation Details S2

S3. Calculated Results **S3**

S4. Optimized Geometries **S10**

S1. Topology of the Conjugated System of Porphyrin

The porphine has 18 π-electrons in its globally aromatic system and contains two pyrrole rings, which could each assume a 6 π-electron configuration. However, both pyrrole rings cannot have a 6 π-electron aromatic configuration at the same time when the porphine system is in an 18 π-electron globally aromatic configuration. Depending on the number and position of the radical substituents in the porphine, different open-shell behavior can be induced so that, in addition to aromatic stabilization, the topological restriction is imposed on the lower-bound number of unpaired electrons in the obtained π-system. Hence, based on these topological principles, we can design open-shell compounds that have a nonzero minimum number of unpaired electrons even if there is no source of sufficient delocalization energy stabilization from the groups that would bridge these radical centers to offset the energy of fewer π bonds in higher-order polyradical configurations compared to lower-order polyradical configurations.

Furthermore, one can design such a topology of a π-system in porphyrin that restricts the lower-bound number of unpaired electrons, is not globally aromatic, but has two pyrrole rings in aromatic configuration. This means we can induce the open-shell character by the topological restriction when the aromatic resonance stabilization would not be sufficient to offset the energy of the broken π bond that led to the open shell. However, when this necessity is removed, the aromatic resonance stabilization can increase the contribution of the structure with the nominal lower-bound number of unpaired electrons and thus allows for the further refinement in the systematic control of the number of unpaired electrons in the system. Examples of such systems are **PE-Z** and **QD**, which have both of their pyrrole rings in aromatic configuration when the number of unpaired electrons is the same as the nominal lower-bound number of unpaired electrons imposed by topological restriction. In such a case, the topological restriction becomes tighter and aromaticity further reinforces/stabilizes the open-shell electronic structures. This effect from the aromaticity also affects the density distribution of the unpaired electrons within the molecule, as they are more excluded from the aromatic subsystems than the rest of the accessible π-system.

In addition, one can distinguish between *tight* and *loose* topological restrictions in the compound. Even though **PB** is topologically restricted to be at least a diradical, the restriction is loosened due to the spatial proximity and nonzero overlap (thus, through-space interaction) of the orbitals that bear these unpaired electrons. The schematic representation of this is given in Figure S2a.

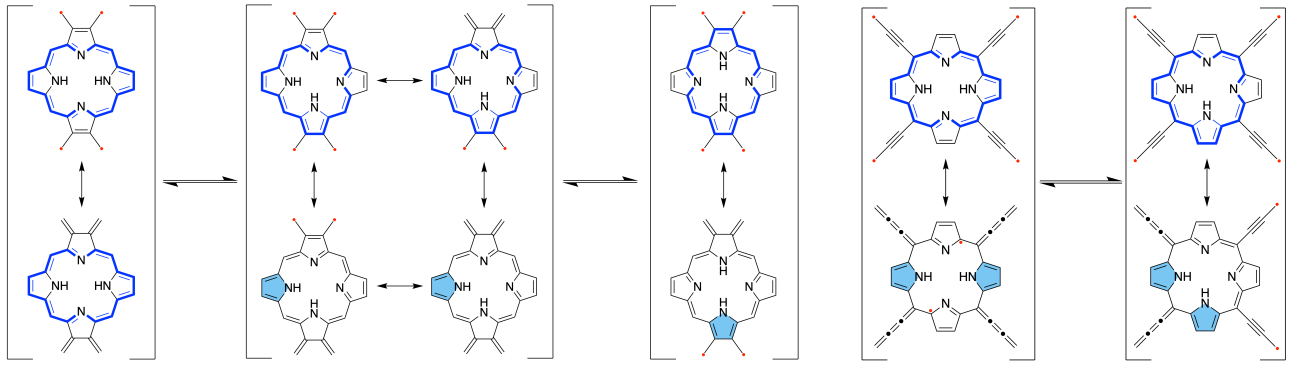

(a) **PA**,**PT**, **PB** **(b)** **PE-Z** and **PE-T**

Figure S1. Resonance structures of the tautomers of presented tetrasubstituted porphyrins.

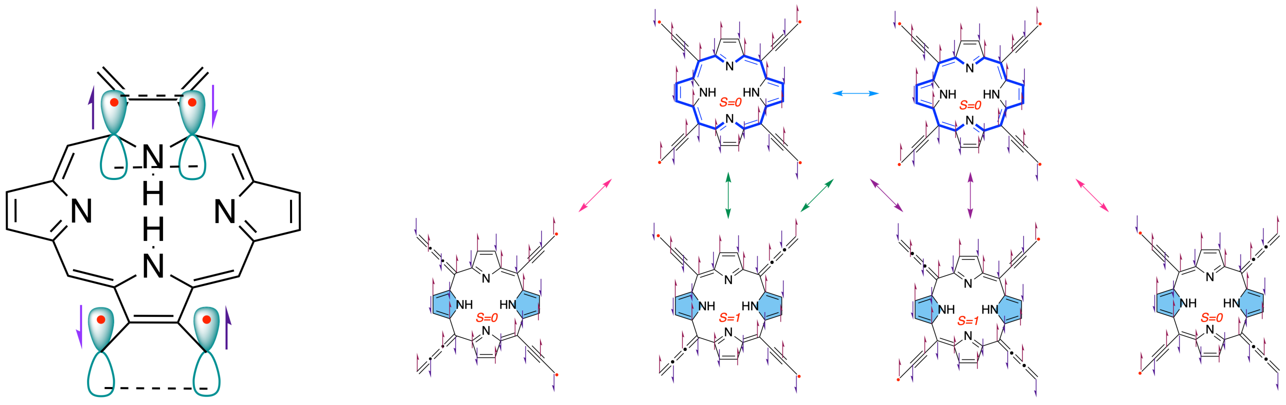

(a) **(b)** **PB**: colored arrows show if the given structures are interconvertible by continuous conjugation chain.

Figure S2. (a) Illustration of loosening of the topological restriction on the minimum number of unpaired electrons by long-range through-space on-bond pairing between unpaired electrons. (b) Analysis of the preferred spin configuration of **PE-Z** using representative resonance structures.

S2. Methods and Computational Details

S2.1. Geometry Optimization

For geometry optimizations of the molecules, Density Functional Theory (DFT) was used with Gaussian 2016.[1] We used geometries optimized for the state with appropriate spin multiplicity by performing restricted and unrestricted Kohn-Sham (RKS and UKS) DFT calculations using Generalized Gradient Approximation (GGA) exchange-correlation functional BLYP[2,3] without any relativistic corrections. The Dunning's correlation-consistent triple-ζ basis set cc-pVTZ[4] was used for geometry optimizations. In order to verify that the optimized geometry corresponded to the minimum on the Potential Energy Surface (PES), a Hessian with respect to nuclear coordinates was computed which was verified to have all positive eigenvalues, translating into all real calculated infrared absorption frequencies corresponding to the determined normal modes. We remark that all computations were carried out for a single, isolated molecule in the gas phase.

S2.2. CASSCF and CASCI Calculation Details

Hartree-Fock method with its restricted and unrestricted formalism (RHF and UHF, respectively) is a computationally cheap tool to evaluate whether or not the molecule has an open-shell electronic structure. To achieve this, we need to compute RHF and UHF solutions separately and compare them. If the UHF solution has lower energy than the RHF solution, it means that the closed-shell electronic wave function is unstable and the ground-state wave function is stable with an open-shell, UHF solution. For Hartree-Fock calculations, we used the General Atomic and Molecular Electronic Structure System (GAMESS).[5,6] The basis set used for all calculations except for geometry optimizations was Dunning's correlation-consistent double-ζ basis set cc-pVDZ.[4] To study the multiconfigurational wave function of polyradical(oid)s presented in this work, a multireference method such as Complete Active Space Self-Consistent Field (CASSCF), which is also known as Full Optimized Reaction Space (FORS), was used.[7–13] CASSCF calculations were performed using GAMESS. The initial guess orbitals for

CASSCF calculations were UHF natural orbitals as they are one of the best starting orbitals for CASSCF.[14–16] Natural orbitals (NOs) are eigenvectors of the first-order density matrix operator, and their corresponding eigenvalues are natural orbital occupation numbers (n_{NO}).[17] The largest affordable calculation for CASSCF with our resources is 16 electrons in 16 orbitals, i.e. CASSCF(16,16). We used the natural orbitals occupation number criteria around the frontier orbitals and selected active space with consideration of the electronic structure. The orbitals which were included are usually somewhat separated from the rest of the π-system, so that the frontier π-subspace can be described with high qualitative accuracy. In addition, when the CASSCF calculations are not affordable or have convergence problems, we use Complete Active Space Configuration Interaction (CASCI) calculations to characterize the low-energy spectrum of spin states of polyradicals. The well-established choice is using UHF natural orbitals (of the state with highest-allowed multiplicity for a given polyradical), which reproduces the electron density of multiple states because of the mixing of pure states in UHF. This method is called unrestricted natural orbitals complete active space CI, UNO-CAS, which has been shown to reproduce CASSCF results of similar active space sizes very well qualitatively. This was shown with determining electronic structures and energies along potential energy surfaces (PES) of different molecules and it was shown in most of the PES region that the UNO-CAS energy curve is essentially parallel to the CASSCF energy curve.[15]

For calculations of polyradical character indices, Yamaguchi's approach was employed,[18] based on occupation numbers (n_{NO}) of natural orbitals. The n-radical character y_n varies from $y_n = 0$ meaning no n-radical character to $y_n = 1$ meaning full n-radical character. The highest occupied natural orbital (HONO) is defined as the orbital that has the lowest n_{NO} among natural orbitals (NOs) with $n_{NO} \geq 1$. The lowest unoccupied natural orbital (LUNO) is defined as the orbital which has the highest n_{NO} among NOs with $n_{NO} \leq 1$. In UHF, $n_{HONO-i} + n_{LUNO+i} =$ 2.000, which is usually also manifested in CASSCF NOs. $2(i+1)$-ple radical character is calculated as follows:

$$T_i = \frac{n_{HONO-i} - n_{LUNO+i}}{2} \rightarrow y_i = 1 - \frac{2T_i}{1 + {T_i}^2}$$

By substituting $i = 0$, we obtain y_0, and $2(0+1)$-ple radical character. Hence, y_0 is a diradical character index. Analogically, by substituting $i = 1$, we obtain y_1, which is a tetraradical character index and similarly for higher-order polyradical character indices.

S3. Calculated Results

The results of CASSCF calculations for the tetrasubstituted porphine **PA** are given in Tables S1 and S2, for RKS-DFT and triplet UKS-DFT optimized geometries, respectively, with orbital symbolic assignations according to Figure S3.

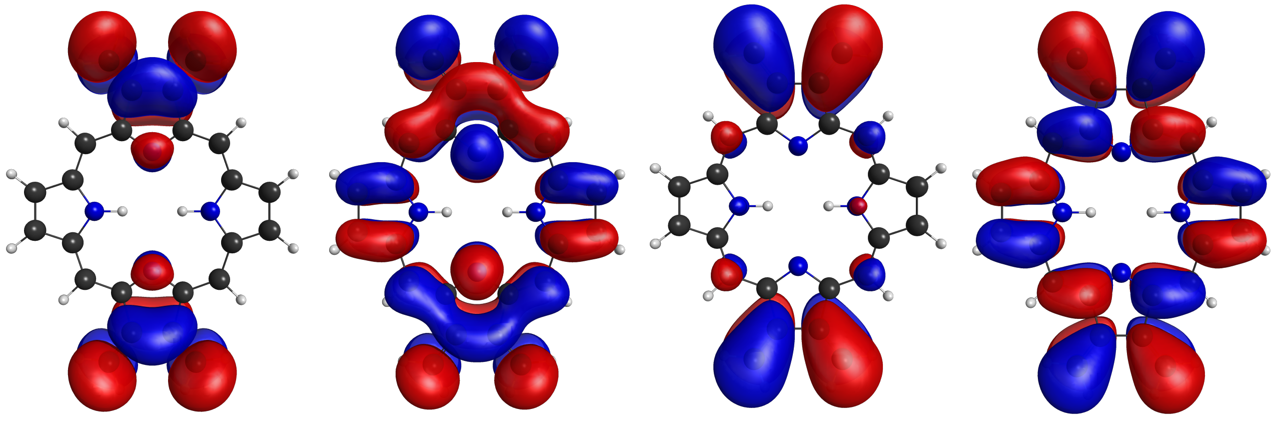

(a) C_0 **(b)** C_1 **(c)** C_1' **(d)** C_2

Figure S3. CASSCF frontier natural orbitals of **PA** with symbolic assignations. Isosurfaces with the value of 0.015.

As evident from the occupation numbers and energy gaps, the open-shell character of the **PA** is very small. This can be explained from Figure S1a as the configurations with zero, two and four unpaired electrons still possess the globally aromatic 18 π-electron system. Hence, there is no appreciable aromatic resonance energy of diradical and tetraradical configurations relative to the closed-shell configurations to offset the energy of the broken π bond(s) that would lead to diradical (or tetraradical) electronic structure. A similar observation can be made for the **PT** connecting tautomer of **PA** and **PB**, which does not have any restriction or sufficient resonance stabilization to assume significant open-shell character.

Table S1. CASSCF(14,14)/cc-pVDZ states of **PA** determined by state-specific calculations using RKS/DFT-optimized geomery. For all the calculations, UHF quintet NOs were used as initial guess. Under HONO, LUNO, etc. three columns represent symmetry of the particular orbital, its symbolic representation according to Figure S3 and NO occupation number.

State	Symmetry	Energy (a.u.)	CASSCF NOs symmetry, identity and occupation number												ΔE from GS (cm^{-1})
			HONO − 1			HONO			LUNO			LUNO + 1			
S_0	A_g	-1137.2273937606	B_{2g}	$C_1^{'}$	1.882	A_u	C_2	1.852	B_{1g}	C_1	0.137	B_{3u}	C_0	0.126	0
T_0	B_{1u}	-1137.1765367859	B_{2g}	$C_1^{'}$	1.881	A_u	C_2	1.002	B_{1g}	C_1	0.996	B_{3u}	C_0	0.122	11162
T_1	B_{2u}	-1137.1414461223	B_{2g}	$C_1^{'}$	1.888	A_u	C_2	1.005	B_{2g}	C_{1-2} [a]	0.985	B_{1g}	C_1	0.122	18863
T_2	B_{3g}	-1137.1257804176	B_{2g}	$C_1^{'}$	1.575	A_u	C_2	1.329	B_{3u}	C_0	0.642	B_{1g}	C_1	0.447	22302
S_1	A_g	-1137.0726989148	B_{2g}	$C_1^{'}$	1.652	B_{1g}	C_1	1.351	A_u	C_2	0.600	B_{3u}	C_0	0.368	33952
T_3	A_g	-1137.0634974578	B_{2g}	$C_1^{'}$	1.530	B_{1g}	C_1	1.008	A_u	C_2	0.846	B_{3u}	C_0	0.534	35971

Table S2. CASSCF(14,14)/cc-pVDZ states of **PA** determined by state-specific calculations using triplet UKS/DFT-optimized geomery. For all the calculations, UHF quintet NOs were used as initial guess. Under HONO, LUNO, etc. three columns represent symmetry of the particular orbital, its symbolic representation according to Figure S3 and NO occupation number.

State	Symmetry	Energy (a.u.)	CASSCF NOs symmetry, identity and occupation number												ΔE from GS (cm^{-1})
			HONO − 1			HONO			LUNO			LUNO + 1			
S_0	A_g	-1137.2235522914	B_{2g}	$C_1^{'}$	1.879	A_u	C_2	1.847	B_{1g}	C_1	0.142	B_{3u}	C_0	0.130	0
T_0	B_{1u}	-1137.1751335779	B_{2g}	$C_1^{'}$	1.878	A_u	C_2	1.002	B_{1g}	C_1	0.996	B_{3u}	C_0	0.125	10627
T_1	B_{2u}	-1137.1364465160	B_{2g}	$C_1^{'}$	1.886	A_u	C_2	1.006	B_{2g}	C_{1-2} [a]	0.983	B_{1g}	C_1	0.141	19118
T_2	B_{3g}	-1137.1249702080	B_{2g}	$C_1^{'}$	1.570	A_u	C_2	1.329	B_{3u}	C_0	0.642	B_{1g}	C_1	0.453	21636
S_1	A_g	-1137.0739016592	B_{2g}	$C_1^{'}$	1.643	B_{1g}	C_1	1.342	A_u	C_2	0.608	B_{3u}	C_0	0.378	32845
T_3	A_g	-1137.0645762542	B_{2g}	$C_1^{'}$	1.526	B_{1g}	C_1	1.013	A_u	C_2	0.845	B_{3u}	C_0	0.534	34891

[a] C_{1-2} means the orbital which has attributes from denoted orbital C_1 and C_2 but is neither of them precisely.

In comparison, the energy gaps are smaller and the open-shell character is higher in **PB** due to the presence of a topological restriction to have two unpaired electrons. However, this restriction is loose due to the spatial proximity of the radical centers, which leads to appreciable contribution from the electronic configurations with long-range through-space on-bond pairing as illustrated in Figure S2a. This leads to open-shell character diminishing from pure diradical, but is nonetheless higher than in **PA** and **PT** due to the complete absence of topological restriction in the latter compounds.

Table S3. CASSCF(14,14)/cc-pVDZ states of **PT** determined by state-specific calculations using RKS/DFT-optimized geomery. For all the calculations, UHF quintet NOs were used as initial guess. Under HONO, LUNO, etc. three columns represent symmetry of the particular orbital, its arbitrary symbolic representation[b] and NO occupation number.

State	Symmetry	Energy (a.u.)	CASSCF NOs symmetry, identity and occupation number												ΔE from GS (cm^{-1})
			HONO − 1			HONO			LUNO			LUNO + 1			
S_0	$A^{'}$	-1137.2291429097	$A^{''}$	D_1	1.874	$A^{''}$	D_2	1.838	$A^{''}$	D_3	0.168	$A^{''}$	D_4	0.131	0.00
T_0	$A^{'}$	-1137.1752208524	$A^{''}$	D_1	1.876	$A^{''}$	D_2	1.014	$A^{''}$	D_3	0.991	$A^{''}$	D_4	0.130	11835
S_1	$A^{'}$	-1137.1219755904	$A^{''}$	D_1	1.577	$A^{''}$	D_2	1.229	$A^{''}$	D_3	0.88	$A^{''}$	D_4	0.345	23521
T_1	$A^{'}$	N/A													
T_2	$A^{'}$	N/A													
S_2	$A^{'}$	-1137.0942380564	$A^{''}$	D_1	1.619	$A^{''}$	D_2	1.041	$A^{''}$	D_3	0.900	$A^{''}$	D_4	0.475	29608

Table S4. CASSCF(14,14)/cc-pVDZ states of **PT** determined by state-specific calculations using triplet UKS/DFT-optimized geomery. For all the calculations, UHF quintet NOs were used as initial guess. Under HONO, LUNO, etc. three columns represent symmetry of the particular orbital, its arbitrary symbolic representation[b] and NO occupation number.

State	Symmetry	Energy (a.u.)	CASSCF NOs symmetry, identity and occupation number												ΔE from GS (cm^{-1})
			HONO − 1			HONO			LUNO			LUNO + 1			
S_0	$A^{'}$	-1137.2282796486	$A^{''}$	D_1	1.871	$A^{''}$	D_2	1.780	$A^{''}$	D_2	0.220	$A^{''}$	D_3	0.135	0
T_0	$A^{'}$	-1137.1785470320	$A^{''}$	D_1	1.870	$A^{''}$	D_2	1.011	$A^{''}$	D_2	0.992	$A^{''}$	D_3	0.132	10915
T_1	$A^{'}$	-1137.1327048060	$A^{''}$	D_1	1.644	$A^{''}$	D_2	1.208	$A^{''}$	D_2	0.822	$A^{''}$	D_3	0.349	20976
T_2	$A^{'}$	N/A													
T_3	$A^{'}$	N/A													
S_1	$A^{'}$	N/A													

[b] The shapes of tabulated frontier orbitals are not as regular across different states as in the other presented compounds. Thus, the symbolic representations are denoted as distorted orbitals (D) with arbitrary numbering.

In order to increase the diradical character to essentially unity, we need a tight topological restriction to impose at least two unpaired electrons in the system. This is achieved by tailoring the topology of π-conjugation in the

tetrasubstituted porphine **PE-Z**, which has radical substituents quite distanced from one another and also the diradical resonance structure (RS) has both pyrrole rings in aromatic configuration. This reinforces the topological constraint and the electronic structure with two fully unpaired electrons is achieved. Furthermore, even though there exists a through-bond path such that on-bond pairing between unpaired electrons is possible, such pairing would leave other unpaired electrons behind. Moreover, there is another path of π-conjugation which causes radical centers to be ferromagnetically coupled. The analysis can be extracted from representative resonance structures in Figure S2b by comparing them to CASSCF frontier natural orbitals and occupation numbers. The unpaired electron density distribution in the ground state is mainly in orbitals C_1 and C_2 according to the symbolic assignations in Figure S5. Such an electron density distribution indicates that the closure of the shell across pyrrole rings that would diminish collective aromatic resonance stabilization is not favored. Hence, the more appropriate representative RSs towards the resonance hybrid are the RSs with two unpaired electrons and $S = 1$ equally contributing to the resonance hybrid due to symmetry. This is consistent with full delocalization of unpaired electrons across non-aromatic rings, while being more excluded from aromatic pyrrole rings. Moreover, it is noteworthy that there is always very little unpaired electron density on all nitrogen atoms in the two CASSCF frontier orbitals. This could suggest that through-bond coupling, which controls the spin alignment between radical centers, does not pass through nitrogen and is mainly all-carbon path. This is consistent with the results of the CASSCF calculations and the analysis of the spin alignment in different RSs in Figure S2b.

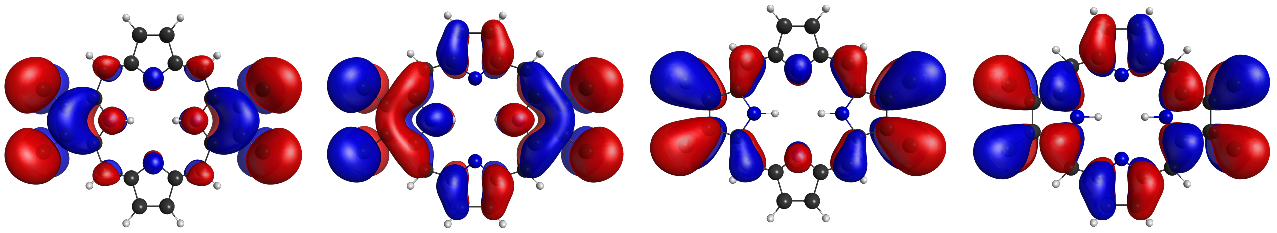

(a) C_0 **(b)** C_1 **(c)** $C_1^{'}$ **(d)** C_2

Figure S4. CASSCF frontier natural orbitals of **PB** with symbolic assignations. Isosurfaces with the value of 0.015.

Table S5. CASSCF(14,14)/cc-pVDZ states of **PB** determined by state-specific calculations using RKS/DFT-optimized geometry. For all the calculations, UHF quintet NOs were used as initial guess. Under HONO, LUNO, etc. three columns represent symmetry of the particular orbital, its symbolic representation[c] according to Figure S4 and NO occupation number.

State	Symmetry	Energy (a.u.)	CASSCF NOs symmetry, identity and occupation number												ΔE from GS (cm^{-1})
			HONO − 1			HONO			LUNO			LUNO + 1			
S_0	A_g	-1137.2141217187	B_{1g}	$C_{1e}^{'}$	1.874	A_u	C_{2i}	1.647	B_{2g}	C_{1i}	0.349	B_{3u}	C_{0e}	0.132	0
T_0	B_{2u}	-1137.1973481963	B_{1g}	$C_{1e}^{'}$	1.878	A_u	C_{2i}	1.003	B_{2g}	C_{1i}	0.997	B_{3u}	C_{0e}	0.126	3681
T_1	B_{1u}	-1137.1278593219	A_u	C_{2i}	1.459	B_{3u}	C_{0i}	1.375	B_{2g}	C_{1i}	0.852	B_{1g}	$C_{1i}^{'}$	0.389	18932
T_2	B_{3g}	-1137.1164331141	B_{1g}	$C_{1e}^{'}$	1.509	A_u	C_{2e}	1.350	B_{3u}	C_{0e}	0.578	B_{1g}	C_{1e}	0.564	21440
S_1	A_g	-1137.0929991918	B_{2g}	$C_{1e}^{'}$	1.544	B_{1g}	C_{1ie}	1.101	A_u	C_{2ie}	0.834	B_{3u}	C_{0e}	0.461	26583
T_3	A_g	-1137.0769821098	A_u	C_{2i}	1.414	B_{3u}	C_{0i}	1.344	B_{1g}	$C_{1e}^{'}$	0.637	B_{2g}	$C_{1i}^{'}$	0.614	30099

Table S6. CASSCF(14,14)/cc-pVDZ states of **PB** determined by state-specific calculations using triplet UKS/DFT-optimized geometry. For all the calculations, UHF quintet NOs were used as initial guess. Under HONO, LUNO, etc. three columns represent symmetry of the particular orbital, its symbolic representation[c] according to Figure S4 and NO occupation number.

State	Symmetry	Energy (a.u.)	CASSCF NOs symmetry, identity and occupation number												ΔE from GS (cm^{-1})
			HONO − 1			HONO			LUNO			LUNO + 1			
S_0	A_g	-1137.2127698680	B_{1g}	$C_{1e}^{'}$	1.869	A_u	C_{2ie}	1.615	B_{2g}	C_{1i}	0.381	B_{3u}	C_{0e}	0.137	0
T_0	B_{2u}	-1137.1978751081	B_{1g}	$C_{1e}^{'}$	1.874	A_u	C_{2i}	1.003	B_{2g}	C_{1i}	0.997	B_{3u}	C_{0e}	0.130	3269
T_1	B_{3g}	-1137.1184024164	B_{1g}	$C_{1e}^{'}$	1.500	A_u	C_{2e}	1.344	B_{3u}	C_{0e}	0.581	B_{2g}	C_{1e}	0.575	20711
T_2	B_{1u}	-1137.1117881786	A_u	C_{2ie}	1.627	B_{3u}	C_{0i}	1.258	B_{2g}	C_{1i}	1.074	B_{2g}	$C_{1i}^{'}$	0.181	22163
S_1	A_g	-1137.0967944603	B_{1g}	$C_{1e}^{'}$	1.540	B_{2g}	C_{1ie}	1.097	A_u	C_{2ie}	0.842	B_{3u}	C_{0e}	0.466	25454
T_3	A_g	-1137.0925423339	B_{1g}	$C_{1e}^{'}$	1.503	B_{2g}	C_{1ie}	1.050	A_u	C_{2ie}	0.900	B_{2g}	C_{1e}	0.506	26387

We also analyzed another tautomer of **PE-Z**, namely **PE-T**, for which the topological analysis also indicates that it must have at least two unpaired electrons in the ground state. This has been verified by the results of CASSCF(16,16)/cc-pVDZ calculations, for which results are given in Tables S10 and S11 according to the symbolic assignations to CASSCF frontier natural orbitals given in Figure S6. This verifies the prediction from the topological analysis with the consistent number of unpaired electrons and ground-state multiplicity. This also

Table S7. CASSCF(14,14)/cc-pVDZ states of **PB** determined by state-specific calculations using quintet UKS/DFT-optimized geometry. For all the calculations, UHF quintet NOs were used as initial guess. Under HONO, LUNO, etc. three columns represent symmetry of the particular orbital, its symbolic representation[c] according to Figure S4 and NO occupation number.

State	Symmetry	Energy (a.u.)	CASSCF NOs symmetry, identity and occupation number												ΔE from GS (cm^{-1})
			HONO − 1			HONO			LUNO			LUNO + 1			
S_0	A_g	-1137.1949270962	B_{1g}	C_{1e}'	1.819	A_u	C_{2ie}	1.639	B_{2g}	C_{1ie}	0.354	B_{3u}	C_{0e}	0.187	0
T_0	B_{2u}	-1137.1772504024	B_{1g}	C_{1e}'	1.832	A_u	C_{2i}	1.007	B_{2g}	C_{1i}	0.994	B_{3u}	C_{0e}	0.173	3880
T_1	B_{3g}	-1137.1286437569	B_{1g}	C_{1e}'	1.491	A_u	C_{2e}	1.286	B_{3u}	C_{0e}	0.653	B_{2g}	C_{1e}	0.574	14548
T_2	B_{1u}	-1137.1078834588	B_{1g}	C_{1e}'	1.659	A_u	C_{2e}	1.069	B_{2g}	C_{1ie}	0.792	B_{2g}	C_{1ie}'	0.443	19104
S_1	A_g	-1137.0993447886	B_{1g}	C_{1e}'	1.497	B_{2g}	C_{1ie}	1.087	A_u	C_{2ie}	0.846	B_{3u}	C_{0e}	0.518	20978
T_3	A_g	-1137.0852410762	B_{1g}	C_{1e}'	1.172	A_u	C_{2ie}	1.033	B_{2g}	C_{1e}	0.964	B_{3u}	C_{0e}	0.838	24073

[c] Auxiliary subscripts in the orbital symbolic notation e refers to the majority of density distribution in external parts of the molecule and i refers to the majority of density distribution in internal parts of the molecule. When both are used, it refers to less distorted distribution, but more density towards the part which comes first in the subscript.

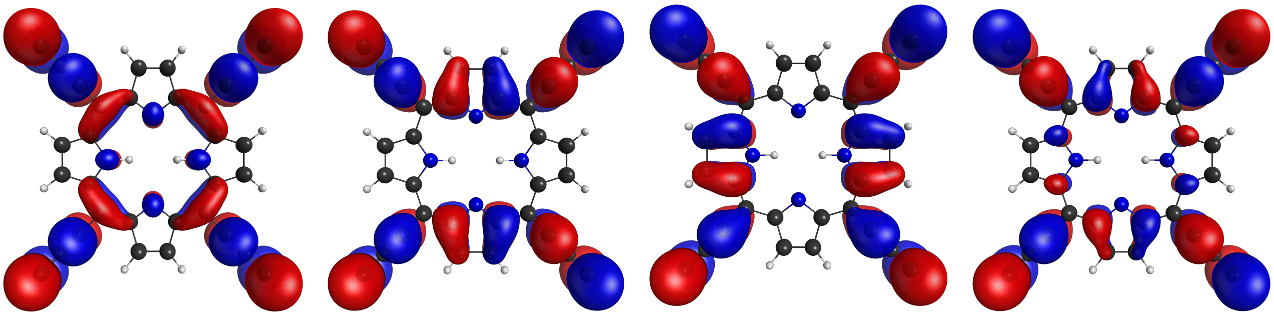

(a) C_0 **(b)** C_1 **(c)** C_1' **(d)** C_2

Figure S5. CASSCF frontier natural orbitals of **PE-Z** with symbolic assignations. Isosurfaces with the value of 0.015.

Table S8. CASSCF(16,16)/cc-pVDZ states of **PE-Z** determined by state-specific calculations using triplet UKS/DFT-optimized geometry. For all the calculations, UHF quintet NOs were used as initial guess. Under HONO, LUNO, etc. three columns represent symmetry of the particular orbital, its symbolic representation according to Figure S5 and NO occupation number.

State	Symmetry	Energy (a.u.)	CASSCF NOs symmetry, identity and occupation number												ΔE from GS (cm^{-1})
			HONO − 1			HONO			LUNO			LUNO + 1			
T_0	B_{2u}	-1439.8413262971	B_{1g}	C_1'	1.793	A_u	C_2	1.000	B_{2g}	C_1	1.000	B_{3u}	C_0	0.194	0
S_0	A_g	-1439.8398065997	B_{1g}	C_1'	1.771	B_{2g}	C_1	1.014	A_u	C_2	0.988	B_{3u}	C_0	0.227	334
T_1	B_{3g}	-1439.8085420696	A_u	C_2	1.276	B_{1g}	C_1'	1.092	B_{2g}	C_1	0.918	B_{3u}	C_0	0.710	7195
T_2	B_{1u}	-1439.8082767286	B_{2g}	C_1	1.439	B_{1g}	C_1'	1.078	A_u	C_2	0.944	B_{3u}	C_0	0.528	7254
Q_0	A_g	-1439.8040310054	B_{1g}	C_1'	1.004	A_u	C_2	1.002	B_{2g}	C_1	1.001	B_{3u}	C_0	0.994	8185
S_1	A_g	N/A													

Table S9. CASSCF(16,16)/cc-pVDZ states of **PE-Z** determined by state-specific calculations using quintet UKS/DFT-optimized geometry. For all the calculations, UHF quintet NOs were used as initial guess. Under HONO, LUNO, etc. three columns represent symmetry of the particular orbital, its symbolic representation according to Figure S5 and NO occupation number.

State	Symmetry	Energy (a.u.)	CASSCF NOs symmetry, identity and occupation number												ΔE from GS (cm^{-1})
			HONO − 1			HONO			LUNO			LUNO + 1			
S_0	A_g	-1439.8341162630	B_{1g}	C_1'	1.637	B_{2g}	C_1	1.018	A_u	C_2	0.988	B_{3u}	C_0	0.355	0
T_0	B_{2u}	-1439.8333618988	B_{1g}	C_1'	1.705	A_u	C_2	1.001	B_{2g}	C_1	1.000	B_{3u}	C_0	0.281	166
T_1	B_{3g}	-1439.8223253632	A_u	C_2	1.171	B_{1g}	C_1'	1.055	B_{2g}	C_1	0.950	B_{3u}	C_0	0.823	2588
T_2	B_{1u}	-1439.8217287705	B_{2g}	C_1	1.180	B_{1g}	C_1'	1.041	A_u	C_2	0.965	B_{3u}	C_0	0.815	2719
Q_0	A_g	-1439.8203846579	B_{1g}	C_1'	1.003	A_u	C_2	1.002	B_{2g}	C_1	1.001	B_{3u}	C_0	0.995	3014
S_1	A_g	N/A													

shows that high-spin diradical porphyrin design with high delocalization of unpaired electrons is persistent across relevant tautomers of the system.

In addition to the tetrasubstituted systems, we created some hexasubstituted systems, among which the most noteworthy are the tautomers of system **QD**, which have four unpaired electrons and high-spin ground-state multiplicity. The topological restriction in tautomers of **QD** shown in Figure S7a is tight and diradical and tetraradical characters approach 1 because the closest radicalogen groups are bonded to atoms in 1, 3 positions from one another and closing the shell between them via through-space interaction is unfavorable, especially because such a bond would be inside a six-membered ring and cause higher repulsion than a proper π bond. As

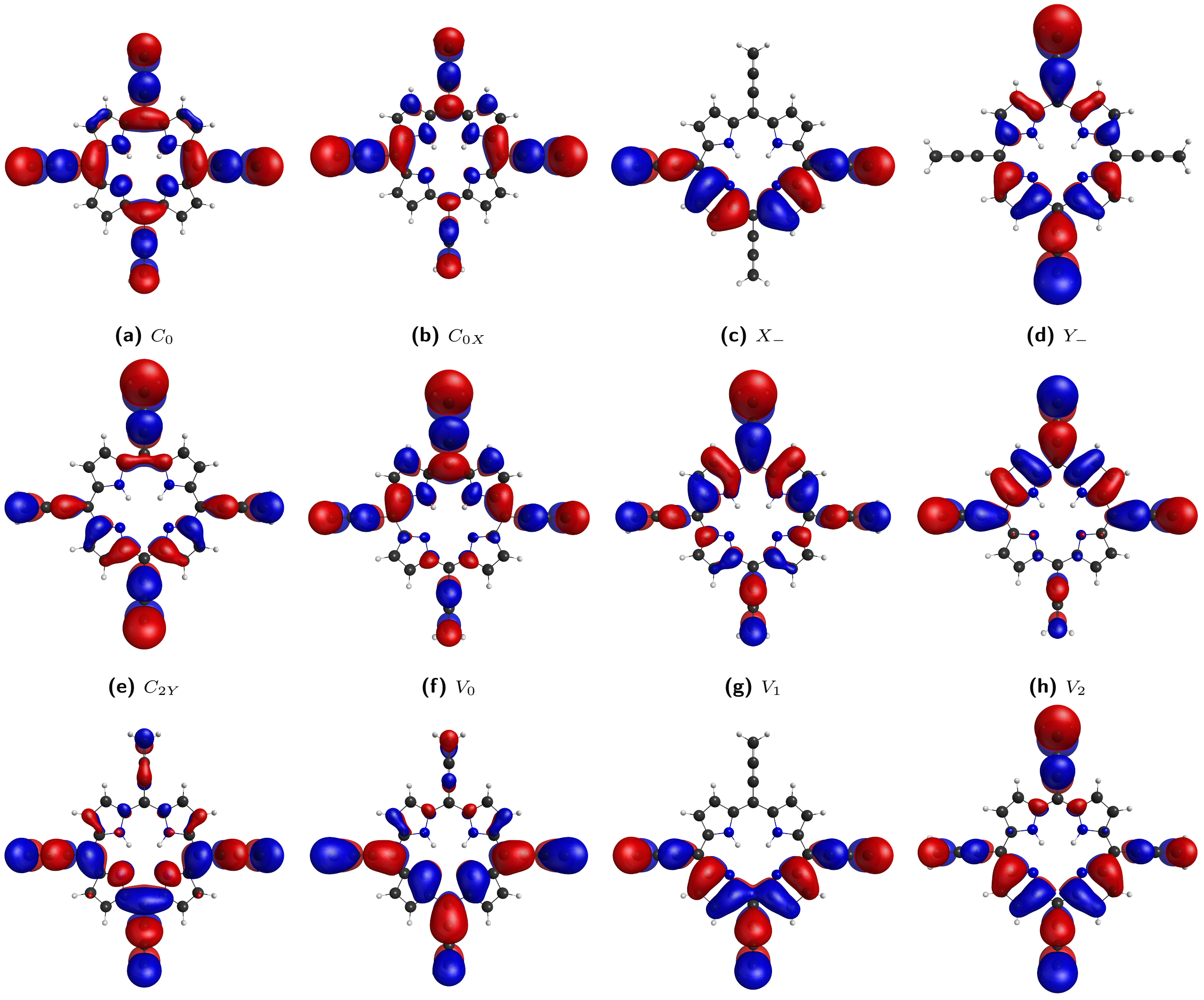

(a) C_0 **(b)** C_{0X} **(c)** X_- **(d)** Y_-

(e) C_{2Y} **(f)** V_0 **(g)** V_1 **(h)** V_2

(i) W_0 **(j)** W_1 **(k)** W_2 **(l)** $V_1 + W_1$

Figure S6. CASSCF frontier natural orbitals of **PE-T** with symbolic assignations. Isosurfaces with the value of 0.015.

Table S10. CASSCF(16,16)/cc-pVDZ states of **PE-T** determined by state-specific calculations using triplet UKS/DFT-optimized geometry. For all the calculations, UHF quintet NOs were used as initial guess. Under HONO, LUNO, etc. three columns represent symmetry of the particular orbital, its symbolic representation according to Figure S6 and NO occupation number.

State	Symmetry	Energy (a.u.)	CASSCF NOs symmetry, identity and occupation number												ΔE from GS (cm^{-1})
			HONO − 1			HONO			LUNO			LUNO + 1			
T_0	B_1	-1439.8499103419	B_2	W_1	1.792	A_2	X_-	1.003	B_2	W_2	0.998	B_2	W_0	0.214	0.00
S_0	A_1	-1439.8433582546	B_2	W_1	1.834	A_2	X_-	1.093	B_2	W_2	0.911	B_2	W_0	0.162	4.11
Q_0	B_1	-1439.8047056171	B_2	V_1	1.012	A_2	X_-	1.004	B_2	W_2	1.000	B_2	V_0	0.979	28.37
T_1	A_1	-1439.8029892080	A_2	X_-	1.323	B_2	V_1	1.155	B_2	W_2	1.025	B_2	C_0	0.523	29.44
S_1	A_1	-1439.7945635540	A_2	X_-	1.410	B_2	V_2	1.327	B_2	V_1	0.936	B_2	C_0	0.394	12147.22
T_2	B_1	N/A													

apparent from the frontier orbitals and their occupation numbers for the tautomers of **QD** in Figures S7c to S7e, the unpaired electrons are fully delocalized in the molecule, which should be favorable for the chemical stability of the **QD**. Notably, one could also predict the ground state multiplicity of these tautomers based on the careful extension of Ovchinnikov's rule by following the proper through-bond path between radical centers as described in our previous works.[19,20]

Furthermore, we investigated the deprotonated species for the tetraradical system **QD**, as in the dynamic systems with different conditions, the deprotonation can be relevant and it is important to analyze its effects on the electronic structure of the compound. According to the resonance structures of singly deprotonated (net charge -1) **QD**, it is also topologically constrained to have at least four unpaired electrons, as shown in Figure S8a and verified by CASCI(14,14)/cc-pVDZ spectra in Figure S8b. In the figures for singly deprotonated species, when

Table S11. CASSCF(16,16)/cc-pVDZ states of **PE-T** determined by state-specific calculations using quintet UKS/DFT-optimized geometry. For all the calculations, UHF quintet NOs were used as initial guess. Under HONO, LUNO, etc. three columns represent symmetry of the particular orbital, its symbolic representation according to Figure S5 and NO occupation number.

State	Symmetry	Energy (a.u.)	CASSCF NOs symmetry, identity and occupation number												ΔE from GS (cm^{-1})
			HONO − 1			HONO			LUNO			LUNO + 1			
T_0	B_1	-1439.8395401692	B_2	V_1	1.682	A_2	X_-	1.003	B_2	W_2	0.999	B_2	C_0	0.320	0.00
S_0	A_1	-1439.8325805048	B_2	V_1	1.739	A_2	X_-	1.069	B_2	W_2	0.939	B_2	W_0	0.261	4.37
Q_0	B_1	-1439.8178050621	B_2	V_1	1.013	A_2	X_-	1.004	B_2	W_2	1.000	B_2	V_0	0.985	13.64
T_1	A_1	-1439.8145194439	A_2	X_-	1.281	B_2	C_{2Y}	1.027	B_2	Y_-	1.019	B_2	C_{0X}	0.680	15.70
T_2	B_1	-1439.8094665330	B_2	W_2	1.146	B_2	V_1	1.033	A_2	X_-	1.017	B_2	C_0	0.811	6600.40
S_1	A_1	-1439.8005406412	A_2	X_-	1.479	B_2	C_2	1.286	B_2	$V_1 + W_1$	0.830	B_2	C_0	0.428	8559.41

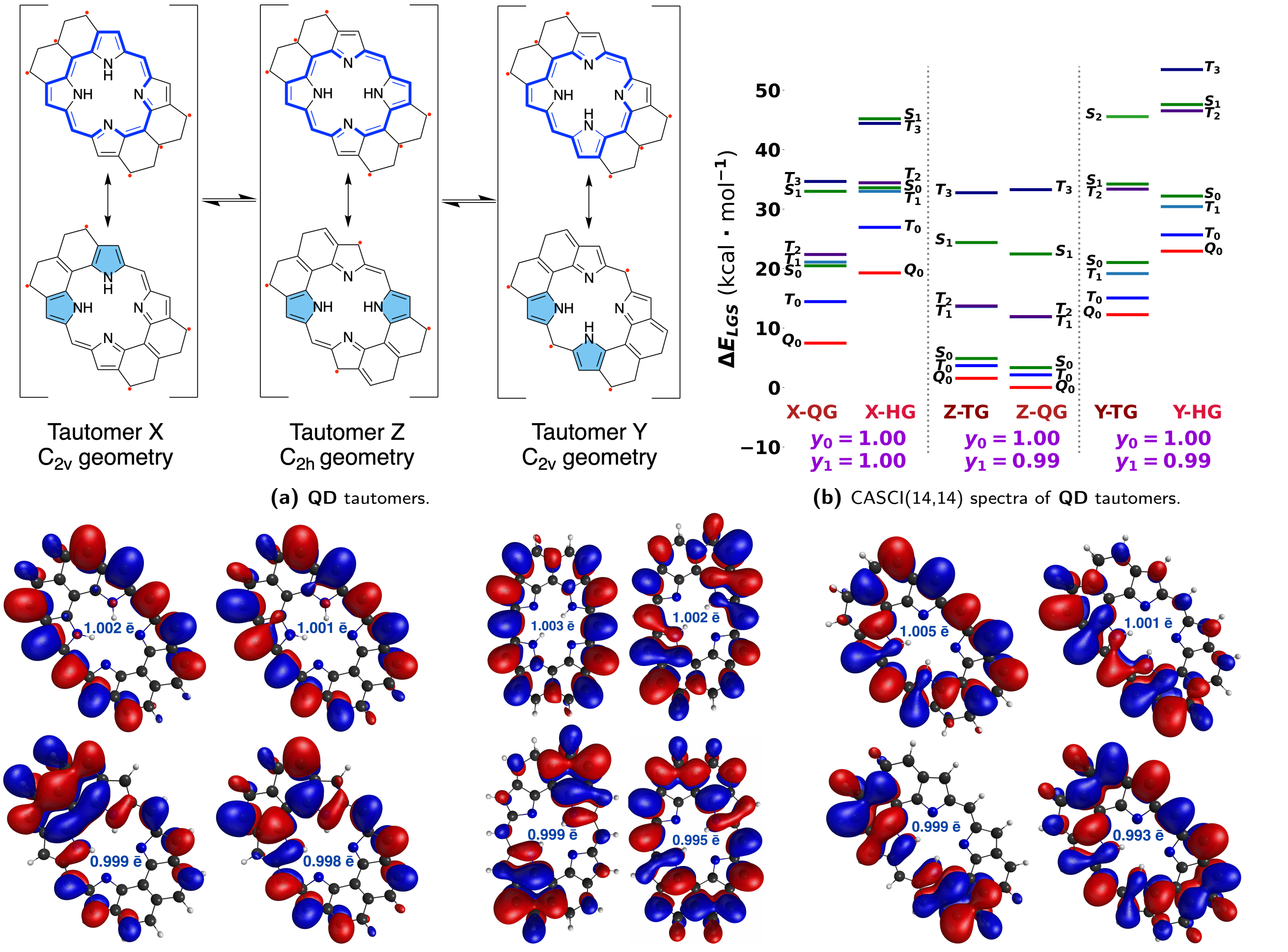

(a) **QD** tautomers. (b) CASCI(14,14) spectra of **QD** tautomers.

(c) **QD-X** frontier NOs. (d) **QD-Z** frontier NOs. (e) **QD-Y** frontier NOs.

Figure S7. (a) Tautomer structures of **QD**, (b) CASCI spectra using UKS-triplet, UKS-quintet, UKS-septet-optimized geometries (TG, QG, and HG, respectively) with diradical (y_0) and tetraradical (y_1) characters, and (c, d, e) frontier NOs of **QD-X**, **QD-Z**, **QD-Y** with occupation numbers.

the nitrogen has two lone pairs, only the π lone pair is marked with the bar. It is noteworthy that spectral gaps between spin states and the overall spectral range of the low-energy spectrum are diminished upon deprotonation, but the ground state multiplicity remains a quintet.

The significantly different electronic structure is obtained with double deprotonation of **QD** (net charge −2), because the four nitrogen-containing rings become essentially equivalent and the greater repulsion leads to shifting electron density away from the pyrrole rings, which loosens topological restrictions. The analysis with RSs given in Figure S9a shows the nominal topological restriction to have at least four unpaired electrons, which is consistent with CASCI(14,14)/cc-pVDZ results given in Figure S9b and calculated diradical and tetraradical character indices. Nonetheless, due to the negative charge in the core of the compound, the unpaired electron density is repelled more towards the peripheries of the molecule and this increases through-space overlap between orbitals that bear these unpaired electrons, leading to the contribution from the configuration with long-range through-space on-bond pairing due to nonzero overlap between participating π-orbitals. Hence, double deprotonation and

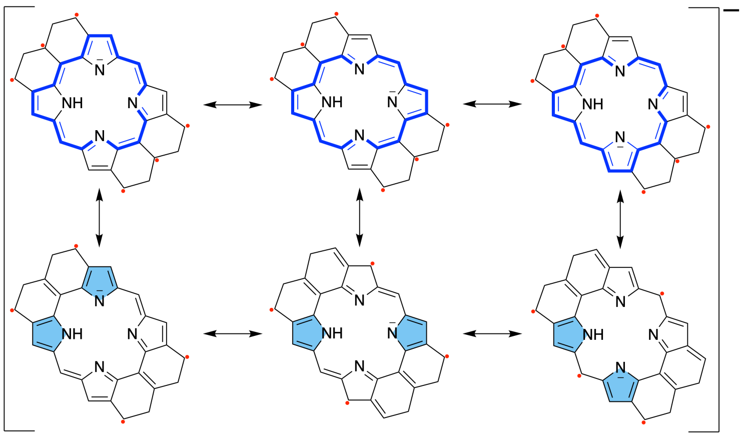

(a) Resonance structure of $\mathbf{QD}^-_{m1H}$

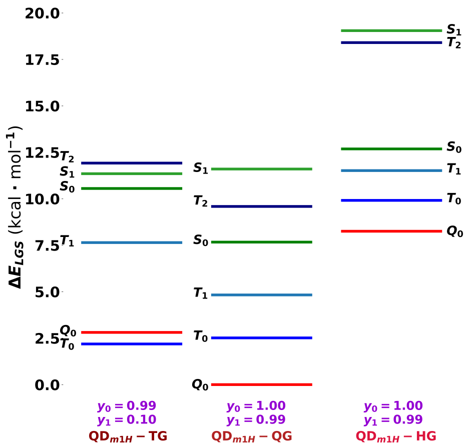

(b) CASCI(14,14) spectra of $\mathbf{QD}^-_{m1H}$

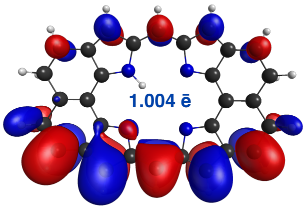

(c)

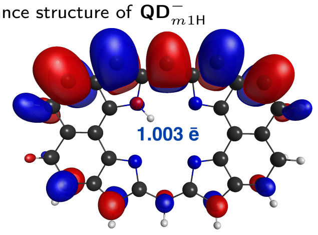

(d)

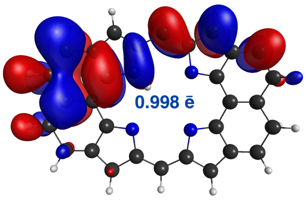

(e)

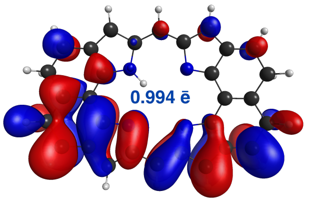

(f)

Figure S8. (a) Resonance structures of singly deprotonated (with charge -1) **QD**. (b) CASCI spectra using UKS-triplet, UKS-quintet, UKS-septet-optimized geometries (TG, QG, and HG, respectively) with diradical (y_0) and tetraradical (y_1) characters. (c-f) CASCI quintet ground state four singly occupied natural orbitals, computed at quintet UKS-optimized geometry.

thus making some parts of the compound equivalent that were not before may cause a topological change in the π-system that can affect the previously imposed restriction. It is noteworthy that the underlying topological structure is still present in the doubly deprotonated species and the tetraradical resonance structure is still being stabilized by the deprotonated pyrrole rings in the aromatic configurations. Also, the proper tetraradical(oid) structure of the low-energy spectrum is maintained (see definitions in our recent work [19]). This can even be a beneficial feature of this open-shell porphyrin system, because doubly deprotonated species can be an important intermediate in preparation of the metal-inserted open-shell porphyrins. Since the open-shell character of this doubly deprotonated species is diminished, the tendencies for side, radical-like reactions for this intermediate are commensurately diminished.

We further examined the effect of the insertion of the main group metal ion Mg^{2+} in the doubly deprotonated porphyrin, so that the overall metal-porphyrin complex is neutral. The topological analysis with RSs in Figure S10a shows at least four unpaired electrons, which is verified by the CASCI(14,14)/cc-pVDZ calculation results and diradical and tetraradical character indices shown in Figure S10b. Furthermore, the spectral ranges are more diminished than in singly and deprotonated species and the ground state multiplicity and number of unpaired electrons are firmly maintained from the original system **QD**.

We can see the tendency that the accumulation of more negative charge in the system can loosen the topological restriction imposed in the neutral species (protonated), while the inclusion of the metal ion in the doubly deprotonated species tightens the topological restriction on the number of unpaired electrons and maintains ground-state multiplicity from the neutral species, as well as correspondence of the low-energy spectrum with the assumed polyradical character. For the systems of the same symmetry, such as doubly deprotonated and metal-inserted species, the abundance in electron density for the core of the porphyrin relates to spectral ranges of the spin states. Based on the discussed cases, one could hypothesize the trend that the higher the electron density in the core, the more likely it is to loosen the topological restriction and change the GS multiplicity of the original species.

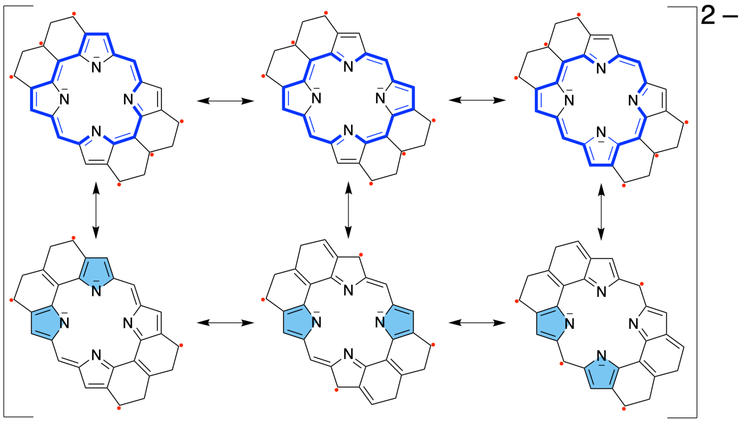

(a) Resonance structure of QD^{2-}_{m2H}

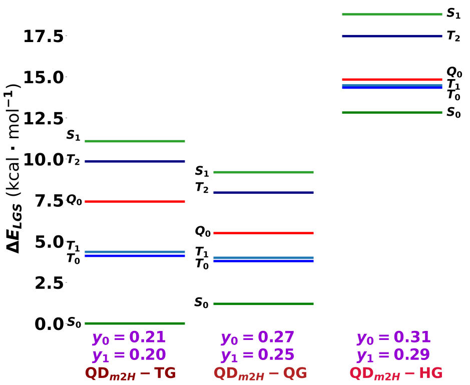

(b) CASCI(14,14) spectra of QD^{2-}_{m2H}

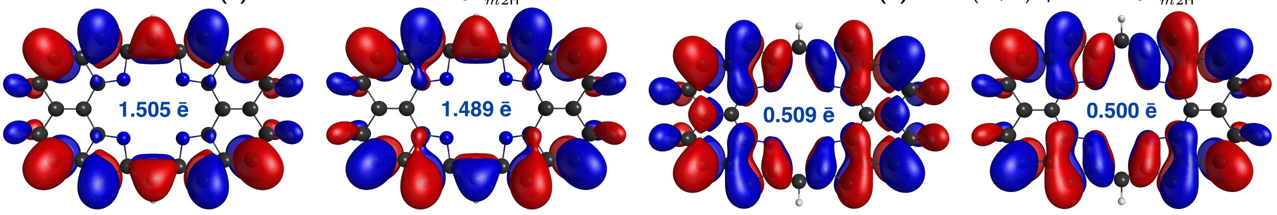

(c) (d) (e) (f)

Figure S9. (a) Resonance structures of doubly deprotonated (with charge −2) **QD**. (b) CASCI spectra using UKS-triplet, UKS-quintet, UKS-septet-optimized geometries (TG, QG, and HG, respectively) with diradical (y_0) and tetraradical (y_1) characters. (c-f) CASCI singlet ground state frontier NOs with occupation numbers, computed at quintet UKS-optimized geometry.

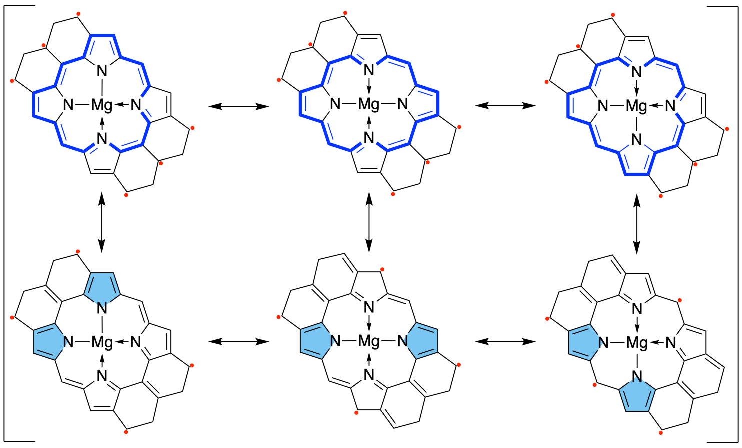

(a) Resonance structure of Mg-**QD**.

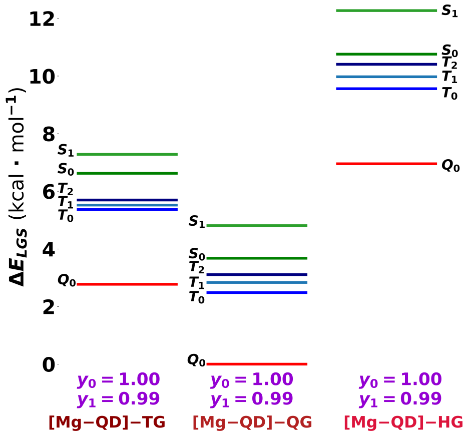

(b) CASCI(14,14) spectra of Mg-**QD**.

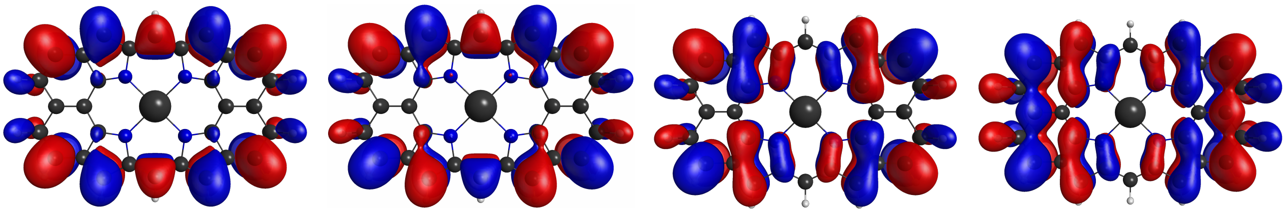

$n_{HONO-1} = 1.000$ $n_{HONO} = 1.000$ $n_{LUNO} = 0.998$ $n_{LUNO+1} = 0.994$

(c) **Mg-QD** frontier **singly occupied** NOs.

Figure S10. (a) Resonance structures of Mg^{2+} inserted **QD**. (b) CASCI spectra of **Mg-QD** using UKS-triplet, UKS-quintet, UKS-septet-optimized geometries (TG, QG, and HG, respectively) with diradical (y_0) and tetraradical (y_1) characters. (c) CASCI quintet ground state four singly occupied natural orbitals, computed at quintet UKS-optimized geometry.

S4. Optimized Geometries

The RKS/DFT-optimized geometry of molecule **L1N** is given below (BLYP/cc-pVTZ level of theory).
Total electronic energy: -802.548966947 $a.u.$

32

N	0.0000000000	-0.0000000000	2.8303690000
C	0.0000000000	-0.0000000000	1.4096270000
C	0.0000000000	1.2543320000	0.7194190000
C	0.0000000000	2.4705450000	1.4815570000
C	0.0000000000	2.4179090000	2.8599850000
H	0.0000000000	1.1023170000	-4.6063580000
C	0.0000000000	-1.1972700000	3.5276460000
C	-0.0000000000	-2.4179090000	2.8599850000
H	0.0000000000	-3.4315450000	-0.9827640000
C	0.0000000000	1.1972700000	3.5276460000
C	-0.0000000000	-1.2543320000	0.7194190000
C	-0.0000000000	-1.2543320000	-0.7194190000
C	-0.0000000000	-2.4705450000	-1.4815570000
C	-0.0000000000	-2.4179090000	-2.8599850000
H	0.0000000000	3.3276370000	-3.4550150000
H	-0.0000000000	-3.3276370000	-3.4550150000
H	0.0000000000	3.4315450000	-0.9827640000
H	-0.0000000000	-3.4315450000	0.9827640000
C	0.0000000000	-1.1972700000	-3.5276460000
N	0.0000000000	-0.0000000000	-2.8303690000
C	0.0000000000	-0.0000000000	-1.4096270000
C	0.0000000000	1.2543320000	-0.7194190000
C	0.0000000000	2.4705450000	-1.4815570000
C	0.0000000000	2.4179090000	-2.8599850000
C	0.0000000000	1.1972700000	-3.5276460000
C	-0.0000000000	-2.4705450000	1.4815570000
H	0.0000000000	3.4315450000	0.9827640000
H	-0.0000000000	3.3276370000	3.4550150000
H	-0.0000000000	-1.1023170000	-4.6063580000
H	-0.0000000000	-3.3276370000	3.4550150000
H	-0.0000000000	-1.1023170000	4.6063580000
H	-0.0000000000	1.1023170000	4.6063580000

The triplet UKS/DFT-optimized geometry of molecule **L1N** is given below (BLYP/cc-pVTZ level of theory). Total electronic energy: -802.534958887 $a.u.$

32

N	0.0000000000	0.0000000000	2.8273910000
C	0.0000000000	0.0000000000	1.4076310000
C	0.0000000000	1.2525970000	0.7252180000
C	0.0000000000	2.4578620000	1.4776120000
C	0.0000000000	2.4089080000	2.8806000000
H	0.0000000000	1.1031860000	-4.6185270000
C	0.0000000000	-1.2055690000	3.5401390000
C	-0.0000000000	-2.4089080000	2.8806000000
H	0.0000000000	-3.4194470000	-0.9805610000
C	0.0000000000	1.2055690000	3.5401390000
C	-0.0000000000	-1.2525970000	0.7252180000
C	-0.0000000000	-1.2525970000	-0.7252180000
C	-0.0000000000	-2.4578620000	-1.4776120000
C	-0.0000000000	-2.4089080000	-2.8806000000
H	0.0000000000	3.3235030000	-3.4677770000
H	-0.0000000000	-3.3235030000	-3.4677770000
H	0.0000000000	3.4194470000	-0.9805610000
H	-0.0000000000	-3.4194470000	0.9805610000

```
C      0.0000000000   -1.2055690000   -3.5401390000
N      0.0000000000   -0.0000000000   -2.8273910000
C      0.0000000000   -0.0000000000   -1.4076310000
C      0.0000000000    1.2525970000   -0.7252180000
C      0.0000000000    2.4578620000   -1.4776120000
C      0.0000000000    2.4089080000   -2.8806000000
C      0.0000000000    1.2055690000   -3.5401390000
C     -0.0000000000   -2.4578620000    1.4776120000
H      0.0000000000    3.4194470000    0.9805610000
H     -0.0000000000    3.3235030000    3.4677770000
H     -0.0000000000   -1.1031860000   -4.6185270000
H     -0.0000000000   -3.3235030000    3.4677770000
H     -0.0000000000   -1.1031860000    4.6185270000
H     -0.0000000000    1.1031860000    4.6185270000
```

The RKS/DFT-optimized geometry of molecule **L1B** is given below (BLYP/cc-pVTZ level of theory). Total electronic energy: -742.741650291 $a.u.$

```
32

B     -0.0000000000    0.0000000000    2.9721510000
C     -0.0000000000    0.0000000000    1.4363480000
C      0.0000000000    1.2401490000    0.7237100000
C      0.0000000000    2.4681270000    1.4804960000
C      0.0000000000    2.5205940000    2.8718730000
H      0.0000000000    1.4840930000   -4.7440040000
C      0.0000000000   -1.3518240000    3.6592590000
C     -0.0000000000   -2.5205940000    2.8718730000
H      0.0000000000   -3.4138010000   -0.9480130000
C      0.0000000000    1.3518240000    3.6592590000
C     -0.0000000000   -1.2401490000    0.7237100000
C     -0.0000000000   -1.2401490000   -0.7237100000
C     -0.0000000000   -2.4681270000   -1.4804960000
C     -0.0000000000   -2.5205940000   -2.8718730000
H      0.0000000000    3.5063570000   -3.3401480000
H     -0.0000000000   -3.5063570000   -3.3401480000
H      0.0000000000    3.4138010000   -0.9480130000
H     -0.0000000000   -3.4138010000    0.9480130000
C      0.0000000000   -1.3518240000   -3.6592590000
B      0.0000000000    0.0000000000   -2.9721510000
C      0.0000000000    0.0000000000   -1.4363480000
C      0.0000000000    1.2401490000   -0.7237100000
C      0.0000000000    2.4681270000   -1.4804960000
C      0.0000000000    2.5205940000   -2.8718730000
C      0.0000000000    1.3518240000   -3.6592590000
C     -0.0000000000   -2.4681270000    1.4804960000
H      0.0000000000    3.4138010000    0.9480130000
H     -0.0000000000    3.5063570000    3.3401480000
H     -0.0000000000   -1.4840930000   -4.7440040000
H     -0.0000000000   -3.5063570000    3.3401480000
H     -0.0000000000   -1.4840930000    4.7440040000
H     -0.0000000000    1.4840930000    4.7440040000
```

The triplet UKS/DFT-optimized geometry of molecule **L1B** is given below (BLYP/cc-pVTZ level of theory). Total electronic energy: -742.728503389 $a.u.$

```
32
```

B	0.0000000000	0.0000000000	2.9893380000
C	0.0000000000	0.0000000000	1.4389380000
C	0.0000000000	1.2404750000	0.7306090000
C	0.0000000000	2.4520330000	1.4812410000
C	0.0000000000	2.5135470000	2.8939880000
H	0.0000000000	1.5024890000	-4.7656460000
C	0.0000000000	-1.3702850000	3.6814340000
C	-0.0000000000	-2.5135470000	2.8939880000
H	0.0000000000	-3.4004960000	-0.9524760000
C	0.0000000000	1.3702850000	3.6814340000
C	-0.0000000000	-1.2404750000	0.7306090000
C	-0.0000000000	-1.2404750000	-0.7306090000
C	-0.0000000000	-2.4520330000	-1.4812410000
C	-0.0000000000	-2.5135470000	-2.8939880000
H	0.0000000000	3.5066230000	-3.3470500000
H	-0.0000000000	-3.5066230000	-3.3470500000
H	0.0000000000	3.4004960000	-0.9524760000
H	-0.0000000000	-3.4004960000	0.9524760000
C	0.0000000000	-1.3702850000	-3.6814340000
B	0.0000000000	0.0000000000	-2.9893380000
C	0.0000000000	0.0000000000	-1.4389380000
C	0.0000000000	1.2404750000	-0.7306090000
C	0.0000000000	2.4520330000	-1.4812410000
C	0.0000000000	2.5135470000	-2.8939880000
C	0.0000000000	1.3702850000	-3.6814340000
C	-0.0000000000	-2.4520330000	1.4812410000
H	0.0000000000	3.4004960000	0.9524760000
H	-0.0000000000	3.5066230000	3.3470500000
H	-0.0000000000	-1.5024890000	-4.7656460000
H	-0.0000000000	-3.5066230000	3.3470500000
H	-0.0000000000	-1.5024890000	4.7656460000
H	-0.0000000000	1.5024890000	4.7656460000

The RKS/DFT-optimized geometry of molecule **L3O** is given below (BLYP/cc-pVTZ level of theory).
Total electronic energy: −1145.21226563 *a.u.*

36

C	-0.0000000000	0.0000000000	2.8304740000
C	-0.0000000000	0.0000000000	1.3898490000
C	-0.0000000000	1.2695410000	0.7350670000
C	-0.0000000000	2.4144310000	1.4916030000
O	-0.0000000000	2.4219310000	2.8816140000
C	-0.0000000000	0.0000000000	5.6375790000
C	-0.0000000000	-1.2080610000	3.5558920000
H	-0.0000000000	3.4272480000	1.1114270000
C	-0.0000000000	1.2223590000	4.9511370000
C	0.0000000000	1.2080610000	3.5558920000
C	-0.0000000000	-1.2695410000	0.7350670000
C	-0.0000000000	-1.2695410000	-0.7350670000
C	-0.0000000000	-2.4144310000	-1.4916030000
O	-0.0000000000	-2.4219310000	-2.8816140000
H	0.0000000000	3.4272480000	-1.1114270000
H	-0.0000000000	0.0000000000	6.7248830000
C	-0.0000000000	-1.2223590000	4.9511370000
C	-0.0000000000	-1.2223590000	-4.9511370000
C	0.0000000000	-1.2080610000	-3.5558920000

```
C      0.0000000000    0.0000000000   -2.8304740000
C      0.0000000000    0.0000000000   -1.3898490000
C      0.0000000000    1.2695410000   -0.7350670000
C      0.0000000000    2.4144310000   -1.4916030000
O      0.0000000000    2.4219310000   -2.8816140000
H      0.0000000000    0.0000000000   -6.7248830000
H     -0.0000000000   -3.4272480000   -1.1114270000
H     -0.0000000000   -3.4272480000    1.1114270000
C      0.0000000000    1.2223590000   -4.9511370000
C      0.0000000000    0.0000000000   -5.6375790000
C      0.0000000000    1.2080610000   -3.5558920000
C     -0.0000000000   -2.4144310000    1.4916030000
O     -0.0000000000   -2.4219310000    2.8816140000
H      0.0000000000   -2.1715160000   -5.4797240000
H      0.0000000000    2.1715160000    5.4797240000
H      0.0000000000    2.1715160000   -5.4797240000
H     -0.0000000000   -2.1715160000    5.4797240000
```

The triplet UKS/DFT-optimized geometry of molecule **L3O** is given below (BLYP/cc-pVTZ level of theory). Total electronic energy: -1145.20515566 *a.u.*

```
36

C     -0.0000000000   -0.0000000000    2.8430160000
C     -0.0000000000   -0.0000000000    1.4019300000
C     -0.0000000000    1.2703430000    0.7415230000
C     -0.0000000000    2.4123660000    1.5063380000
O     -0.0000000000    2.4210160000    2.8978350000
C     -0.0000000000   -0.0000000000    5.6534290000
C     -0.0000000000   -1.2065170000    3.5704520000
H     -0.0000000000    3.4271230000    1.1301050000
C     -0.0000000000    1.2213450000    4.9656380000
C      0.0000000000    1.2065170000    3.5704520000
C     -0.0000000000   -1.2703430000    0.7415230000
C     -0.0000000000   -1.2703430000   -0.7415230000
C     -0.0000000000   -2.4123660000   -1.5063380000
O     -0.0000000000   -2.4210160000   -2.8978350000
H      0.0000000000    3.4271230000   -1.1301050000
H     -0.0000000000   -0.0000000000    6.7406800000
C     -0.0000000000   -1.2213450000    4.9656380000
C     -0.0000000000   -1.2213450000   -4.9656380000
C      0.0000000000   -1.2065170000   -3.5704520000
C      0.0000000000   -0.0000000000   -2.8430160000
C      0.0000000000   -0.0000000000   -1.4019300000
C      0.0000000000    1.2703430000   -0.7415230000
C      0.0000000000    2.4123660000   -1.5063380000
O      0.0000000000    2.4210160000   -2.8978350000
H      0.0000000000   -0.0000000000   -6.7406800000
H     -0.0000000000   -3.4271230000   -1.1301050000
H     -0.0000000000   -3.4271230000    1.1301050000
C      0.0000000000    1.2213450000   -4.9656380000
C      0.0000000000   -0.0000000000   -5.6534290000
C      0.0000000000    1.2065170000   -3.5704520000
C     -0.0000000000   -2.4123660000    1.5063380000
O     -0.0000000000   -2.4210160000    2.8978350000
H      0.0000000000   -2.1713780000   -5.4926320000
H      0.0000000000    2.1713780000    5.4926320000
H      0.0000000000    2.1713780000   -5.4926320000
```

```
H     -0.0000000000   -2.1713780000    5.4926320000
```

The RKS/DFT-optimized geometry of molecule **L3H** is given below (BLYP/cc-pVTZ level of theory).
Total electronic energy: −1001.44698188 *a.u.*

```
44

C      2.9180050000   -0.0000000000    0.0000000000
C      1.4489550000   -0.0000000000    0.0000000000
C      0.7466710000    1.2551800000   -0.0000000000
C      1.4389630000    2.4413720000   -0.0000000000
C      2.9268830000    2.5461530000   -0.0000000000
C      5.7559700000   -0.0000000000    0.0000000000
C      3.6504920000   -1.2194670000    0.0000000000
H      0.9098160000    3.3875110000   -0.0000000000
C      5.0487920000    1.2042860000   -0.0000000000
C      3.6504920000    1.2194670000    0.0000000000
C      0.7466710000   -1.2551800000   -0.0000000000
C     -0.7466710000   -1.2551800000   -0.0000000000
C     -1.4389630000   -2.4413720000   -0.0000000000
C     -2.9268830000   -2.5461530000   -0.0000000000
H     -0.9098160000    3.3875110000   -0.0000000000
H      6.8440890000   -0.0000000000    0.0000000000
C      5.0487920000   -1.2042860000   -0.0000000000
C     -5.0487920000   -1.2042860000   -0.0000000000
C     -3.6504920000   -1.2194670000   -0.0000000000
C     -2.9180050000    0.0000000000    0.0000000000
C     -1.4489550000    0.0000000000    0.0000000000
C     -0.7466710000    1.2551800000   -0.0000000000
C     -1.4389630000    2.4413720000   -0.0000000000
C     -2.9268830000    2.5461530000   -0.0000000000
H     -6.8440890000    0.0000000000    0.0000000000
H     -0.9098160000   -3.3875110000   -0.0000000000
H      0.9098160000   -3.3875110000   -0.0000000000
C     -5.0487920000    1.2042860000   -0.0000000000
C     -5.7559700000    0.0000000000    0.0000000000
C     -3.6504920000    1.2194670000    0.0000000000
C      1.4389630000   -2.4413720000   -0.0000000000
C      2.9268830000   -2.5461530000   -0.0000000000
H     -5.5892670000   -2.1507550000    0.0000000000
H      5.5892670000    2.1507550000    0.0000000000
H     -5.5892670000    2.1507550000    0.0000000000
H      5.5892670000   -2.1507550000    0.0000000000
H      3.2561620000    3.1465830000    0.8695190000
H      3.2561620000    3.1465830000   -0.8695190000
H     -3.2561620000   -3.1465830000    0.8695190000
H     -3.2561620000   -3.1465830000   -0.8695190000
H     -3.2561620000    3.1465830000   -0.8695190000
H     -3.2561620000    3.1465830000    0.8695190000
H      3.2561620000   -3.1465830000    0.8695190000
H      3.2561620000   -3.1465830000   -0.8695190000
```

The triplet UKS/DFT-optimized geometry of molecule **L3H** is given below (BLYP/cc-pVTZ level of theory).
Total electronic energy: −1001.44174650 *a.u.*

```
44
```

C	2.9294500000	-0.0000000000	0.0000000000
C	1.4607710000	-0.0000000000	0.0000000000
C	0.7523880000	1.2569630000	-0.0000000000
C	1.4539100000	2.4389380000	-0.0000000000
C	2.9412190000	2.5449650000	-0.0000000000
C	5.7698530000	-0.0000000000	0.0000000000
C	3.6636500000	-1.2185900000	0.0000000000
H	0.9277190000	3.3882220000	-0.0000000000
C	5.0619160000	1.2037520000	-0.0000000000
C	3.6636500000	1.2185900000	0.0000000000
C	0.7523880000	-1.2569630000	-0.0000000000
C	-0.7523880000	-1.2569630000	-0.0000000000
C	-1.4539100000	-2.4389380000	-0.0000000000
C	-2.9412190000	-2.5449650000	-0.0000000000
H	-0.9277190000	3.3882220000	-0.0000000000
H	6.8579510000	-0.0000000000	0.0000000000
C	5.0619160000	-1.2037520000	-0.0000000000
C	-5.0619160000	-1.2037520000	-0.0000000000
C	-3.6636500000	-1.2185900000	-0.0000000000
C	-2.9294500000	0.0000000000	0.0000000000
C	-1.4607710000	0.0000000000	0.0000000000
C	-0.7523880000	1.2569630000	-0.0000000000
C	-1.4539100000	2.4389380000	-0.0000000000
C	-2.9412190000	2.5449650000	-0.0000000000
H	-6.8579510000	0.0000000000	0.0000000000
H	-0.9277190000	-3.3882220000	-0.0000000000
H	0.9277190000	-3.3882220000	-0.0000000000
C	-5.0619160000	1.2037520000	-0.0000000000
C	-5.7698530000	0.0000000000	0.0000000000
C	-3.6636500000	1.2185900000	0.0000000000
C	1.4539100000	-2.4389380000	-0.0000000000
C	2.9412190000	-2.5449650000	-0.0000000000
H	-5.6016300000	-2.1506820000	0.0000000000
H	5.6016300000	2.1506820000	0.0000000000
H	-5.6016300000	2.1506820000	0.0000000000
H	5.6016300000	-2.1506820000	0.0000000000
H	3.2699430000	3.1460250000	0.8696860000
H	3.2699430000	3.1460250000	-0.8696860000
H	-3.2699430000	-3.1460250000	0.8696860000
H	-3.2699430000	-3.1460250000	-0.8696860000
H	-3.2699430000	3.1460250000	-0.8696860000
H	-3.2699430000	3.1460250000	0.8696860000
H	3.2699430000	-3.1460250000	0.8696860000
H	3.2699430000	-3.1460250000	-0.8696860000

The RKS/DFT-optimized geometry of molecule **CB** is given below (BLYP/cc-pVTZ level of theory).
Total electronic energy: -386.914500660 *a.u.*

20

H	0.0000000000	3.4097880000	1.0173570000
C	0.0000000000	-0.0000000000	1.3912760000
C	0.0000000000	-1.2637400000	0.7455430000
C	0.0000000000	-2.4280530000	1.4760360000
C	0.0000000000	-1.2637400000	-0.7455430000
C	0.0000000000	-0.0000000000	-1.3912760000
C	0.0000000000	2.4280530000	1.4760360000

```
C      0.0000000000   -2.4280530000   -1.4760360000
H      0.0000000000    3.4097880000   -1.0173570000
H     -0.0000000000   -3.4097880000    1.0173570000
C      0.0000000000    1.2637400000    0.7455430000
C      0.0000000000    1.2637400000   -0.7455430000
C      0.0000000000    2.4280530000   -1.4760360000
H     -0.0000000000   -3.4097880000   -1.0173570000
H      0.0000000000   -0.0000000000    2.4818490000
H      0.0000000000   -2.3952090000    2.5629160000
H      0.0000000000   -0.0000000000   -2.4818490000
H      0.0000000000    2.3952090000    2.5629160000
H      0.0000000000   -2.3952090000   -2.5629160000
H      0.0000000000    2.3952090000   -2.5629160000
```

The triplet UKS/DFT-optimized geometry of molecule **CB** is given below (BLYP/cc-pVTZ level of theory). Total electronic energy: -386.912402769 *a.u.*

```
20

H      0.0000000000    3.4107880000    1.0584610000
C      0.0000000000   -0.0000000000    1.4026380000
C      0.0000000000   -1.2646400000    0.7517850000
C      0.0000000000   -2.4203090000    1.5008740000
C      0.0000000000   -1.2646400000   -0.7517850000
C      0.0000000000   -0.0000000000   -1.4026380000
C      0.0000000000    2.4203090000    1.5008740000
C      0.0000000000   -2.4203090000   -1.5008740000
H      0.0000000000    3.4107880000   -1.0584610000
H     -0.0000000000   -3.4107880000    1.0584610000
C      0.0000000000    1.2646400000    0.7517850000
C      0.0000000000    1.2646400000   -0.7517850000
C      0.0000000000    2.4203090000   -1.5008740000
H     -0.0000000000   -3.4107880000   -1.0584610000
H      0.0000000000    0.0000000000    2.4924950000
H      0.0000000000   -2.3696970000    2.5868070000
H      0.0000000000   -0.0000000000   -2.4924950000
H      0.0000000000    2.3696970000    2.5868070000
H      0.0000000000   -2.3696970000   -2.5868070000
H      0.0000000000    2.3696970000   -2.5868070000
```

The RKS/DFT-optimized geometry of molecule **L3K** is given below (BLYP/cc-pVTZ level of theory). Total electronic energy: -1297.71376628 *a.u.*

```
40

C      0.0000000000   -0.0000000000    2.9040620000
C      0.0000000000   -0.0000000000    1.4521400000
C      0.0000000000    1.2531880000    0.7417440000
C      0.0000000000    2.4414850000    1.4491320000
C      0.0000000000    2.5164960000    2.9051860000
C      0.0000000000   -0.0000000000    5.7354630000
C     -0.0000000000   -1.2216200000    3.6340660000
H      0.0000000000    3.4059600000    0.9540030000
C      0.0000000000    1.2094020000    5.0346240000
C      0.0000000000    1.2216200000    3.6340660000
C     -0.0000000000   -1.2531880000    0.7417440000
```

C	-0.0000000000	-1.2531880000	-0.7417440000
C	-0.0000000000	-2.4414850000	-1.4491320000
C	-0.0000000000	-2.5164960000	-2.9051860000
H	0.0000000000	3.4059600000	-0.9540030000
H	0.0000000000	-0.0000000000	6.8231760000
C	-0.0000000000	-1.2094020000	5.0346240000
C	-0.0000000000	-1.2094020000	-5.0346240000
C	0.0000000000	-1.2216200000	-3.6340660000
C	0.0000000000	-0.0000000000	-2.9040620000
C	0.0000000000	-0.0000000000	-1.4521400000
C	0.0000000000	1.2531880000	-0.7417440000
C	0.0000000000	2.4414850000	-1.4491320000
C	0.0000000000	2.5164960000	-2.9051860000
H	0.0000000000	-0.0000000000	-6.8231760000
H	-0.0000000000	-3.4059600000	-0.9540030000
H	-0.0000000000	-3.4059600000	0.9540030000
C	0.0000000000	1.2094020000	-5.0346240000
C	0.0000000000	-0.0000000000	-5.7354630000
C	0.0000000000	1.2216200000	-3.6340660000
C	-0.0000000000	-2.4414850000	1.4491320000
C	-0.0000000000	-2.5164960000	2.9051860000
O	0.0000000000	3.6141290000	3.4917700000
H	0.0000000000	2.1654580000	5.5524130000
O	-0.0000000000	-3.6141290000	-3.4917700000
H	-0.0000000000	-2.1654580000	5.5524130000
H	-0.0000000000	-2.1654580000	-5.5524130000
O	0.0000000000	3.6141290000	-3.4917700000
H	0.0000000000	2.1654580000	-5.5524130000
O	-0.0000000000	-3.6141290000	3.4917700000

The triplet UKS/DFT-optimized geometry of molecule **L3K** is given below (BLYP/cc-pVTZ level of theory). Total electronic energy: -1297.71310874 $a.u.$

40

C	0.0000000000	-0.0000000000	2.9109550000
C	0.0000000000	-0.0000000000	1.4604350000
C	0.0000000000	1.2520990000	0.7449440000
C	0.0000000000	2.4381980000	1.4585420000
C	0.0000000000	2.5128860000	2.9130910000
C	0.0000000000	-0.0000000000	5.7439950000
C	0.0000000000	-1.2217740000	3.6417750000
H	0.0000000000	3.4051350000	0.9661660000
C	0.0000000000	1.2090130000	5.0429470000
C	0.0000000000	1.2217740000	3.6417750000
C	-0.0000000000	-1.2520990000	0.7449440000
C	-0.0000000000	-1.2520990000	-0.7449440000
C	-0.0000000000	-2.4381980000	-1.4585420000
C	-0.0000000000	-2.5128860000	-2.9130910000
H	0.0000000000	3.4051350000	-0.9661660000
H	0.0000000000	-0.0000000000	6.8317260000
C	-0.0000000000	-1.2090130000	5.0429470000
C	-0.0000000000	-1.2090130000	-5.0429470000
C	0.0000000000	-1.2217740000	-3.6417750000
C	0.0000000000	-0.0000000000	-2.9109550000
C	0.0000000000	-0.0000000000	-1.4604350000
C	0.0000000000	1.2520990000	-0.7449440000
C	0.0000000000	2.4381980000	-1.4585420000

C	0.0000000000	2.5128860000	-2.9130910000
H	0.0000000000	-0.0000000000	-6.8317260000
H	-0.0000000000	-3.4051350000	-0.9661660000
H	-0.0000000000	-3.4051350000	0.9661660000
C	0.0000000000	1.2090130000	-5.0429470000
C	0.0000000000	-0.0000000000	-5.7439950000
C	0.0000000000	1.2217740000	-3.6417750000
C	-0.0000000000	-2.4381980000	1.4585420000
C	-0.0000000000	-2.5128860000	2.9130910000
O	0.0000000000	3.6154560000	3.4957370000
H	0.0000000000	2.1648560000	5.5611520000
O	-0.0000000000	-3.6154560000	-3.4957370000
H	-0.0000000000	-2.1648560000	5.5611520000
H	-0.0000000000	-2.1648560000	-5.5611520000
O	0.0000000000	3.6154560000	-3.4957370000
H	0.0000000000	2.1648560000	-5.5611520000
O	-0.0000000000	-3.6154560000	3.4957370000

The RKS/DFT-optimized geometry of molecule **CG** is given below (BLYP/cc-pVTZ level of theory). Total electronic energy: -1458.86125608 $a.u.$

56

C	0.0000000000	-0.0000000000	2.8854210000
C	0.0000000000	-0.0000000000	1.4441180000
C	0.0000000000	1.2544810000	0.7398430000
C	0.0000000000	2.4359870000	1.4720930000
C	0.0000000000	2.4682720000	2.8950710000
C	0.0000000000	3.6843500000	3.6220840000
C	0.0000000000	3.6813550000	5.0220520000
C	0.0000000000	2.4839660000	5.7312770000
C	0.0000000000	1.2316980000	5.0509210000
C	0.0000000000	1.2299680000	3.6090780000
C	-0.0000000000	-1.2544810000	0.7398430000
C	-0.0000000000	-1.2544810000	-0.7398430000
C	-0.0000000000	-2.4359870000	-1.4720930000
C	-0.0000000000	-2.4682720000	-2.8950710000
C	-0.0000000000	-3.6843500000	-3.6220840000
C	-0.0000000000	-3.6813550000	-5.0220520000
C	-0.0000000000	-2.4839660000	-5.7312770000
C	-0.0000000000	-1.2316980000	-5.0509210000
C	-0.0000000000	-1.2299680000	-3.6090780000
C	0.0000000000	-0.0000000000	-2.8854210000
C	0.0000000000	-0.0000000000	-1.4441180000
C	0.0000000000	1.2544810000	-0.7398430000
C	0.0000000000	2.4359870000	-1.4720930000
C	0.0000000000	2.4682720000	-2.8950710000
C	0.0000000000	3.6843500000	-3.6220840000
C	0.0000000000	3.6813550000	-5.0220520000
C	0.0000000000	2.4839660000	-5.7312770000
C	0.0000000000	1.2316980000	-5.0509210000
C	0.0000000000	0.0000000000	-5.7375620000
C	0.0000000000	1.2299680000	-3.6090780000
C	-0.0000000000	-2.4359870000	1.4720930000
C	-0.0000000000	-2.4682720000	2.8950710000
C	-0.0000000000	-3.6843500000	3.6220840000
C	-0.0000000000	-3.6813550000	5.0220520000
C	-0.0000000000	-2.4839660000	5.7312770000

```
C     -0.0000000000   -1.2316980000    5.0509210000
C     -0.0000000000    0.0000000000    5.7375620000
C     -0.0000000000   -1.2299680000    3.6090780000
H      0.0000000000    3.3952230000    0.9627970000
H      0.0000000000    4.6271490000    3.0772410000
H      0.0000000000    4.6270780000    5.5614120000
H      0.0000000000    2.4908470000    6.8200310000
H     -0.0000000000   -3.3952230000   -0.9627970000
H     -0.0000000000   -4.6271490000   -3.0772410000
H     -0.0000000000   -4.6270780000   -5.5614120000
H     -0.0000000000   -2.4908470000   -6.8200310000
H      0.0000000000    3.3952230000   -0.9627970000
H      0.0000000000    4.6271490000   -3.0772410000
H      0.0000000000    4.6270780000   -5.5614120000
H      0.0000000000    2.4908470000   -6.8200310000
H     -0.0000000000   -3.3952230000    0.9627970000
H     -0.0000000000   -4.6271490000    3.0772410000
H     -0.0000000000   -4.6270780000    5.5614120000
H     -0.0000000000   -2.4908470000    6.8200310000
H      0.0000000000    0.0000000000   -6.8271760000
H     -0.0000000000    0.0000000000    6.8271760000
```

The triplet UKS/DFT-optimized geometry of molecule **CG** is given below (BLYP/cc-pVTZ level of theory). Total electronic energy: -1458.86630198 $a.u.$

```
56

C      0.0000000000   -0.0000000000    2.8854050000
C      0.0000000000   -0.0000000000    1.4476390000
C      0.0000000000    1.2547110000    0.7410020000
C      0.0000000000    2.4354830000    1.4755800000
C      0.0000000000    2.4675420000    2.8959830000
C      0.0000000000    3.6882450000    3.6238230000
C      0.0000000000    3.6849150000    5.0204600000
C      0.0000000000    2.4840840000    5.7303680000
C      0.0000000000    1.2336380000    5.0512440000
C      0.0000000000    1.2314040000    3.6100450000
C     -0.0000000000   -1.2547110000    0.7410020000
C     -0.0000000000   -1.2547110000   -0.7410020000
C     -0.0000000000   -2.4354830000   -1.4755800000
C     -0.0000000000   -2.4675420000   -2.8959830000
C     -0.0000000000   -3.6882450000   -3.6238230000
C     -0.0000000000   -3.6849150000   -5.0204600000
C     -0.0000000000   -2.4840840000   -5.7303680000
C     -0.0000000000   -1.2336380000   -5.0512440000
C     -0.0000000000   -1.2314040000   -3.6100450000
C      0.0000000000   -0.0000000000   -2.8854050000
C      0.0000000000   -0.0000000000   -1.4476390000
C      0.0000000000    1.2547110000   -0.7410020000
C      0.0000000000    2.4354830000   -1.4755800000
C      0.0000000000    2.4675420000   -2.8959830000
C      0.0000000000    3.6882450000   -3.6238230000
C      0.0000000000    3.6849150000   -5.0204600000
C      0.0000000000    2.4840840000   -5.7303680000
C      0.0000000000    1.2336380000   -5.0512440000
C      0.0000000000    0.0000000000   -5.7391030000
C      0.0000000000    1.2314040000   -3.6100450000
C     -0.0000000000   -2.4354830000    1.4755800000
```

C	-0.0000000000	-2.4675420000	2.8959830000
C	-0.0000000000	-3.6882450000	3.6238230000
C	-0.0000000000	-3.6849150000	5.0204600000
C	-0.0000000000	-2.4840840000	5.7303680000
C	-0.0000000000	-1.2336380000	5.0512440000
C	-0.0000000000	0.0000000000	5.7391030000
C	-0.0000000000	-1.2314040000	3.6100450000
H	0.0000000000	3.3952920000	0.9667810000
H	0.0000000000	4.6301420000	3.0774930000
H	0.0000000000	4.6298260000	5.5611520000
H	0.0000000000	2.4913280000	6.8191530000
H	-0.0000000000	-3.3952920000	-0.9667810000
H	-0.0000000000	-4.6301420000	-3.0774930000
H	-0.0000000000	-4.6298260000	-5.5611520000
H	-0.0000000000	-2.4913280000	-6.8191530000
H	0.0000000000	3.3952920000	-0.9667810000
H	0.0000000000	4.6301420000	-3.0774930000
H	0.0000000000	4.6298260000	-5.5611520000
H	0.0000000000	2.4913280000	-6.8191530000
H	-0.0000000000	-3.3952920000	0.9667810000
H	-0.0000000000	-4.6301420000	3.0774930000
H	-0.0000000000	-4.6298260000	5.5611520000
H	-0.0000000000	-2.4913280000	6.8191530000
H	0.0000000000	0.0000000000	-6.8286820000
H	-0.0000000000	0.0000000000	6.8286820000

The RKS/DFT-optimized geometry of molecule **PA** is given below (BLYP/cc-pVTZ level of theory). Total electronic energy: −1144.28663756 *a.u.*

46

H	-0.0000000000	3.2124860000	3.2186200000
N	0.0000000000	-2.1457320000	0.0000000000
N	-0.0000000000	0.0000000000	2.1017740000
N	0.0000000000	2.1457320000	0.0000000000
N	0.0000000000	0.0000000000	-2.1017740000
C	-0.0000000000	-2.9371150000	1.1133880000
C	0.0000000000	-2.9371150000	-1.1133880000
C	0.0000000000	2.9371150000	1.1133880000
C	0.0000000000	2.9371150000	-1.1133880000
C	-0.0000000000	-1.1397420000	2.8858580000
C	0.0000000000	1.1397420000	2.8858580000
C	0.0000000000	-1.1397420000	-2.8858580000
C	0.0000000000	1.1397420000	-2.8858580000
C	-0.0000000000	-4.3801920000	0.7433530000
C	0.0000000000	-4.3801920000	-0.7433530000
C	0.0000000000	4.3801920000	0.7433530000
C	0.0000000000	4.3801920000	-0.7433530000
C	-0.0000000000	-0.6897750000	4.2497510000
C	0.0000000000	0.6897750000	4.2497510000
C	0.0000000000	-0.6897750000	-4.2497510000
C	0.0000000000	0.6897750000	-4.2497510000
C	-0.0000000000	-2.4638110000	2.4300910000
C	0.0000000000	-2.4638110000	-2.4300910000
C	0.0000000000	2.4638110000	-2.4300910000
C	0.0000000000	2.4638110000	2.4300910000
C	-0.0000000000	-5.4452600000	1.5680820000
C	0.0000000000	-5.4452600000	-1.5680820000

```
C      0.0000000000    5.4452600000    1.5680820000
C      0.0000000000    5.4452600000   -1.5680820000
H     -0.0000000000   -3.2124860000   -3.2186200000
H      0.0000000000    3.2124860000   -3.2186200000
H      0.0000000000   -0.0000000000   -1.0873640000
H     -0.0000000000   -0.0000000000    1.0873640000
H     -0.0000000000   -3.2124860000    3.2186200000
H     -0.0000000000   -1.3492650000    5.1090600000
H      0.0000000000    1.3492650000    5.1090600000
H      0.0000000000   -1.3492650000   -5.1090600000
H      0.0000000000    1.3492650000   -5.1090600000
H     -0.0000000000   -6.4634370000    1.1856470000
H     -0.0000000000   -5.3375650000    2.6494820000
H      0.0000000000   -5.3375650000   -2.6494820000
H      0.0000000000   -6.4634370000   -1.1856470000
H      0.0000000000    6.4634370000    1.1856470000
H      0.0000000000    5.3375650000    2.6494820000
H      0.0000000000    5.3375650000   -2.6494820000
H      0.0000000000    6.4634370000   -1.1856470000
```

The triplet UKS/DFT-optimized geometry of molecule **PA** is given below (BLYP/cc-pVTZ level of theory). Total electronic energy: −1144.24237653 *a.u.*

```
46

H     -0.0000000000    3.2220820000    3.2195230000
N      0.0000000000   -2.1099650000    0.0000000000
N     -0.0000000000    0.0000000000    2.1141850000
N      0.0000000000    2.1099650000    0.0000000000
N      0.0000000000    0.0000000000   -2.1141850000
C     -0.0000000000   -2.9183180000    1.1149070000
C      0.0000000000   -2.9183180000   -1.1149070000
C      0.0000000000    2.9183180000    1.1149070000
C      0.0000000000    2.9183180000   -1.1149070000
C     -0.0000000000   -1.1394620000    2.8992340000
C      0.0000000000    1.1394620000    2.8992340000
C      0.0000000000   -1.1394620000   -2.8992340000
C      0.0000000000    1.1394620000   -2.8992340000
C     -0.0000000000   -4.3535500000    0.7411820000
C      0.0000000000   -4.3535500000   -0.7411820000
C      0.0000000000    4.3535500000    0.7411820000
C      0.0000000000    4.3535500000   -0.7411820000
C     -0.0000000000   -0.6909370000    4.2641220000
C      0.0000000000    0.6909370000    4.2641220000
C      0.0000000000   -0.6909370000   -4.2641220000
C      0.0000000000    0.6909370000   -4.2641220000
C     -0.0000000000   -2.4672950000    2.4379520000
C      0.0000000000   -2.4672950000   -2.4379520000
C      0.0000000000    2.4672950000   -2.4379520000
C      0.0000000000    2.4672950000    2.4379520000
C     -0.0000000000   -5.4291990000    1.5586610000
C      0.0000000000   -5.4291990000   -1.5586610000
C      0.0000000000    5.4291990000    1.5586610000
C      0.0000000000    5.4291990000   -1.5586610000
H     -0.0000000000   -3.2220820000   -3.2195230000
H      0.0000000000    3.2220820000   -3.2195230000
H      0.0000000000   -0.0000000000   -1.0982050000
H     -0.0000000000   -0.0000000000    1.0982050000
```

```
H     -0.0000000000   -3.2220820000    3.2195230000
H     -0.0000000000   -1.3512270000    5.1226450000
H      0.0000000000    1.3512270000    5.1226450000
H      0.0000000000   -1.3512270000   -5.1226450000
H      0.0000000000    1.3512270000   -5.1226450000
H     -0.0000000000   -6.4432030000    1.1657120000
H     -0.0000000000   -5.3314830000    2.6407800000
H      0.0000000000   -5.3314830000   -2.6407800000
H      0.0000000000   -6.4432030000   -1.1657120000
H      0.0000000000    6.4432030000    1.1657120000
H      0.0000000000    5.3314830000    2.6407800000
H      0.0000000000    5.3314830000   -2.6407800000
H      0.0000000000    6.4432030000   -1.1657120000
```

The RKS/DFT-optimized geometry of molecule **PT** is given below (BLYP/cc-pVTZ level of theory).
Total electronic energy: -1144.26504764 *a.u.*

```
46

H     -0.0000000000    1.1347680000   -0.0000000000
N      0.4917500000    2.0346850000    0.0000000000
N     -2.0159320000    0.8265590000   -0.0000000000
N     -0.4693710000   -1.9658520000   -0.0000000000
N      2.0447820000   -0.8605660000    0.0000000000
C     -0.2652120000    3.1990020000   -0.0000000000
C      1.8427290000    2.3352150000    0.0000000000
C     -1.8057540000   -2.3011060000   -0.0000000000
C      0.2134530000   -3.1523050000    0.0000000000
C     -2.5046840000    2.1060900000   -0.0000000000
C     -3.0432770000   -0.0537590000   -0.0000000000
C      3.0570380000    0.0781800000    0.0000000000
C      2.4879660000   -2.1656670000    0.0000000000
C      0.6554190000    4.2897510000    0.0000000000
C      1.9360230000    3.7643820000    0.0000000000
C     -1.9579090000   -3.7604300000   -0.0000000000
C     -0.7024470000   -4.2891880000   -0.0000000000
C     -3.9902130000    2.1080430000   -0.0000000000
C     -4.3548240000    0.6641570000   -0.0000000000
C      4.3335940000   -0.6836890000    0.0000000000
C      3.9608150000   -2.1317670000    0.0000000000
C     -1.6708250000    3.2245030000   -0.0000000000
C      2.9481550000    1.4573500000    0.0000000000
C      1.6238830000   -3.2484660000    0.0000000000
C     -2.9227360000   -1.4547640000   -0.0000000000
H      0.3718110000    5.3349980000    0.0000000000
H      2.8713250000    4.3108490000    0.0000000000
H     -2.9057560000   -4.2880160000   -0.0000000000
H     -0.4130770000   -5.3345340000   -0.0000000000
C     -4.8147090000    3.1733110000   -0.0000000000
C     -5.5913770000    0.1304390000   -0.0000000000
C      5.5803750000   -0.1707180000    0.0000000000
C      4.7875060000   -3.1974960000    0.0000000000
H      1.0123120000   -0.7391860000    0.0000000000
H     -2.1294700000    4.2101430000   -0.0000000000
H      3.9008610000    1.9812830000    0.0000000000
H      2.0606180000   -4.2434710000    0.0000000000
H     -3.8705620000   -1.9907180000   -0.0000000000
H     -5.8965240000    3.0607580000   -0.0000000000
```

H	-4.4355610000	4.1921370000	-0.0000000000
H	-5.7569440000	-0.9434780000	-0.0000000000
H	-6.4819540000	0.7550760000	-0.0000000000
H	6.4571250000	-0.8125370000	0.0000000000
H	5.7656840000	0.8994620000	0.0000000000
H	4.4054990000	-4.2144730000	0.0000000000
H	5.8680720000	-3.0832540000	0.0000000000

The triplet UKS/DFT-optimized geometry of molecule **PT** is given below (B3LYP/cc-pVTZ level of theory). Total electronic energy: -1144.22801090 *a.u.*

46

H	0.0000000000	1.1406710000	-0.0000000000
N	0.5012100000	2.0374040000	-0.0000000000
N	-1.9885970000	0.8290110000	0.0000000000
N	-0.4955680000	-1.9723820000	0.0000000000
N	2.0092730000	-0.8475200000	-0.0000000000
C	-0.2516180000	3.2056290000	-0.0000000000
C	1.8548980000	2.3318790000	-0.0000000000
C	-1.8184200000	-2.3108210000	0.0000000000
C	0.1989950000	-3.1629950000	0.0000000000
C	-2.4756750000	2.0928640000	0.0000000000
C	-3.0465190000	-0.0640570000	0.0000000000
C	3.0488170000	0.0707840000	-0.0000000000
C	2.4711300000	-2.1671200000	-0.0000000000
C	0.6816640000	4.2986610000	-0.0000000000
C	1.9538140000	3.7676000000	-0.0000000000
C	-1.9721960000	-3.7712970000	0.0000000000
C	-0.7143630000	-4.2999410000	0.0000000000
C	-3.9592470000	2.1091730000	0.0000000000
C	-4.3403550000	0.6726710000	0.0000000000
C	4.3067250000	-0.6961620000	-0.0000000000
C	3.9285790000	-2.1338040000	-0.0000000000
C	-1.6491990000	3.2380080000	0.0000000000
C	2.9590260000	1.4554830000	-0.0000000000
C	1.6104740000	-3.2523270000	-0.0000000000
C	-2.9460130000	-1.4485660000	0.0000000000
H	0.4016520000	5.3447080000	-0.0000000000
H	2.8930480000	4.3072260000	-0.0000000000
H	-2.9193840000	-4.3001200000	0.0000000000
H	-0.4238520000	-5.3447630000	0.0000000000
C	-4.7691690000	3.1865620000	0.0000000000
C	-5.5916770000	0.1679470000	0.0000000000
C	5.5703850000	-0.1967240000	-0.0000000000
C	4.7734940000	-3.2008930000	-0.0000000000
H	0.9846980000	-0.7183760000	-0.0000000000
H	-2.1156120000	4.2184320000	0.0000000000
H	3.9146780000	1.9728570000	-0.0000000000
H	2.0504670000	-4.2459890000	-0.0000000000
H	-3.8965440000	-1.9793790000	0.0000000000
H	-5.8525290000	3.0889350000	0.0000000000
H	-4.3756400000	4.1996030000	0.0000000000
H	-5.7822040000	-0.9017840000	0.0000000000
H	-6.4665470000	0.8141690000	0.0000000000
H	6.4353510000	-0.8532190000	-0.0000000000
H	5.7721400000	0.8702340000	-0.0000000000
H	4.4030730000	-4.2218550000	-0.0000000000

H	5.8516820000	-3.0722610000	-0.0000000000

The RKS/DFT-optimized geometry of molecule **PB** is given below (BLYP/cc-pVTZ level of theory).
Total electronic energy: -1144.26451573 *a.u.*

46

H	0.0000000000	3.2453000000	3.1753050000
N	0.0000000000	-2.0129420000	0.0000000000
N	0.0000000000	0.0000000000	2.2104500000
N	0.0000000000	2.0129420000	0.0000000000
N	0.0000000000	0.0000000000	-2.2104500000
C	0.0000000000	-2.8466630000	1.0921530000
C	0.0000000000	-2.8466630000	-1.0921530000
C	0.0000000000	2.8466630000	1.0921530000
C	0.0000000000	2.8466630000	-1.0921530000
C	0.0000000000	-1.1553060000	2.9569800000
C	0.0000000000	1.1553060000	2.9569800000
C	0.0000000000	-1.1553060000	-2.9569800000
C	0.0000000000	1.1553060000	-2.9569800000
C	0.0000000000	-4.2466220000	0.6823880000
C	0.0000000000	-4.2466220000	-0.6823880000
C	0.0000000000	4.2466220000	0.6823880000
C	0.0000000000	4.2466220000	-0.6823880000
C	0.0000000000	-0.7475810000	4.3783920000
C	0.0000000000	0.7475810000	4.3783920000
C	0.0000000000	-0.7475810000	-4.3783920000
C	0.0000000000	0.7475810000	-4.3783920000
C	0.0000000000	-2.4418700000	2.4422900000
C	0.0000000000	-2.4418700000	-2.4422900000
C	0.0000000000	2.4418700000	-2.4422900000
C	0.0000000000	2.4418700000	2.4422900000
H	0.0000000000	-5.0991850000	1.3529890000
H	0.0000000000	-5.0991850000	-1.3529890000
H	0.0000000000	5.0991850000	1.3529890000
H	0.0000000000	5.0991850000	-1.3529890000
C	0.0000000000	-1.5671750000	5.4494480000
C	0.0000000000	1.5671750000	5.4494480000
C	0.0000000000	-1.5671750000	-5.4494480000
C	0.0000000000	1.5671750000	-5.4494480000
H	-0.0000000000	-3.2453000000	3.1753050000
H	-0.0000000000	-3.2453000000	-3.1753050000
H	0.0000000000	3.2453000000	-3.1753050000
H	0.0000000000	0.0000000000	-1.1849380000
H	-0.0000000000	0.0000000000	1.1849380000
H	0.0000000000	-2.6482130000	5.3437140000
H	0.0000000000	-1.1782990000	6.4640510000
H	0.0000000000	2.6482130000	5.3437140000
H	0.0000000000	1.1782990000	6.4640510000
H	0.0000000000	-1.1782990000	-6.4640510000
H	0.0000000000	-2.6482130000	-5.3437140000
H	0.0000000000	1.1782990000	-6.4640510000
H	0.0000000000	2.6482130000	-5.3437140000

The triplet UKS/DFT-optimized geometry of molecule **PB** is given below (BLYP/cc-pVTZ level of theory).
Total electronic energy: -1144.23465868 *a.u.*

46

H	0.0000000000	3.2460870000	3.1827150000
N	0.0000000000	-2.0197820000	0.0000000000
N	0.0000000000	0.0000000000	2.1808790000
N	0.0000000000	2.0197820000	-0.0000000000
N	0.0000000000	-0.0000000000	-2.1808790000
C	0.0000000000	-2.8520490000	1.0923660000
C	0.0000000000	-2.8520490000	-1.0923660000
C	0.0000000000	2.8520490000	1.0923660000
C	0.0000000000	2.8520490000	-1.0923660000
C	0.0000000000	-1.1564660000	2.9484090000
C	0.0000000000	1.1564660000	2.9484090000
C	0.0000000000	-1.1564660000	-2.9484090000
C	0.0000000000	1.1564660000	-2.9484090000
C	0.0000000000	-4.2555530000	0.6818390000
C	0.0000000000	-4.2555530000	-0.6818390000
C	0.0000000000	4.2555530000	0.6818390000
C	0.0000000000	4.2555530000	-0.6818390000
C	0.0000000000	-0.7449070000	4.3600670000
C	0.0000000000	0.7449070000	4.3600670000
C	0.0000000000	-0.7449070000	-4.3600670000
C	0.0000000000	0.7449070000	-4.3600670000
C	0.0000000000	-2.4453420000	2.4473940000
C	0.0000000000	-2.4453420000	-2.4473940000
C	0.0000000000	2.4453420000	-2.4473940000
C	0.0000000000	2.4453420000	2.4473940000
H	0.0000000000	-5.1075120000	1.3530530000
H	0.0000000000	-5.1075120000	-1.3530530000
H	0.0000000000	5.1075120000	1.3530530000
H	0.0000000000	5.1075120000	-1.3530530000
C	0.0000000000	-1.5569820000	5.4440510000
C	0.0000000000	1.5569820000	5.4440510000
C	0.0000000000	-1.5569820000	-5.4440510000
C	0.0000000000	1.5569820000	-5.4440510000
H	-0.0000000000	-3.2460870000	3.1827150000
H	-0.0000000000	-3.2460870000	-3.1827150000
H	0.0000000000	3.2460870000	-3.1827150000
H	-0.0000000000	0.0000000000	-1.1598390000
H	-0.0000000000	0.0000000000	1.1598390000
H	0.0000000000	-2.6389880000	5.3503820000
H	0.0000000000	-1.1556720000	6.4533480000
H	0.0000000000	2.6389880000	5.3503820000
H	0.0000000000	1.1556720000	6.4533480000
H	0.0000000000	-1.1556720000	-6.4533480000
H	0.0000000000	-2.6389880000	-5.3503820000
H	0.0000000000	1.1556720000	-6.4533480000
H	0.0000000000	2.6389880000	-5.3503820000

The quintet UKS/DFT-optimized geometry of molecule **PB** is given below (BLYP/cc-pVTZ level of theory). Total electronic energy: -1144.16363540 *a.u.*

46

H	0.0000000000	3.2422820000	3.1846810000
N	0.0000000000	-2.0305490000	0.0000000000
N	0.0000000000	0.0000000000	2.1487440000
N	0.0000000000	2.0305490000	0.0000000000
N	0.0000000000	-0.0000000000	-2.1487440000

C	0.0000000000	-2.8631920000	1.0949150000
C	0.0000000000	-2.8631920000	-1.0949150000
C	0.0000000000	2.8631920000	1.0949150000
C	0.0000000000	2.8631920000	-1.0949150000
C	0.0000000000	-1.1439520000	2.9346820000
C	0.0000000000	1.1439520000	2.9346820000
C	0.0000000000	-1.1439520000	-2.9346820000
C	0.0000000000	1.1439520000	-2.9346820000
C	0.0000000000	-4.2661980000	0.6812800000
C	0.0000000000	-4.2661980000	-0.6812800000
C	0.0000000000	4.2661980000	0.6812800000
C	0.0000000000	4.2661980000	-0.6812800000
C	0.0000000000	-0.7224950000	4.3200040000
C	0.0000000000	0.7224950000	4.3200040000
C	0.0000000000	-0.7224950000	-4.3200040000
C	0.0000000000	0.7224950000	-4.3200040000
C	0.0000000000	-2.4499610000	2.4405660000
C	0.0000000000	-2.4499610000	-2.4405660000
C	0.0000000000	2.4499610000	-2.4405660000
C	0.0000000000	2.4499610000	2.4405660000
H	0.0000000000	-5.1188330000	1.3516570000
H	0.0000000000	-5.1188330000	-1.3516570000
H	0.0000000000	5.1188330000	1.3516570000
H	0.0000000000	5.1188330000	-1.3516570000
C	0.0000000000	-1.5551440000	5.4488490000
C	0.0000000000	1.5551440000	5.4488490000
C	0.0000000000	-1.5551440000	-5.4488490000
C	0.0000000000	1.5551440000	-5.4488490000
H	-0.0000000000	-3.2422820000	3.1846810000
H	-0.0000000000	-3.2422820000	-3.1846810000
H	0.0000000000	3.2422820000	-3.1846810000
H	0.0000000000	0.0000000000	-1.1304420000
H	-0.0000000000	0.0000000000	1.1304420000
H	0.0000000000	-2.6365140000	5.3610970000
H	0.0000000000	-1.1397260000	6.4506810000
H	0.0000000000	2.6365140000	5.3610970000
H	0.0000000000	1.1397260000	6.4506810000
H	0.0000000000	-1.1397260000	-6.4506810000
H	0.0000000000	-2.6365140000	-5.3610970000
H	0.0000000000	1.1397260000	-6.4506810000
H	0.0000000000	2.6365140000	-5.3610970000

The triplet UKS/DFT-optimized geometry of molecule **PE-Z** is given below (BLYP/cc-pVTZ level of theory). Total electronic energy: -1448.86542265 $a.u.$

54

H	-0.0000000000	-0.0000000000	1.1289990000
N	0.0000000000	2.0817190000	0.0000000000
N	-0.0000000000	0.0000000000	2.1467900000
N	-0.0000000000	-2.0817190000	0.0000000000
N	0.0000000000	-0.0000000000	-2.1467900000
C	0.0000000000	2.9169920000	1.0883260000
C	0.0000000000	2.9169920000	-1.0883260000
C	-0.0000000000	-2.9169920000	1.0883260000
C	-0.0000000000	-2.9169920000	-1.0883260000
C	0.0000000000	1.1307480000	2.9393560000
C	-0.0000000000	-1.1307480000	2.9393560000

```
C      0.0000000000    1.1307480000   -2.9393560000
C     -0.0000000000   -1.1307480000   -2.9393560000
C      0.0000000000    4.3149680000    0.6807680000
C      0.0000000000    4.3149680000   -0.6807680000
C     -0.0000000000   -4.3149680000    0.6807680000
C     -0.0000000000   -4.3149680000   -0.6807680000
C      0.0000000000    0.6960700000    4.2844610000
C     -0.0000000000   -0.6960700000    4.2844610000
C      0.0000000000    0.6960700000   -4.2844610000
C     -0.0000000000   -0.6960700000   -4.2844610000
C      0.0000000000    2.5018750000    2.4683420000
C      0.0000000000    2.5018750000   -2.4683420000
C     -0.0000000000   -2.5018750000   -2.4683420000
C     -0.0000000000   -2.5018750000    2.4683420000
H      0.0000000000    5.1661780000    1.3485420000
H      0.0000000000    5.1661780000   -1.3485420000
H     -0.0000000000   -5.1661780000    1.3485420000
H     -0.0000000000   -5.1661780000   -1.3485420000
H      0.0000000000    1.3515800000    5.1442560000
H     -0.0000000000   -1.3515800000    5.1442560000
H      0.0000000000    1.3515800000   -5.1442560000
H     -0.0000000000   -1.3515800000   -5.1442560000
C      0.0000000000   -5.3305460000   -5.2524380000
C     -0.0000000000   -5.3305460000    5.2524380000
C      0.0000000000    5.3305460000   -5.2524380000
H      0.0000000000   -0.0000000000   -1.1289990000
C      0.0000000000    5.3305460000    5.2524380000
C      0.0000000000    3.4899550000    3.4288420000
C      0.0000000000    4.3760460000    4.3115050000
C      0.0000000000    3.4899550000   -3.4288420000
C      0.0000000000    4.3760460000   -4.3115050000
C     -0.0000000000   -3.4899550000    3.4288420000
C     -0.0000000000   -4.3760460000    4.3115050000
C     -0.0000000000   -3.4899550000   -3.4288420000
C     -0.0000000000   -4.3760460000   -4.3115050000
H      0.0000000000   -5.0811720000   -6.3120580000
H      0.0000000000   -6.3865770000   -4.9880420000
H     -0.0000000000   -5.0811720000    6.3120580000
H     -0.0000000000   -6.3865770000    4.9880420000
H      0.0000000000    6.3865770000   -4.9880420000
H      0.0000000000    5.0811720000   -6.3120580000
H      0.0000000000    6.3865770000    4.9880420000
H      0.0000000000    5.0811720000    6.3120580000
```

The quintet UKS/DFT-optimized geometry of molecule **PE-Z** is given below (BLYP/cc-pVTZ level of theory). Total electronic energy: -1448.83317624 $a.u.$

```
54

H      0.0000000000   -0.0000000000    1.1142320000
N      0.0000000000    2.0627780000    0.0000000000
N     -0.0000000000   -0.0000000000    2.1327430000
N     -0.0000000000   -2.0627780000    0.0000000000
N      0.0000000000   -0.0000000000   -2.1327430000
C     -0.0000000000    2.8988690000    1.0896120000
C      0.0000000000    2.8988690000   -1.0896120000
C     -0.0000000000   -2.8988690000    1.0896120000
C     -0.0000000000   -2.8988690000   -1.0896120000
```

C	-0.0000000000	1.1328690000	2.9240100000
C	-0.0000000000	-1.1328690000	2.9240100000
C	0.0000000000	1.1328690000	-2.9240100000
C	-0.0000000000	-1.1328690000	-2.9240100000
C	-0.0000000000	4.2979470000	0.6807000000
C	0.0000000000	4.2979470000	-0.6807000000
C	-0.0000000000	-4.2979470000	0.6807000000
C	-0.0000000000	-4.2979470000	-0.6807000000
C	-0.0000000000	0.6896250000	4.2832880000
C	-0.0000000000	-0.6896250000	4.2832880000
C	0.0000000000	0.6896250000	-4.2832880000
C	-0.0000000000	-0.6896250000	-4.2832880000
C	-0.0000000000	2.4860880000	2.4627410000
C	0.0000000000	2.4860880000	-2.4627410000
C	-0.0000000000	-2.4860880000	-2.4627410000
C	-0.0000000000	-2.4860880000	2.4627410000
H	-0.0000000000	5.1490140000	1.3489150000
H	0.0000000000	5.1490140000	-1.3489150000
H	-0.0000000000	-5.1490140000	1.3489150000
H	-0.0000000000	-5.1490140000	-1.3489150000
H	-0.0000000000	1.3492390000	5.1398220000
H	-0.0000000000	-1.3492390000	5.1398220000
H	0.0000000000	1.3492390000	-5.1398220000
H	-0.0000000000	-1.3492390000	-5.1398220000
C	0.0000000000	-5.3199510000	-5.2859080000
C	-0.0000000000	-5.3199510000	5.2859080000
C	0.0000000000	5.3199510000	-5.2859080000
H	0.0000000000	-0.0000000000	-1.1142320000
C	0.0000000000	5.3199510000	5.2859080000
C	-0.0000000000	3.4825700000	3.4505630000
C	-0.0000000000	4.3593150000	4.3309660000
C	0.0000000000	3.4825700000	-3.4505630000
C	0.0000000000	4.3593150000	-4.3309660000
C	-0.0000000000	-3.4825700000	3.4505630000
C	-0.0000000000	-4.3593150000	4.3309660000
C	-0.0000000000	-3.4825700000	-3.4505630000
C	-0.0000000000	-4.3593150000	-4.3309660000
H	0.0000000000	-5.0648530000	-6.3435900000
H	0.0000000000	-6.3758180000	-5.0232310000
H	-0.0000000000	-5.0648530000	6.3435900000
H	-0.0000000000	-6.3758180000	5.0232310000
H	0.0000000000	6.3758180000	-5.0232310000
H	0.0000000000	5.0648530000	-6.3435900000
H	0.0000000000	6.3758180000	5.0232310000
H	0.0000000000	5.0648530000	6.3435900000

The triplet UKS/DFT-optimized geometry of molecule **PE-T** is given below (BLYP/cc-pVTZ level of theory). Total electronic energy: -1448.85118571 *a.u.*

54

H	0.0000000000	8.1659550000	1.0281450000
N	0.0000000000	1.6125520000	-1.4331240000
N	0.0000000000	1.5648710000	1.3797400000
N	0.0000000000	-1.5648710000	1.3797400000
N	0.0000000000	-1.6125520000	-1.4331240000
C	0.0000000000	2.9882980000	-1.2593230000
C	0.0000000000	1.3106370000	-2.7833010000

C	-0.0000000000	-1.3043940000	2.7176890000
C	-0.0000000000	-2.9347780000	1.2760100000
C	-0.0000000000	2.9347780000	1.2760100000
C	-0.0000000000	1.3043940000	2.7176890000
C	0.0000000000	-1.3106370000	-2.7833010000
C	0.0000000000	-2.9882980000	-1.2593230000
C	0.0000000000	3.5765240000	-2.5416860000
C	0.0000000000	2.5471920000	-3.4761760000
C	-0.0000000000	-2.5469250000	3.4898240000
C	-0.0000000000	-3.5659060000	2.5858910000
C	-0.0000000000	3.5659060000	2.5858910000
C	-0.0000000000	2.5469250000	3.4898240000
C	0.0000000000	-2.5471920000	-3.4761760000
C	0.0000000000	-3.5765240000	-2.5416860000
C	0.0000000000	3.6385080000	0.0270840000
C	-0.0000000000	-0.0000000000	-3.4265080000
C	0.0000000000	-3.6385080000	0.0270840000
C	0.0000000000	0.0000000000	3.3535340000
H	0.0000000000	4.6404730000	-2.7362270000
H	0.0000000000	2.6419820000	-4.5527720000
H	-0.0000000000	-2.6180690000	4.5691710000
H	-0.0000000000	-4.6311990000	2.7773090000
H	-0.0000000000	4.6311990000	2.7773090000
H	-0.0000000000	2.6180690000	4.5691710000
H	0.0000000000	-2.6419820000	-4.5527720000
H	0.0000000000	-4.6404730000	-2.7362270000
C	-0.0000000000	-7.6146760000	0.0894340000
C	0.0000000000	0.0000000000	7.3279380000
C	-0.0000000000	-0.0000000000	-7.3813330000
H	0.0000000000	8.1945880000	-0.8318010000
C	-0.0000000000	7.6146760000	0.0894340000
C	0.0000000000	5.0228880000	0.0544080000
C	0.0000000000	6.2708530000	0.0686060000
C	-0.0000000000	-0.0000000000	-4.7912500000
C	-0.0000000000	-0.0000000000	-6.0461100000
C	0.0000000000	0.0000000000	4.7366530000
C	0.0000000000	0.0000000000	5.9868690000
C	0.0000000000	-5.0228880000	0.0544080000
C	0.0000000000	-6.2708530000	0.0686060000
H	0.0000000000	-8.1945880000	-0.8318010000
H	0.0000000000	-8.1659550000	1.0281450000
H	0.0000000000	0.9295960000	7.8949250000
H	0.0000000000	-0.9295960000	7.8949250000
H	0.0000000000	0.9291590000	-7.9488410000
H	0.0000000000	-0.9291590000	-7.9488410000
H	0.0000000000	1.0181880000	-0.5963600000
H	0.0000000000	-1.0181880000	-0.5963600000

The quintet UKS/DFT-optimized geometry of molecule **PE-T** is given below (BLYP/cc-pVTZ level of theory). Total electronic energy: -1448.82223490 *a.u.*

54

H	0.0000000000	8.1884060000	1.0032560000
N	0.0000000000	1.6090060000	-1.4054630000
N	0.0000000000	1.5662080000	1.3684330000
N	0.0000000000	-1.5662080000	1.3684330000
N	0.0000000000	-1.6090060000	-1.4054630000

C	0.0000000000	2.9857910000	-1.2476310000
C	0.0000000000	1.2886740000	-2.7553050000
C	-0.0000000000	-1.2981090000	2.7089570000
C	-0.0000000000	-2.9345050000	1.2693290000
C	-0.0000000000	2.9345050000	1.2693290000
C	-0.0000000000	1.2981090000	2.7089570000
C	0.0000000000	-1.2886740000	-2.7553050000
C	0.0000000000	-2.9857910000	-1.2476310000
C	0.0000000000	3.5644550000	-2.5472290000
C	0.0000000000	2.5344880000	-3.4655050000
C	-0.0000000000	-2.5418110000	3.4833040000
C	-0.0000000000	-3.5624720000	2.5824210000
C	-0.0000000000	3.5624720000	2.5824210000
C	-0.0000000000	2.5418110000	3.4833040000
C	0.0000000000	-2.5344880000	-3.4655050000
C	0.0000000000	-3.5644550000	-2.5472290000
C	0.0000000000	3.6438340000	0.0225820000
C	0.0000000000	0.0000000000	-3.3723970000
C	0.0000000000	-3.6438340000	0.0225820000
C	-0.0000000000	-0.0000000000	3.3387720000
H	0.0000000000	4.6272160000	-2.7476560000
H	0.0000000000	2.6155520000	-4.5430810000
H	-0.0000000000	-2.6100100000	4.5627770000
H	-0.0000000000	-4.6275140000	2.7753250000
H	-0.0000000000	4.6275140000	2.7753250000
H	-0.0000000000	2.6100100000	4.5627770000
H	0.0000000000	-2.6155520000	-4.5430810000
H	0.0000000000	-4.6272160000	-2.7476560000
C	-0.0000000000	-7.6355210000	0.0660810000
C	-0.0000000000	-0.0000000000	7.3311450000
C	0.0000000000	0.0000000000	-7.3887150000
H	0.0000000000	8.2112800000	-0.8571730000
C	-0.0000000000	7.6355210000	0.0660810000
C	0.0000000000	5.0397930000	0.0396890000
C	0.0000000000	6.2838650000	0.0499990000
C	0.0000000000	0.0000000000	-4.7885270000
C	0.0000000000	0.0000000000	-6.0273000000
C	-0.0000000000	-0.0000000000	4.7366950000
C	-0.0000000000	-0.0000000000	5.9822470000
C	0.0000000000	-5.0397930000	0.0396890000
C	0.0000000000	-6.2838650000	0.0499990000
H	0.0000000000	-8.2112800000	-0.8571730000
H	0.0000000000	-8.1884060000	1.0032560000
H	0.0000000000	0.9298960000	7.8968480000
H	0.0000000000	-0.9298960000	7.8968480000
H	0.0000000000	0.9305260000	-7.9518870000
H	0.0000000000	-0.9305260000	-7.9518870000
H	0.0000000000	1.0290350000	-0.5551020000
H	0.0000000000	-1.0290350000	-0.5551020000

The triplet UKS/DFT-optimized geometry of molecule **QD-Z** is given below (BLYP/cc-pVTZ level of theory). Total electronic energy: −1374.03486577 *a.u.*

54

H	-3.8159180000	5.2639640000	0.8664190000
N	-2.0812100000	-0.0674530000	0.0000000000
N	-0.0540200000	2.1333750000	0.0000000000

N	2.0812100000	0.0674530000	0.0000000000
N	0.0540200000	-2.1333750000	0.0000000000
C	-2.9122760000	0.9584670000	0.0000000000
C	-2.9122760000	-1.2439220000	0.0000000000
C	2.9122760000	1.2439220000	0.0000000000
C	2.9122760000	-0.9584670000	0.0000000000
C	-1.2041740000	2.8491380000	0.0000000000
C	1.0668030000	2.9930650000	0.0000000000
C	-1.0668030000	-2.9930650000	0.0000000000
C	1.2041740000	-2.8491380000	0.0000000000
C	-4.3315720000	0.5347750000	0.0000000000
C	-4.2753920000	-0.8844650000	0.0000000000
C	4.2753920000	0.8844650000	0.0000000000
C	4.3315720000	-0.5347750000	0.0000000000
C	-0.8528330000	4.2581170000	0.0000000000
C	0.5728550000	4.3116520000	0.0000000000
C	-0.5728550000	-4.3116520000	0.0000000000
C	0.8528330000	-4.2581170000	0.0000000000
C	-2.5576600000	2.3748880000	0.0000000000
C	-2.4015380000	-2.5511210000	0.0000000000
C	2.5576600000	-2.3748880000	0.0000000000
C	2.4015380000	2.5511210000	0.0000000000
C	-5.3421150000	1.4618640000	0.0000000000
H	-5.1148860000	-1.5697930000	0.0000000000
H	5.1148860000	1.5697930000	0.0000000000
C	5.3421150000	-1.4618640000	0.0000000000
C	-1.8526200000	5.2155760000	0.0000000000
H	1.1868920000	5.2032860000	0.0000000000
H	-1.1868920000	-5.2032860000	0.0000000000
C	1.8526200000	-5.2155760000	0.0000000000
C	-3.5757900000	3.3205230000	0.0000000000
H	3.8159180000	-5.2639640000	-0.8664190000
C	3.5757900000	-3.3205230000	0.0000000000
H	0.0128160000	1.1177640000	0.0000000000
H	-0.0128160000	-1.1177640000	0.0000000000
C	-5.0234840000	2.9214750000	0.0000000000
H	3.8159180000	-5.2639640000	0.8664190000
C	-3.2942740000	4.8064350000	0.0000000000
H	-1.6188680000	6.2778970000	0.0000000000
H	-3.8159180000	5.2639640000	-0.8664190000
C	5.0234840000	-2.9214750000	0.0000000000
H	1.6188680000	-6.2778970000	0.0000000000
C	3.2942740000	-4.8064350000	0.0000000000
H	3.1437240000	3.3478690000	0.0000000000
H	-3.1437240000	-3.3478690000	0.0000000000
H	5.5180720000	-3.4040900000	-0.8657210000
H	5.5180720000	-3.4040900000	0.8657210000
H	6.3908000000	-1.1697060000	0.0000000000
H	-6.3908000000	1.1697060000	0.0000000000
H	-5.5180720000	3.4040900000	0.8657210000
H	-5.5180720000	3.4040900000	-0.8657210000

The quintet UKS/DFT-optimized geometry of molecule **QD-Z** is given below (BLYP/cc-pVTZ level of theory). Total electronic energy: -1374.02758163 *a.u.*

54

H	-3.8128270000	5.2880550000	0.8673450000

N	-2.0870090000	-0.0578780000	0.0000000000
N	-0.0435930000	2.1160410000	0.0000000000
N	2.0870090000	0.0578780000	0.0000000000
N	0.0435930000	-2.1160410000	0.0000000000
C	-2.9153040000	0.9745610000	0.0000000000
C	-2.9153040000	-1.2244750000	0.0000000000
C	2.9153040000	1.2244750000	0.0000000000
C	2.9153040000	-0.9745610000	0.0000000000
C	-1.2001890000	2.8469820000	0.0000000000
C	1.0663280000	2.9623310000	0.0000000000
C	-1.0663280000	-2.9623310000	0.0000000000
C	1.2001890000	-2.8469820000	0.0000000000
C	-4.3333220000	0.5580050000	0.0000000000
C	-4.2870820000	-0.8616650000	0.0000000000
C	4.2870820000	0.8616650000	0.0000000000
C	4.3333220000	-0.5580050000	0.0000000000
C	-0.8460020000	4.2430750000	0.0000000000
C	0.5798830000	4.2911280000	0.0000000000
C	-0.5798830000	-4.2911280000	0.0000000000
C	0.8460020000	-4.2430750000	0.0000000000
C	-2.5571350000	2.3869550000	0.0000000000
C	-2.4101980000	-2.5305720000	0.0000000000
C	2.5571350000	-2.3869550000	0.0000000000
C	2.4101980000	2.5305720000	0.0000000000
C	-5.3445260000	1.4882160000	0.0000000000
H	-5.1288930000	-1.5445840000	0.0000000000
H	5.1288930000	1.5445840000	0.0000000000
C	5.3445260000	-1.4882160000	0.0000000000
C	-1.8387500000	5.2224010000	0.0000000000
H	1.2014250000	5.1782790000	0.0000000000
H	-1.2014250000	-5.1782790000	0.0000000000
C	1.8387500000	-5.2224010000	0.0000000000
C	-3.5716660000	3.3354440000	0.0000000000
H	3.8128270000	-5.2880550000	-0.8673450000
C	3.5716660000	-3.3354440000	0.0000000000
H	0.0161160000	1.1018260000	0.0000000000
H	-0.0161160000	-1.1018260000	0.0000000000
C	-5.0280550000	2.9522180000	0.0000000000
H	3.8128270000	-5.2880550000	0.8673450000
C	-3.2912580000	4.8281410000	0.0000000000
H	-1.5875580000	6.2807620000	0.0000000000
H	-3.8128270000	5.2880550000	-0.8673450000
C	5.0280550000	-2.9522180000	0.0000000000
H	1.5875580000	-6.2807620000	0.0000000000
C	3.2912580000	-4.8281410000	0.0000000000
H	3.1491960000	3.3302760000	0.0000000000
H	-3.1491960000	-3.3302760000	0.0000000000
H	5.5256660000	-3.4361320000	-0.8657180000
H	5.5256660000	-3.4361320000	0.8657180000
H	6.3925380000	-1.1914850000	0.0000000000
H	-6.3925380000	1.1914850000	0.0000000000
H	-5.5256660000	3.4361320000	0.8657180000
H	-5.5256660000	3.4361320000	-0.8657180000

The quintet UKS/DFT-optimized geometry of molecule **QD-X** is given below (BLYP/cc-pVTZ level of theory). Total electronic energy: -1374.01610783 *a.u.*

54

H	-0.8662960000	1.2292740000	-6.3182840000
N	-0.0003170000	-1.5380370000	-1.4306800000
N	0.0003170000	1.5380370000	-1.4306800000
N	0.0003240000	1.5822690000	1.4179260000
N	-0.0003240000	-1.5822690000	1.4179260000
C	-0.0001910000	-1.2976390000	-2.7301460000
C	-0.0003490000	-2.9612530000	-1.2840520000
C	0.0002510000	2.9674550000	1.2350450000
C	0.0002320000	1.2761580000	2.7610790000
C	0.0001910000	1.2976390000	-2.7301460000
C	0.0003490000	2.9612530000	-1.2840520000
C	-0.0002510000	-2.9674550000	1.2350450000
C	-0.0002320000	-1.2761580000	2.7610790000
C	-0.0002140000	-2.5594910000	-3.5166060000
C	-0.0003100000	-3.5957130000	-2.5474000000
C	-0.0000000000	3.5658150000	2.5124180000
C	-0.0000170000	2.5259270000	3.4863290000
C	0.0002140000	2.5594910000	-3.5166060000
C	0.0003100000	3.5957130000	-2.5474000000
C	-0.0000000000	-3.5658150000	2.5124180000
C	0.0000170000	-2.5259270000	3.4863290000
C	-0.0000000000	0.0000000000	-3.4151420000
C	-0.0003370000	-3.5873160000	-0.0308420000
C	0.0000000000	-0.0000000000	3.4244200000
C	0.0003370000	3.5873160000	-0.0308420000
C	-0.0001860000	-2.5570940000	-4.8870130000
H	-0.0003390000	-4.6635760000	-2.7330060000
H	-0.0001940000	4.6323120000	2.7013860000
C	-0.0002140000	2.5534900000	4.8791600000
C	0.0001860000	2.5570940000	-4.8870130000
H	0.0003390000	4.6635760000	-2.7330060000
H	0.0001940000	-4.6323120000	2.7013860000
C	0.0002140000	-2.5534900000	4.8791600000
C	-0.0000000000	0.0000000000	-4.7990030000
H	0.8663490000	-1.2411210000	6.3508480000
C	0.0000000000	-0.0000000000	4.8295080000
H	-0.8667460000	-1.2291600000	-6.3181170000
H	0.8662960000	-1.2292740000	-6.3182840000
C	-0.0001590000	-1.2627310000	-5.6288430000
H	-0.8671190000	-1.2412700000	6.3502810000
C	0.0001590000	1.2627310000	-5.6288430000
H	0.0002260000	3.4818840000	-5.4621540000
H	0.8667460000	1.2291600000	-6.3181170000
C	0.0001440000	1.2692430000	5.6532510000
H	0.0004840000	-3.4963590000	5.4214900000
C	-0.0001440000	-1.2692430000	5.6532510000
H	0.0003150000	4.6756700000	-0.0240940000
H	-0.0003150000	-4.6756700000	-0.0240940000
H	0.8671190000	1.2412700000	6.3502810000
H	-0.8663490000	1.2411210000	6.3508480000
H	-0.0004840000	3.4963590000	5.4214900000
H	-0.0002260000	-3.4818840000	-5.4621540000
H	0.0010200000	0.9861030000	0.5873070000
H	-0.0010200000	-0.9861030000	0.5873070000

The septet UKS/DFT-optimized geometry of molecule **QD-X** is given below (BLYP/cc-pVTZ level of theory).

Total electronic energy: -1373.95669956 $a.u.$

54

```
H     0.8664490000    1.2315750000    6.3662210000
N    -0.0000480000   -1.5401470000    1.3972310000
N     0.0000480000    1.5401470000    1.3972310000
N     0.0000410000    1.5859890000   -1.4107610000
N    -0.0000410000   -1.5859890000   -1.4107610000
C    -0.0000210000   -1.2722610000    2.7282240000
C    -0.0000230000   -2.9255570000    1.2716990000
C    -0.0000030000    2.9671110000   -1.2348740000
C     0.0000240000    1.2696640000   -2.7579370000
C     0.0000210000    1.2722610000    2.7282240000
C     0.0000230000    2.9255570000    1.2716990000
C     0.0000030000   -2.9671110000   -1.2348740000
C    -0.0000240000   -1.2696640000   -2.7579370000
C    -0.0000000000   -2.5244110000    3.5153890000
C     0.0000020000   -3.5629940000    2.5586460000
C    -0.0000640000    3.5617570000   -2.5247580000
C    -0.0000440000    2.5202210000   -3.4882320000
C    -0.0000000000    2.5244110000    3.5153890000
C    -0.0000020000    3.5629940000    2.5586460000
C     0.0000640000   -3.5617570000   -2.5247580000
C     0.0000440000   -2.5202210000   -3.4882320000
C     0.0000000000   -0.0000000000    3.3876760000
C    -0.0000090000   -3.5864530000    0.0259170000
C    -0.0000000000    0.0000000000   -3.4128060000
C     0.0000090000    3.5864530000    0.0259170000
C     0.0000140000   -2.5378550000    4.8937310000
H     0.0000190000   -4.6308000000    2.7476300000
H    -0.0001220000    4.6274170000   -2.7185300000
C    -0.0000800000    2.5493640000   -4.8823840000
C    -0.0000140000    2.5378550000    4.8937310000
H    -0.0000190000    4.6308000000    2.7476300000
H     0.0001220000   -4.6274170000   -2.7185300000
C     0.0000800000   -2.5493640000   -4.8823840000
C     0.0000000000   -0.0000000000    4.8334510000
H    -0.8670560000   -1.2460780000   -6.3599080000
C    -0.0000000000    0.0000000000   -4.8386570000
H     0.8664370000   -1.2315640000    6.3662350000
H    -0.8664490000   -1.2315750000    6.3662210000
C     0.0000000000   -1.2575530000    5.6661940000
H     0.8669620000   -1.2460200000   -6.3600070000
C    -0.0000000000    1.2575530000    5.6661940000
H    -0.0000280000    3.4779560000    5.4443310000
H    -0.8664370000    1.2315640000    6.3662350000
C     0.0000050000    1.2684040000   -5.6622970000
H     0.0001480000   -3.4949910000   -5.4201360000
C    -0.0000050000   -1.2684040000   -5.6622970000
H    -0.0000230000    4.6738740000    0.0328830000
H     0.0000230000   -4.6738740000    0.0328830000
H    -0.8669620000    1.2460200000   -6.3600070000
H     0.8670560000    1.2460780000   -6.3599080000
H    -0.0001480000    3.4949910000   -5.4201360000
H     0.0000280000   -3.4779560000    5.4443310000
H     0.0002220000    0.9996270000   -0.5689190000
H    -0.0002220000   -0.9996270000   -0.5689190000
```

The triplet UKS/DFT-optimized geometry of molecule **QD-Y** is given below (BLYP/cc-pVTZ level of theory). Total electronic energy: -1374.02325910 *a.u.*

```
54

H      0.8531450000    6.4627750000   -1.2561500000
N      0.0040290000    1.5482210000    1.3820950000
N      0.0055280000    1.5822790000   -1.4403050000
N     -0.0055280000   -1.5822790000   -1.4403050000
N     -0.0040290000   -1.5482210000    1.3820950000
C      0.0014430000    2.8592600000    1.2622400000
C      0.0014210000    1.2864710000    2.7939560000
C      0.0002530000   -1.2867200000   -2.8239590000
C      0.0003510000   -2.9203480000   -1.2506420000
C     -0.0003510000    2.9203480000   -1.2506420000
C     -0.0002530000    1.2867200000   -2.8239590000
C     -0.0014210000   -1.2864710000    2.7939560000
C     -0.0014430000   -2.8592600000    1.2622400000
C     -0.0017830000    3.5610600000    2.5593250000
C     -0.0010210000    2.5036720000    3.5153350000
C      0.0089010000   -2.5358240000   -3.4970980000
C      0.0090030000   -3.5831630000   -2.5285230000
C     -0.0090030000    3.5831630000   -2.5285230000
C     -0.0089010000    2.5358240000   -3.4970980000
C      0.0010210000   -2.5036720000    3.5153350000
C      0.0017830000   -3.5610600000    2.5593250000
C      0.0000000000    3.5946250000    0.0107270000
C      0.0000000000   -0.0000000000    3.3656070000
C      0.0000000000   -3.5946250000    0.0107270000
C     -0.0000000000    0.0000000000   -3.3919000000
C     -0.0054870000    4.9328240000    2.5913010000
H     -0.0036510000    2.6007890000    4.5947890000
H      0.0162910000   -2.6473610000   -4.5740500000
C      0.0150350000   -4.9766570000   -2.5518710000
C     -0.0150350000    4.9766570000   -2.5518710000
H     -0.0162910000    2.6473610000   -4.5740500000
H      0.0036510000   -2.6007890000    4.5947890000
C      0.0054870000   -4.9328240000    2.5913010000
C     -0.0060490000    4.9749130000    0.0199890000
H     -0.8563320000   -6.4282540000    1.3235540000
C      0.0060490000   -4.9749130000    0.0199890000
H      0.8563320000    6.4282540000    1.3235540000
H     -0.8756620000    6.4236100000    1.3239770000
C     -0.0078380000    5.7346010000    1.3198190000
H      0.8756620000   -6.4236100000    1.3239770000
C     -0.0121200000    5.7671490000   -1.2692810000
H     -0.0208240000    5.5291780000   -3.4884900000
H     -0.8798220000    6.4594270000   -1.2498980000
C      0.0121200000   -5.7671490000   -1.2692810000
H      0.0074640000   -5.4859570000    3.5294820000
C      0.0078380000   -5.7346010000    1.3198190000
H     -0.0000000000    0.0000000000   -4.4803380000
H      0.0000000000   -0.0000000000    4.4564000000
H     -0.8531450000   -6.4627750000   -1.2561500000
H      0.8798220000   -6.4594270000   -1.2498980000
H      0.0208240000   -5.5291780000   -3.4884900000
H     -0.0074640000    5.4859570000    3.5294820000
H      0.0205540000    0.9765280000   -0.6127940000
H     -0.0205540000   -0.9765280000   -0.6127940000
```

The septet UKS/DFT-optimized geometry of molecule **QD-Y** is given below (BLYP/cc-pVTZ level of theory). Total electronic energy: -1373.95589265 *a.u.*

```
54

H      0.8728500000    6.4916460000   -1.2484890000
N     -0.0456110000    1.5292630000    1.3924980000
N     -0.0534590000    1.5520920000   -1.4393460000
N      0.0534590000   -1.5520920000   -1.4393460000
N      0.0456110000   -1.5292630000    1.3924980000
C     -0.0367570000    2.8748190000    1.2590350000
C     -0.0205420000    1.2770250000    2.7480720000
C      0.0143170000   -1.2773690000   -2.8085410000
C      0.0346410000   -2.9107940000   -1.2274450000
C     -0.0346410000    2.9107940000   -1.2274450000
C     -0.0143170000    1.2773690000   -2.8085410000
C      0.0205420000   -1.2770250000    2.7480720000
C      0.0367570000   -2.8748190000    1.2590350000
C     -0.0124130000    3.5765440000    2.5407560000
C     -0.0054820000    2.5382300000    3.4957340000
C     -0.0278300000   -2.5375610000   -3.4770610000
C     -0.0151230000   -3.5740390000   -2.4977880000
C      0.0151230000    3.5740390000   -2.4977880000
C      0.0278300000    2.5375610000   -3.4770610000
C      0.0054820000   -2.5382300000    3.4957340000
C      0.0124130000   -3.5765440000    2.5407560000
C     -0.0358490000    3.5846900000    0.0183950000
C     -0.0000000000    0.0000000000    3.3435110000
C      0.0358490000   -3.5846900000    0.0183950000
C      0.0000000000   -0.0000000000   -3.4065460000
C      0.0025780000    4.9677560000    2.5879500000
H      0.0164440000    2.6325020000    4.5763280000
H     -0.0730290000   -2.6560850000   -4.5529230000
C     -0.0460270000   -4.9710810000   -2.5435440000
C      0.0460270000    4.9710810000   -2.5435440000
H      0.0730290000    2.6560850000   -4.5529230000
H     -0.0164440000   -2.6325020000    4.5763280000
C     -0.0025780000   -4.9677560000    2.5879500000
C     -0.0077240000    4.9951600000    0.0254290000
H     -0.8692720000   -6.4754420000    1.3131870000
C      0.0077240000   -4.9951600000    0.0254290000
H      0.8692720000    6.4754420000    1.3131870000
H     -0.8646470000    6.4821390000    1.3204230000
C     -0.0000000000    5.7826830000    1.3161700000
H      0.8646470000   -6.4821390000    1.3204230000
C      0.0125640000    5.7878410000   -1.2686900000
H      0.0873250000    5.5025950000   -3.4919860000
H     -0.8608780000    6.4776430000   -1.2849020000
C     -0.0125640000   -5.7878410000   -1.2686900000
H     -0.0179190000   -5.5027050000    3.5360490000
C      0.0000000000   -5.7826830000    1.3161700000
H      0.0000000000   -0.0000000000   -4.4937530000
H     -0.0000000000    0.0000000000    4.4335690000
H     -0.8728500000   -6.4916460000   -1.2484890000
H      0.8608780000   -6.4776430000   -1.2849020000
H     -0.0873250000   -5.5025950000   -3.4919860000
H      0.0179190000    5.5027050000    3.5360490000
H     -0.1466290000    0.9341950000   -0.6313950000
H      0.1466290000   -0.9341950000   -0.6313950000
```

The triplet UKS/DFT-optimized geometry of molecule $\mathbf{QD}^{-}_{m1\mathrm{H}}$ is given below (BLYP/cc-pVTZ level of theory). Total electronic energy: -1373.48447061 *a.u.*

```
53

H     -3.8285110000    5.2772300000    0.8685870000
N     -2.1021510000   -0.0400600000    0.0000000000
N     -0.0517770000    2.0765260000    0.0000000000
N      2.1254150000    0.0921340000    0.0000000000
N      0.0847850000   -2.0761090000    0.0000000000
C     -2.9433210000    0.9662960000    0.0000000000
C     -2.9067090000   -1.2278020000    0.0000000000
C      2.9626580000    1.2452580000    0.0000000000
C      2.9287280000   -0.9512150000    0.0000000000
C     -1.2092850000    2.8155330000    0.0000000000
C      1.0590130000    2.9313370000    0.0000000000
C     -1.0315330000   -2.9718110000    0.0000000000
C      1.1587230000   -2.8268260000    0.0000000000
C     -4.3633310000    0.5339500000    0.0000000000
C     -4.2808050000   -0.8897940000    0.0000000000
C      4.3329780000    0.8697900000    0.0000000000
C      4.3632030000   -0.5502860000    0.0000000000
C     -0.8564240000    4.2147660000    0.0000000000
C      0.5690620000    4.2579760000    0.0000000000
C     -0.5898150000   -4.3161330000    0.0000000000
C      0.8340200000   -4.2830850000    0.0000000000
C     -2.5745720000    2.3750110000    0.0000000000
C     -2.3687880000   -2.5302910000    0.0000000000
C      2.5502310000   -2.3667790000    0.0000000000
C      2.4191350000    2.5343220000    0.0000000000
C     -5.3656170000    1.4690500000    0.0000000000
H     -5.1098580000   -1.5899460000    0.0000000000
H      5.1857810000    1.5407690000    0.0000000000
C      5.3609700000   -1.4959380000    0.0000000000
C     -1.8477260000    5.1963540000    0.0000000000
H      1.1929830000    5.1444970000    0.0000000000
H     -1.2192370000   -5.2002430000    0.0000000000
C      1.8310280000   -5.2216900000    0.0000000000
C     -3.5865870000    3.3252760000    0.0000000000
H      3.7942630000   -5.2484580000   -0.8679710000
C      3.5522090000   -3.3138140000    0.0000000000
H      0.0000000000    1.0522510000    0.0000000000
C     -5.0417210000    2.9358140000    0.0000000000
H      3.7942630000   -5.2484580000    0.8679710000
C     -3.3030890000    4.8154380000    0.0000000000
H     -1.5884610000    6.2543070000    0.0000000000
H     -3.8285110000    5.2772300000   -0.8685870000
C      5.0213430000   -2.9545120000    0.0000000000
H      1.6181840000   -6.2916910000    0.0000000000
C      3.2685240000   -4.7989230000    0.0000000000
H      3.1289590000    3.3617060000    0.0000000000
H     -3.1137560000   -3.3290290000    0.0000000000
H      5.4990010000   -3.4554950000   -0.8680820000
H      5.4990010000   -3.4554950000    0.8680820000
H      6.4155460000   -1.2180540000    0.0000000000
H     -6.4189640000    1.1851460000    0.0000000000
H     -5.5347940000    3.4263350000    0.8673930000
H     -5.5347940000    3.4263350000   -0.8673930000
```

The quintet UKS/DFT-optimized geometry of molecule $\mathbf{QD}^{-}_{m1\mathrm{H}}$ is given below (BLYP/cc-pVTZ level of theory). Total electronic energy: -1373.46176971 *a.u.*

```
53

H     -3.5218920000    5.5004080000    0.8680690000
N     -2.0948900000    0.0817710000    0.0000000000
N      0.0358310000    2.0686050000    0.0000000000
N      2.1177400000   -0.0450720000    0.0000000000
N     -0.0269630000   -2.0764770000    0.0000000000
C     -2.8990210000    1.1422260000    0.0000000000
C     -2.9368230000   -1.0395150000    0.0000000000
C      3.0079820000    1.0594460000    0.0000000000
C      2.8660990000   -1.1312410000    0.0000000000
C     -1.0692090000    2.8839570000    0.0000000000
C      1.1983400000    2.8448350000    0.0000000000
C     -1.1702660000   -2.8872120000    0.0000000000
C      1.0130210000   -2.8999520000    0.0000000000
C     -4.3210380000    0.7905110000    0.0000000000
C     -4.3150790000   -0.6299460000    0.0000000000
C      4.3619300000    0.6219000000    0.0000000000
C      4.3179790000   -0.8004600000    0.0000000000
C     -0.6272090000    4.2531620000    0.0000000000
C      0.7967670000    4.2052180000    0.0000000000
C     -0.8064440000   -4.2768130000    0.0000000000
C      0.6125000000   -4.3207460000    0.0000000000
C     -2.4609970000    2.5212260000    0.0000000000
C     -2.4836200000   -2.3763620000    0.0000000000
C      2.4189700000   -2.5245740000    0.0000000000
C      2.5357800000    2.3767880000    0.0000000000
C     -5.2942230000    1.7832210000    0.0000000000
H     -5.1776380000   -1.2900810000    0.0000000000
H      5.2471650000    1.2497670000    0.0000000000
C      5.2713140000   -1.7886790000    0.0000000000
C     -1.5542390000    5.3007030000    0.0000000000
H      1.4787630000    5.0480860000    0.0000000000
H     -1.4882760000   -5.1225140000    0.0000000000
C      1.5591820000   -5.3322660000    0.0000000000
C     -3.4090900000    3.5350790000    0.0000000000
H      3.5311080000   -5.4788650000   -0.8686920000
C      3.3789660000   -3.5210110000    0.0000000000
H      0.0000000000    1.0411250000    0.0000000000
C     -4.8953530000    3.2390850000    0.0000000000
H      3.5311080000   -5.4788650000    0.8686920000
C     -3.0310330000    5.0036080000    0.0000000000
H     -1.2292290000    6.3401230000    0.0000000000
H     -3.5218920000    5.5004080000   -0.8680690000
C      4.8612480000   -3.2307580000    0.0000000000
H      1.2767730000   -6.3858750000    0.0000000000
C      3.0271600000   -4.9985370000    0.0000000000
H      3.2883000000    3.1654230000    0.0000000000
H     -3.2746440000   -3.1298260000    0.0000000000
H      5.3261160000   -3.7461920000   -0.8673220000
H      5.3261160000   -3.7461920000    0.8673220000
H      6.3375740000   -1.5579890000    0.0000000000
H     -6.3589280000    1.5479880000    0.0000000000
H     -5.3570660000    3.7619590000    0.8682080000
H     -5.3570660000    3.7619590000   -0.8682080000
```

The septet UKS/DFT-optimized geometry of molecule $\mathbf{QD}^{-}_{m1\mathrm{H}}$ is given below (BLYP/cc-pVTZ level of theory). Total electronic energy: -1373.40604801 *a.u.*

```
53

H     -3.8794640000    5.2684940000    0.8682560000
N     -2.0857360000   -0.0663870000    0.0000000000
N     -0.0731620000    2.0783380000    0.0000000000
N      2.0877220000    0.1265710000    0.0000000000
N      0.0892070000   -2.0727900000    0.0000000000
C     -2.9528550000    0.9591390000    0.0000000000
C     -2.8485530000   -1.2217830000    0.0000000000
C      2.8979240000    1.2381680000    0.0000000000
C      2.9173750000   -0.9569070000    0.0000000000
C     -1.2479150000    2.8037790000    0.0000000000
C      1.0258100000    2.9419160000    0.0000000000
C     -0.9754980000   -2.9432680000    0.0000000000
C      1.2090700000   -2.8231410000    0.0000000000
C     -4.3528030000    0.5212090000    0.0000000000
C     -4.2666670000   -0.8910650000    0.0000000000
C      4.2947460000    0.8699750000    0.0000000000
C      4.3296370000   -0.5467700000    0.0000000000
C     -0.9053890000    4.2069970000    0.0000000000
C      0.5145260000    4.2718070000    0.0000000000
C     -0.5092490000   -4.3236250000    0.0000000000
C      0.9033950000   -4.2659890000    0.0000000000
C     -2.5996590000    2.3507190000    0.0000000000
C     -2.3279110000   -2.5321420000    0.0000000000
C      2.5607410000   -2.3413170000    0.0000000000
C      2.3815060000    2.5578260000    0.0000000000
C     -5.3934080000    1.4435730000    0.0000000000
H     -5.0884300000   -1.6014430000    0.0000000000
H      5.1435150000    1.5478550000    0.0000000000
C      5.3664840000   -1.4683850000    0.0000000000
C     -1.9018340000    5.1863560000    0.0000000000
H      1.1267560000    5.1666550000    0.0000000000
H     -1.1302410000   -5.2151940000    0.0000000000
C      1.9064680000   -5.2240840000    0.0000000000
C     -3.6364400000    3.3115100000    0.0000000000
H      3.8847400000   -5.2652820000   -0.8677840000
C      3.6112780000   -3.3183080000    0.0000000000
H      0.0000000000    1.0538410000    0.0000000000
C     -5.1000300000    2.9239030000    0.0000000000
H      3.8847400000   -5.2652820000    0.8677840000
C     -3.3585840000    4.7995510000    0.0000000000
H     -1.6439770000    6.2449020000    0.0000000000
H     -3.8794640000    5.2684940000   -0.8682560000
C      5.0766720000   -2.9452340000    0.0000000000
H      1.6765730000   -6.2903310000    0.0000000000
C      3.3524910000   -4.8073950000    0.0000000000
H      3.1003730000    3.3758830000    0.0000000000
H     -3.0681160000   -3.3339930000    0.0000000000
H      5.5767620000   -3.4397410000   -0.8679090000
H      5.5767620000   -3.4397410000    0.8679090000
H      6.4086940000   -1.1466330000    0.0000000000
H     -6.4371020000    1.1275540000    0.0000000000
H     -5.6031500000    3.4108770000    0.8682270000
H     -5.6031500000    3.4108770000   -0.8682270000
```

The triplet UKS/DFT-optimized geometry of molecule $\mathbf{QD}^{2-}_{m2H}$ is given below (BLYP/cc-pVTZ level of theory). Total electronic energy: -1372.79600760 *a.u.*

```
52

H     -6.3976320000    1.2327270000    0.8692120000
N     -1.4883600000   -1.4808950000    0.0000000000
N     -1.4883600000    1.4808950000    0.0000000000
N      1.4883600000    1.4808950000    0.0000000000
N      1.4883600000   -1.4808950000    0.0000000000
C     -2.7911830000   -1.2867080000    0.0000000000
C     -1.2856900000   -2.8825860000    0.0000000000
C      1.2856900000    2.8825860000    0.0000000000
C      2.7911830000    1.2867080000    0.0000000000
C     -2.7911830000    1.2867080000    0.0000000000
C     -1.2856900000    2.8825860000    0.0000000000
C      1.2856900000   -2.8825860000    0.0000000000
C      2.7911830000   -1.2867080000    0.0000000000
C     -3.5540610000   -2.5670810000    0.0000000000
C     -2.5385990000   -3.5676740000    0.0000000000
C      2.5385990000    3.5676740000    0.0000000000
C      3.5540610000    2.5670810000    0.0000000000
C     -3.5540610000    2.5670810000    0.0000000000
C     -2.5385990000    3.5676740000    0.0000000000
C      2.5385990000   -3.5676740000    0.0000000000
C      3.5540610000   -2.5670810000    0.0000000000
C     -3.4897740000    0.0000000000    0.0000000000
C      0.0000000000   -3.4731380000    0.0000000000
C      3.4897740000   -0.0000000000    0.0000000000
C      0.0000000000    3.4731380000    0.0000000000
C     -4.9318920000   -2.5649390000    0.0000000000
H     -2.6866220000   -4.6452540000    0.0000000000
H      2.6866220000    4.6452540000    0.0000000000
C      4.9318920000    2.5649390000    0.0000000000
C     -4.9318920000    2.5649390000    0.0000000000
H     -2.6866220000    4.6452540000    0.0000000000
H      2.6866220000   -4.6452540000    0.0000000000
C      4.9318920000   -2.5649390000    0.0000000000
C     -4.8738240000    0.0000000000    0.0000000000
H      6.3976320000   -1.2327270000   -0.8692120000
C      4.8738240000   -0.0000000000    0.0000000000
H     -6.3976320000   -1.2327270000    0.8692120000
H     -6.3976320000   -1.2327270000   -0.8692120000
C     -5.6959600000   -1.2712150000    0.0000000000
H      6.3976320000   -1.2327270000    0.8692120000
C     -5.6959600000    1.2712150000    0.0000000000
H     -5.5082390000    3.4943840000    0.0000000000
H     -6.3976320000    1.2327270000   -0.8692120000
C      5.6959600000    1.2712150000    0.0000000000
H      5.5082390000   -3.4943840000    0.0000000000
C      5.6959600000   -1.2712150000    0.0000000000
H      0.0000000000    4.5676170000    0.0000000000
H     -0.0000000000   -4.5676170000    0.0000000000
H      6.3976320000    1.2327270000   -0.8692120000
H      6.3976320000    1.2327270000    0.8692120000
H      5.5082390000    3.4943840000    0.0000000000
H     -5.5082390000   -3.4943840000   -0.0000000000
```

The quintet UKS/DFT-optimized geometry of molecule $\mathbf{QD}_{m2\mathrm{H}}^{2-}$ is given below (BLYP/cc-pVTZ level of theory). Total electronic energy: -1372.77426750 *a.u.*

```
52

H     -6.4160680000    1.2419920000    0.8695230000
N     -1.4960550000   -1.4748220000    0.0000000000
N     -1.4960550000    1.4748220000    0.0000000000
N      1.4960550000    1.4748220000    0.0000000000
N      1.4960550000   -1.4748220000    0.0000000000
C     -2.8092200000   -1.2865560000    0.0000000000
C     -1.2851640000   -2.8568660000    0.0000000000
C      1.2851640000    2.8568660000    0.0000000000
C      2.8092200000    1.2865560000    0.0000000000
C     -2.8092200000    1.2865560000    0.0000000000
C     -1.2851640000    2.8568660000    0.0000000000
C      1.2851640000   -2.8568660000    0.0000000000
C      2.8092200000   -1.2865560000    0.0000000000
C     -3.5599250000   -2.5591310000    0.0000000000
C     -2.5454810000   -3.5568570000    0.0000000000
C      2.5454810000    3.5568570000    0.0000000000
C      3.5599250000    2.5591310000    0.0000000000
C     -3.5599250000    2.5591310000    0.0000000000
C     -2.5454810000    3.5568570000    0.0000000000
C      2.5454810000   -3.5568570000    0.0000000000
C      3.5599250000   -2.5591310000    0.0000000000
C     -3.5077610000    0.0000000000    0.0000000000
C      0.0000000000   -3.4508210000    0.0000000000
C      3.5077610000   -0.0000000000    0.0000000000
C      0.0000000000    3.4508210000    0.0000000000
C     -4.9492100000   -2.5732250000    0.0000000000
H     -2.6902600000   -4.6356900000    0.0000000000
H      2.6902600000    4.6356900000    0.0000000000
C      4.9492100000    2.5732250000    0.0000000000
C     -4.9492100000    2.5732250000    0.0000000000
H     -2.6902600000    4.6356900000    0.0000000000
H      2.6902600000   -4.6356900000    0.0000000000
C      4.9492100000   -2.5732250000    0.0000000000
C     -4.8916690000    0.0000000000    0.0000000000
H      6.4160680000   -1.2419920000   -0.8695230000
C      4.8916690000   -0.0000000000    0.0000000000
H     -6.4160680000   -1.2419920000    0.8695230000
H     -6.4160680000   -1.2419920000   -0.8695230000
C     -5.7163660000   -1.2752510000    0.0000000000
H      6.4160680000   -1.2419920000    0.8695230000
C     -5.7163660000    1.2752510000    0.0000000000
H     -5.5195980000    3.5056190000    0.0000000000
H     -6.4160680000    1.2419920000   -0.8695230000
C      5.7163660000    1.2752510000    0.0000000000
H      5.5195980000   -3.5056190000    0.0000000000
C      5.7163660000   -1.2752510000    0.0000000000
H      0.0000000000    4.5457560000    0.0000000000
H     -0.0000000000   -4.5457560000    0.0000000000
H      6.4160680000    1.2419920000   -0.8695230000
H      6.4160680000    1.2419920000    0.8695230000
H      5.5195980000    3.5056190000    0.0000000000
H     -5.5195980000   -3.5056190000   -0.0000000000
```

The septet UKS/DFT-optimized geometry of molecule $\mathbf{QD}_{m2\mathrm{H}}^{2-}$ is given below (BLYP/cc-pVTZ level of theory). Total electronic energy: -1372.71341886 *a.u.*

```
52

H     -6.4461950000    1.2464280000    0.8694630000
N     -1.4777700000   -1.4795080000    0.0000000000
N     -1.4777700000    1.4795080000    0.0000000000
N      1.4777700000    1.4795080000    0.0000000000
N      1.4777700000   -1.4795080000    0.0000000000
C     -2.8101900000   -1.2695360000    0.0000000000
C     -1.2803610000   -2.8421230000    0.0000000000
C      1.2803610000    2.8421230000    0.0000000000
C      2.8101900000    1.2695360000    0.0000000000
C     -2.8101900000    1.2695360000    0.0000000000
C     -1.2803610000    2.8421230000    0.0000000000
C      1.2803610000   -2.8421230000    0.0000000000
C      2.8101900000   -1.2695360000    0.0000000000
C     -3.5641520000   -2.5370650000    0.0000000000
C     -2.5580690000   -3.5399470000    0.0000000000
C      2.5580690000    3.5399470000    0.0000000000
C      3.5641520000    2.5370650000    0.0000000000
C     -3.5641520000    2.5370650000    0.0000000000
C     -2.5580690000    3.5399470000    0.0000000000
C      2.5580690000   -3.5399470000    0.0000000000
C      3.5641520000   -2.5370650000    0.0000000000
C     -3.4894570000    0.0000000000    0.0000000000
C      0.0000000000   -3.4590350000    0.0000000000
C      3.4894570000   -0.0000000000    0.0000000000
C      0.0000000000    3.4590350000    0.0000000000
C     -4.9541440000   -2.5600980000    0.0000000000
H     -2.7108010000   -4.6181800000    0.0000000000
H      2.7108010000    4.6181800000    0.0000000000
C      4.9541440000    2.5600980000    0.0000000000
C     -4.9541440000    2.5600980000    0.0000000000
H     -2.7108010000    4.6181800000    0.0000000000
H      2.7108010000   -4.6181800000    0.0000000000
C      4.9541440000   -2.5600980000    0.0000000000
C     -4.9162070000    0.0000000000    0.0000000000
H      6.4461950000   -1.2464280000   -0.8694630000
C      4.9162070000   -0.0000000000    0.0000000000
H     -6.4461950000   -1.2464280000    0.8694630000
H     -6.4461950000   -1.2464280000   -0.8694630000
C     -5.7385080000   -1.2706060000    0.0000000000
H      6.4461950000   -1.2464280000    0.8694630000
C     -5.7385080000    1.2706060000    0.0000000000
H     -5.5111800000    3.5008160000    0.0000000000
H     -6.4461950000    1.2464280000   -0.8694630000
C      5.7385080000    1.2706060000    0.0000000000
H      5.5111800000   -3.5008160000    0.0000000000
C      5.7385080000   -1.2706060000    0.0000000000
H      0.0000000000    4.5523630000    0.0000000000
H     -0.0000000000   -4.5523630000    0.0000000000
H      6.4461950000    1.2464280000   -0.8694630000
H      6.4461950000    1.2464280000    0.8694630000
H      5.5111800000    3.5008160000    0.0000000000
H     -5.5111800000   -3.5008160000   -0.0000000000
```

References

(1) Frisch, M. J. *et al.* Gaussian~16 Revision B.01. 2016; Gaussian Inc. Wallingford CT.

(2) Becke, A. D. Density-functional exchange-energy approximation with correct asymptotic behavior. *Phys. Rev. A* **1988**, *38*, 3098–3100.

(3) Lee, C.; Yang, W.; Parr, R. G. Development of the Colle-Salvetti correlation-energy formula into a functional of the electron density. *Phys. Rev. B* **1988**, *37*, 785–789.

(4) Woon, D. E.; Dunning, J., Thom H. Gaussian basis sets for use in correlated molecular calculations. V. Core-valence basis sets for boron through neon. *J. Chem. Phys.* **1995**, *103*, 4572–4585.

(5) Barca, G. M. J. *et al.* Recent developments in the general atomic and molecular electronic structure system. *J. Chem. Phys.* **2020**, *152*, 154102.

(6) Zahariev, F. *et al.* The General Atomic and Molecular Electronic Structure System (GAMESS): Novel Methods on Novel Architectures. *J. Chem. Theory Comput.* **2023**, *19*, 7031–7055.

(7) Cheung, L. M.; Sundberg, K. R.; Ruedenberg, K. Dimerization of carbene to ethylene. *J. Am. Chem. Soc.* **1978**, *100*, 8024–8025.

(8) Cheung, L. M.; Sundberg, K. R.; Ruedenberg, K. Electronic rearrangements during chemical reactions. II. Planar dissociation of ethylene. *Int. J. Quantum Chem.* **1979**, *16*, 1103–1139.

(9) Roos, B. O.; Taylor, P. R.; Sigbahn, P. E. M. A complete active space SCF method (CASSCF) using a density matrix formulated super-CI approach. *J. Chem. Phys.* **1980**, *48*, 157–173.

(10) Siegbahn, P.; Heiberg, A.; Roos, B.; Levy, B. A Comparison of the Super-CI and the Newton-Raphson Scheme in the Complete Active Space SCF Method. *Phys. Scr.* **1980**, *21*, 323–327.

(11) Johnson, R. P.; Schmidt, M. W. The sudden polarization effect: MC-SCF calculations on planar and 90.degree. twisted methylenecyclopropene. *J. Am. Chem. Soc.* **1981**, *103*, 3244–3249.

(12) Siegbahn, P. E. M.; Almlöf, J.; Heiberg, A.; Roos, B. O. The complete active space SCF (CASSCF) method in a Newton–Raphson formulation with application to the HNO molecule. *J. Chem. Phys.* **1981**, *74*, 2384–2396.

(13) Feller, D. F.; Schmidt, M. W.; Ruedenberg, K. Concerted dihydrogen exchange between ethane and ethylene. SCF and FORS calculations of the barrier. *J. Am. Chem. Soc.* **1982**, *104*, 960–967.

(14) Pulay, P.; Hamilton, T. P. UHF natural orbitals for defining and starting MC-SCF calculations. *J. Chem. Phys.* **1988**, *88*, 4926–4933.

(15) Bofill, J. M.; Pulay, P. The unrestricted natural orbital–complete active space (UNO–CAS) method: An inexpensive alternative to the complete active space–self-consistent-field (CAS–SCF) method. *J. Chem. Phys.* **1989**, *90*, 3637–3646.

(16) Tóth, Z.; Pulay, P. Comparison of Methods for Active Orbital Selection in Multiconfigurational Calculations. *J. Chem. Theory Comput.* **2020**, *16*, 7328–7341.

(17) Lowdin, P.-O. Present Situation of Quantum Chemistry. *J. Phys. Chem.* **1957**, *61*, 55–68.

(18) Yamaguchi, K. The electronic structures of biradicals in the unrestricted Hartree-Fock approximation. *Chem. Phys. Lett.* **1975**, *33*, 330–335.

(19) Betkhoshvili, S.; Moreira, I. d. P. R.; Poater, J.; Bofill, J. M. Algorithmic Design of Metal-Free Fully π-Conjugated Organic Polyradicals with Any Ground-State Multiplicity. *J. Phys. Chem. C* **2025**, *129*, 4464–4480.

(20) Betkhoshvili, S.; Poater, J.; Moreira, I. d. P. R.; Bofill, J. M. Fully Conjugated Heteroatomic Non- and Quasi-Alternant Polyradicals. *Chemistry* **2025**, *7*.
